# Assessment of the Binding Affinity of Covalent Inhibitors of Cruzain and Cathepsin L Using Relative Binding Free Energy

**DOI:** 10.1002/cmdc.70508

**Published:** 2026-09-27

**Authors:** Yan Carvalho Magno, Luiz Fernando Barbosa, Maycon Vinicius Damasceno de Oliveira, Jerônimo Lameira, Carlos A. Montanari

**Affiliations:** ^1^ Laboratório de Planejamento e Desenvolvimento de Fármacos Instituto de Ciências Exatas e Naturais Universidade Federal do Pará Belém Pará Brazil; ^2^ Medicinal and Biological Chemistry Group São Carlos Institute of Chemistry University of São Paulo São Carlos São Paulo Brazil

**Keywords:** biochemistry, cathepsin L, Chagas disease, chemistry, covalent bond, cysteine protease, molecular dynamics, nitrile, protease

## Abstract

Cysteine proteases are recognized as relevant therapeutic targets in both parasitic diseases and cancer due to their essential biological functions. Among these enzymes, cruzain from *Trypanosoma cruzi* is an important molecular target for the treatment of Chagas disease. Likewise, human cathepsin L (hCatL) is a potential target for anticancer drug development. Both enzymes share conserved catalytic features, particularly the cysteine–histidine catalytic dyad, which enables interaction with nitrile‐based reversible covalent inhibitors. Here, six nitrile‐derived inhibitors (**Neq1171.1, Neq1172.1, Neq1181.1, Neq1182.1, Neq1268, and Neq1276**) had their cruzain and hCatL inhibition constants, *K*_i_, determined. Besides, we have used molecular dynamics simulation and alchemical free energy calculations for evaluating the overall relative binding free energies of these inhibitors. The calculated free energy differences closely reproduced experimental trends, confirming the reliability of the computational protocol. These findings demonstrate that thermodynamic integration is an effective strategy for predicting relative binding affinities and provides valuable molecular‐level insights for the rational optimization of cysteine protease inhibitors with potential applications in Chagas disease and cancer therapy.

## Graphical abstract

Six nitrile‐based covalent inhibitors are evaluated against cruzain and hCatL. Thermodynamic integration accurately predicts relative binding affinities by modeling the covalent adduct state. We identify a structural difference in the S2 subsite that governs ligand selectivity, offering valuable insights for drug discovery.

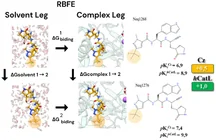

## 1 Introduction

Cysteine protease enzymes have been extensively investigated as therapeutic targets across various pathological contexts, including neglected diseases and cancer. In this scenario, cruzain (Cz), the main protease of the protozoan *Trypanosoma cruzi*, plays a fundamental role in processes essential for the parasite’s survival and replication, including cell invasion and degradation of host proteins. For this reason, Cz is considered a key target for the development of new drugs against Chagas disease, which still represents a significant public health problem [1, 2, 3]. Globally, the World Health Organization (WHO) estimated in 2026 that 8 million people are infected, with over 100 million individuals at risk. The disease currently causes approximately 10,000 deaths annually. In areas where vector transmission is controlled, and globally, the main route of infection is now transmission during pregnancy or birth, which occurs in approximately 3%–5% of pregnancies [4, 5].

In contrast, the enzyme human cathepsin L (hCatL), also a protease and a human lysosomal protein belonging to the papain family, has been associated with tumor progression, invasion, and metastasis. The overexpression of this enzyme in various types of cancer suggests its active participation in extracellular matrix remodeling and facilitation of tumor cell dissemination, making it a promising target for the development of antitumor agents [2, 6, 7, 8, 9].

From a structural point of view, both Cz and cathepsin L share conserved characteristics in the active site, including the presence of a catalytic dyad composed of the amino acids cysteine   and histidine. This arrangement favors the activation of the thiol group of cysteine, allowing nucleophilic attack on substrates or electrophilic groups present in inhibitors. In this sense, compounds containing nitriles are capable of forming thioimidate‐type adducts with catalytic cysteine, maintaining a dynamic equilibrium between the bound and unbound states [1, 2, 10, 11].

The process of covalent enzyme inhibition (Figure 1) is generally described by a two‐step mechanism, in which the inhibitor initially forms a reversible noncovalent complex with the enzyme, followed by the conversion of this intermediate into a covalent adduct via a high‐energy transition state. The formation of this state reflects the structural reorganization necessary for the establishment of the covalent bond with nucleophilic residues of the active site, culminating in a more stable complex, which may be reversible or irreversible depending on the nature of the bond formed. In this context, the efficiency of the inhibitor is determined not only by the initial affinity but also by the activation barrier and the chemical reactivity of the system [10, 12, 13].

**FIGURE 1:**
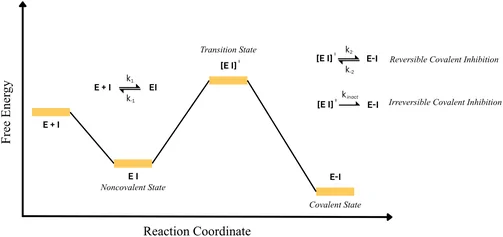
Representative free energy diagram for covalent inhibitors. The scheme shows the two‐stage binding process: the reversible formation of a noncovalent complex and the subsequent chemical reaction leading to a covalent bond formation. Rates for reversible and irreversible mechanisms are indicated.

Accurately predicting absolute binding affinities for covalent inhibitors remains challenging due to the complexity of bond formation and associated thermodynamic contributions. In this context, relative free energy approaches such as alchemical transformations offer a practical and robust framework for comparing the binding affinities of structurally related ligands. Recently, we demonstrated the applicability of free energy perturbation (FEP) [14] methods for predicting the relative binding affinities of nitrile‐based reversible covalent inhibitors targeting hCatL. Our results indicate that the covalent adduct state plays a dominant role in determining binding affinity, highlighting the importance of explicitly modeling the covalent complex to obtain reliable insights [10, 12].

Here, thermodynamic integration (TI) was used to predict the relative binding of six nitrile‐based inhibitors (Figure 2). Compounds **Neq1268** and **Neq1276** were previously reported [15], while **Neq1171.1** **,** **Neq1172.1**, and **Neq1182.1** were reported in reference [16]. **Neq1181.1** is reported herein for the first time (Figure 2). These approaches allow not only for more accurate estimation of free binding energies but also for a more detailed understanding of the molecular factors governing the recognition and stabilization of enzyme‐inhibitor complexes. This contributes to the rational design of compounds with potential application in both the treatment of Chagas disease and the development of antitumor therapeutic strategies.

**FIGURE 2:**
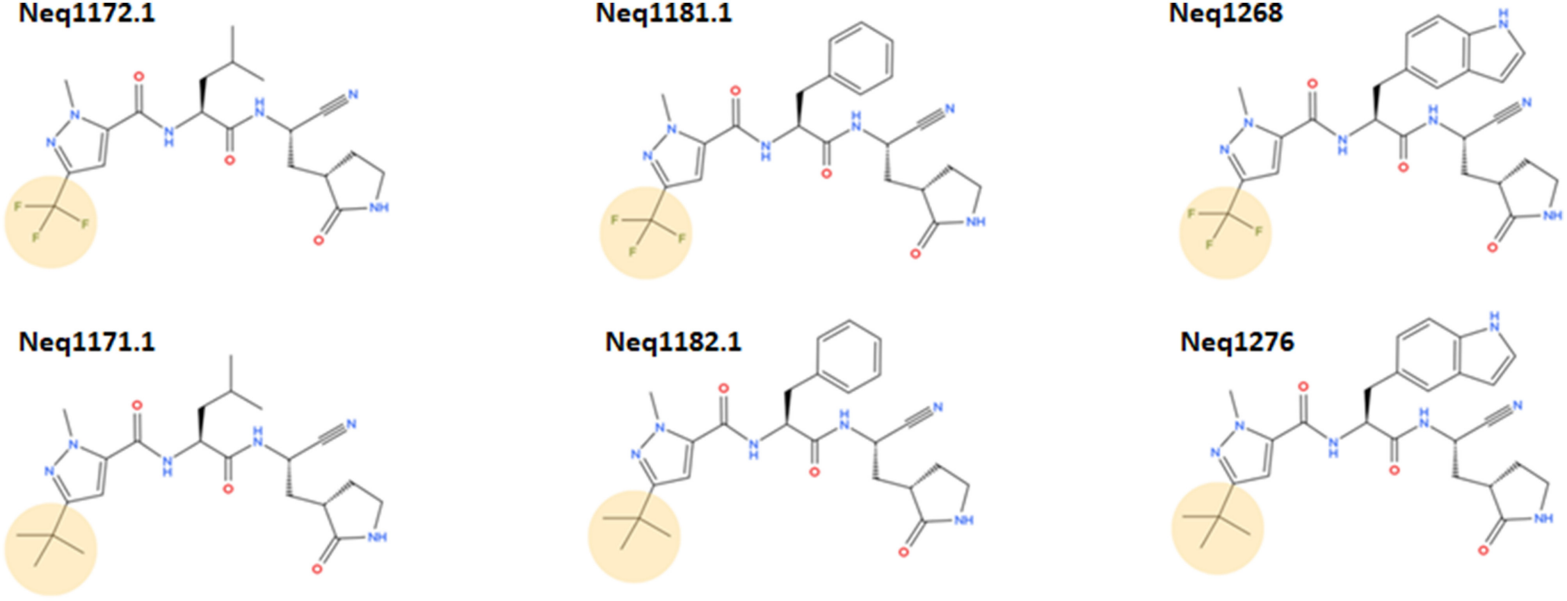
Chemical structures of nitrile‐based reversible covalent inhibitors. The circles indicate the groups selected for alchemical transformation.

## 2 Methods

### 2.1 Experimental Kinetics and Synthesis

The investigated ligands inhibit Cz and hCatL through the formation of a covalent thioimidate adduct with the catalytic residue Cys. In this study, the nitrile‐based inhibitors **Neq1171.1**, **Neq1172.1**, **Neq1181.1**, **Neq1182.1**, **Neq1268**, and **Neq1276** were evaluated against Cz and hCatL by enzymatic kinetic assays. The synthesis of **Neq1181.1** is also reported in Supporting Information.

### 2.2 Biochemical Assays

The recombinant Cz was expressed and purified as previously described [1]. hCatL was purchased from Enzo Life Sciences. The enzymatic activity was evaluated by fluorimetric assays using a Biotek Synergy HT plate reader monitoring the hydrolysis rate of the fluorogenic substrate Z‐Phe‐Arg‐7‐amido‐4‐methylcoumarin (Z‐FR‐MCA, Sigma–Aldrich) with fluorescence excitation at 360 nm and emission at 460 nm. The enzymes were activated in an assay buffer of 100 mM sodium acetate, 300 mM NaCl, 5 mM EDTA containing 7 mM DTT (Sigma–Aldrich), and 0.014% of Triton X‐100 (Sigma–Aldrich) at different times at 0 °C (Table 1).

**TABLE 1:** Parameters used in the kinetics assays for Cz and hCatL.

Enzyme	[E], nM	Substrate	Assay buffer	Time	*K*_M_, µM
Cz	0.15	Z‐FR‐MCA	100 mM sodium acetate pH 5.5	15 min	4.6 ± 0.2
hCatL	1.9	Z‐FR‐MCA	100 mM sodium acetate pH 5.5	20 min	3.0 ± 0.2

Enzyme kinetics assays were evaluated in black 96‐well plates (Corning) containing 200 µL of assay buffer constituted by 100 mM sodium acetate, 300 mM NaCl, 5 mM EDTA, 0.014% (v/v) Triton X‐100, 5 mM 1,4‐dithiothreitol (DTT), and 4% v/v dimethyl sulfoxide (DMSO). For the hCatL assays, the final enzyme concentration ([E]) was 1.9 nM, and for the Cz assays, the final concentration was 0.15 nM (Table 1).

For Michaelis–Menten constant (*K*_M_) determination, eight substrate concentrations (Z‐FR‐MCA) were prepared using a 0.5‐fold serial dilution starting from an initial concentration of 30 µM. *K*_M_ values were determined by nonlinear regression according to the Michaelis–Menten equation:

(1) $$v=\frac{V_{\max}.\left[S\right]}{K_{M}+[S]}$$

For *K*_*i*_ determination, the substrate concentration was fixed and kept equal to the *K*_M_ value, [S] = *K*_M_. Seven inhibitor solutions were prepared with a 0.5‐fold serial dilution. A final concentration of 4% v/v of DMSO was added to the last well without inhibitor. Initial inhibitor concentrations ranged from 10 µM to 10 nM for curve fitting in case the inhibitor behaves as a strong inhibitor. Inhibitors were preincubated with the enzyme solution for 2 min before initiating the reaction by substrate addition. The conversion rate was monitored for 5 min.

Visual inspection and a prereading of plate wells were performed to check for possible precipitation and background fluorescence. None of the substances displayed significant fluorescence under assay conditions.

The apparent inhibition constant (*K*_*i*_^app^) was determined by nonlinear regression using the following equation:

(2) $$V_{s}=\frac{v_{0}}{1+\frac{\left[I\right]}{K_{i}^{\mathrm{app}}}}$$

where *V*_*s*_ is the steady‐state velocity, *v*_0_ is the velocity in the absence of inhibitor, and [I] is the inhibitor concentration. The inhibition constant *K*_*i*_ was calculated by correcting *K*_*i*_^app^ using the following equation:

(3) $$K_{i}=\frac{K_{i}^{\mathrm{app}}}{1+\frac{\left[S\right]}{K_{M}}}$$

where [S] is the substrate concentration and *K*_M_ is the Michaelis–Menten constant. All *K*_*i*_ values were converted to p*K*_*i*_ by p*K*_*i*_ = −log (*K*_*i*_). A control measurement with the covalent reversible inhibitor Neq0570 [1] was done for each setup plate. All experiments were performed in triplicate. Details are provided as Supporting Information.

### 2.3 Protocol for the Preparation of Systems and Molecular Dynamics Simulations

The crystal structures of human cathepsin L (hCatL; PDB ID: 7QKB) [17] and Cz (PDB ID: 4QH6) [18] were used as starting points for the computational simulations. In the hCatL structure, the co‐crystallized inhibitor GC‐376 was retained as the reference ligand, whereas in the Cz structure the bound nitrile inhibitor, reported as compound 5 in the original crystallographic study and identified in the PDB file by the ligand code 33L, was used as the reference for the binding site definition [17, 18]. Protonation states of all ionizable residues were assigned using PROPKA [19]. Then, the covalent adduct described by the catalytic cysteine residue and the electrophilic center of the nitrile inhibitor was explicitly constructed by defining a new bonded topology within the LEaP module, resulting in a single unified residue representing the protein–ligand covalent complex.

The ff14SB force field [20] was used to describe the protein residues, whereas the parameters for the nitrile‐based inhibitors (**Neq1268**, **Neq1276**, **Neq1171.1**, **Neq1172.1**, **Neq1181.1**, and **Neq1182.1**) were generated according to the AMBER parameterization protocol. Note that the nonstandard moiety was parametrized using the GAFF force field [21], with RESP‐derived atomic partial charges obtained at the HF/6‐31G∗ level of theory using Gaussian09 [22]. The resulting covalent complex was then incorporated into the full system topology for subsequent molecular dynamics (MD) simulations in AMBER22 [23].

All complexes were solvated in explicit TIP3P [24] water molecules using an octahedral water box with a 12 Å buffer. Na^+^ counterions were added when necessary to ensure electroneutrality of the systems. Energy minimization was carried out in four sequential steps. In all stages, AMBER initially applied the steepest descent algorithm followed by conjugate gradient minimization according to the ncyc and maxcyc parameters. In the first step, only water molecules and counterions were allowed to move (ibelly = 1), using 8000 minimization steps (4000 steepest descent + 4000 conjugate gradient) with a drms convergence criterion of 0.001 kcal·mol^−1^·Å^−1^. In the second step, only the hydrogen atoms of the protein and inhibitors were minimized for 5000 steps (2000 steepest descent + 3000 conjugate gradient). In the third step, the hydrogen atoms together with the solvent molecules were minimized again for 8000 steps under the same convergence criterion. Finally, a complete minimization of the entire system (ibelly = 0) was performed without positional restraints using 10,000 steps (5000 + 5000). This gradual relaxation procedure allowed the systems to equilibrate smoothly before the heating and equilibration stages.

The systems were subsequently heated from 0 to 313.15 K over 200 ps of MD under constant volume conditions, applying positional restraints of 5.0 kcal·mol^−1^·Å^−2^ to the complexes. Equilibration was then performed for 500 ps of unrestrained MD at constant pressure (1 bar). During this stage, the temperature was maintained at 313.15 K using a Langevin thermostat with a collision frequency of 2 ps^−1^, while pressure control was achieved using a Berendsen barostat under isotropic conditions. A nonbonded interaction cutoff of 10 Å was applied, and long‐range electrostatic interactions were treated using the Particle Mesh Ewald (PME) [23] method. For each protein–inhibitor complex, three independent MD simulations of 100 ns each were carried out.

### 2.4 Thermodynamics Integration

In this study, we explored the transformation of a trifluoromethyl group into a *tert*‐butyl group (Figure 3). This alchemical change was evaluated for three specific ligand pairs in both enzymes: **Neq1172.1** → **Neq1171.1**, **Neq1181.1** → **Neq1182.1**, and **Neq1268** → **Neq1276**. Simulations were conducted using the GPU‐accelerated pmemd.cuda engine of AMBER22 [25, 26]. To obtain the ΔΔ*G* values, 11 discrete *λ* windows (from 0.0 to 1.0, with a step of 0.1) were employed. For each window, three independent replicates were performed, each consisting of 1 ns of production simulation. A dual‐leg alchemical thermodynamic cycle was constructed, comprising the transformation of the ligand in the solvated protein binding site (Δ*G*_complex_ ) and in bulk aqueous solution (Δ*G*_solvent_ ). For both the complex and solvent thermodynamic environments, the ⟨∂*V*/∂*λ*⟩_*λ* _ gradients were extracted from the AMBER production outputs prior to numerical integration. To prevent endpoint singularities during the appearance and disappearance of atoms, softcore potentials were applied to both electrostatic and van der Waals (vdW) interactions. A concerted transformation scheme was utilized, wherein all nonbonded interactions involving softcore regions were scaled simultaneously with *λ*. The softcore parameters were set to *α* = 0.5 for the Lennard–Jones potential and *β* = 1.0 for the Coulomb potential. Furthermore, tail smoothing was activated for the softcore nonbonded potentials, acting between 8.0 and 10.0 Å. The SHAKE [27] algorithm was employed to constrain bonds involving hydrogen atoms, with constraints automatically removed between bonds containing common and softcore atoms to maintain integration stability. Finally, term‐by‐term ∂*V*/∂*λ* data were logged at each step for the subsequent numerical integration.

**FIGURE 3:**
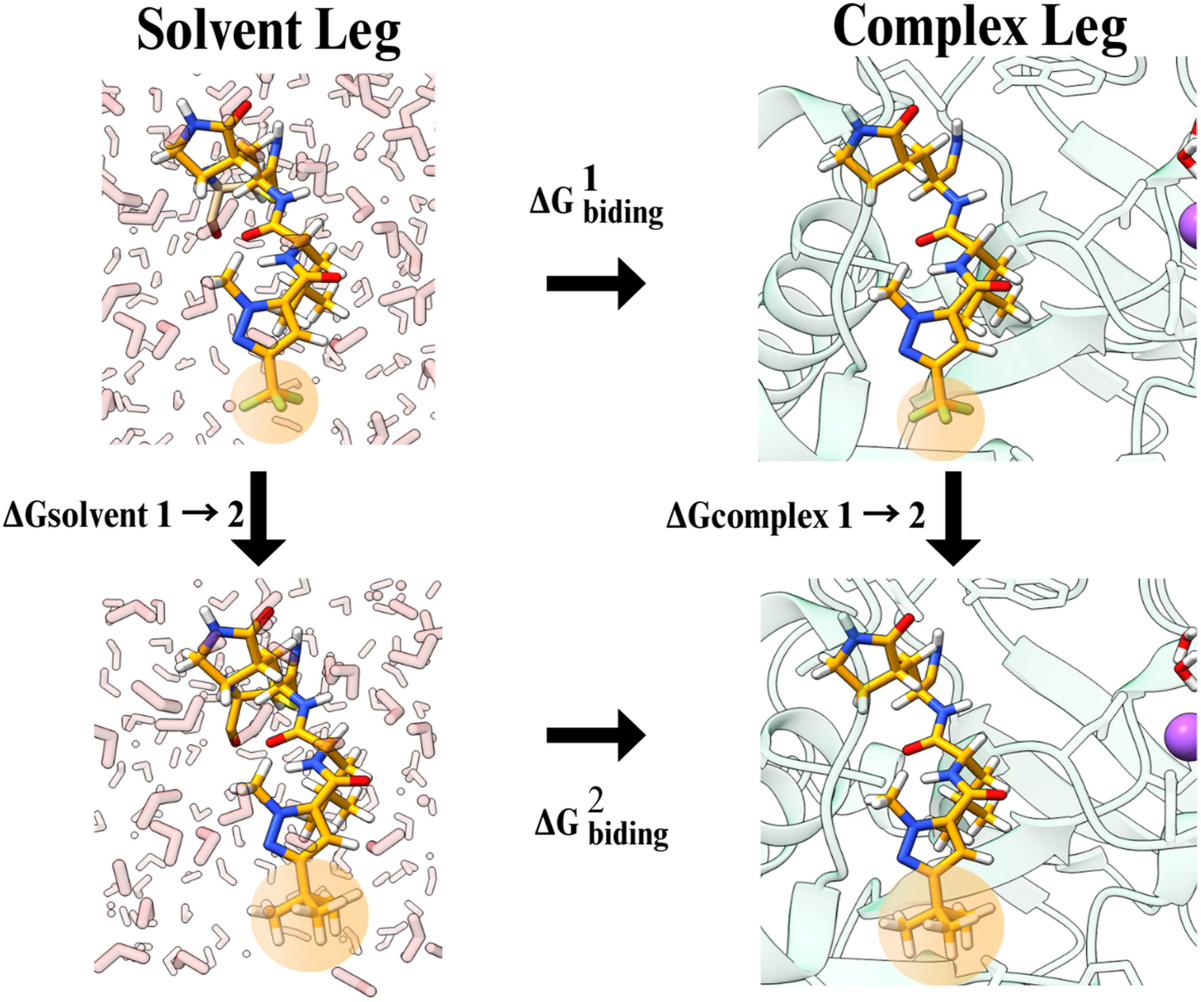
Schematic representation of the thermodynamic cycle used to calculate the relative binding free energy (ΔΔ*G*_binding_) between two ligands via alchemical thermodynamic integration (TI). The horizontal arrows represent the physical binding processes of Ligand 1 (Δ*G* ^1^_binding_) and Ligand 2 (Δ*G* ^2^_binding_) from the aqueous solution to the protein active site. The vertical arrows denote the alchemical transformations, where Ligand 1 is computationally mutated into Ligand 2 in two distinct environments: the unbound state in water (Δ*G*_solvent_) and the protein‐bound state (Δ*G*_complex_). Since free energy is a state function, the relative binding affinity is calculated by the difference between the alchemical legs according to the equation ΔΔ*G* = Δ*G* ^2^_binding_ – Δ*G* ^1^_binding_ = Δ*G*_complex_ – Δ*G*_solvent_.

The calculation of relative binding free energies (ΔΔ*G*) and the associated statistical analyses were performed using the alchemlyb v.2.5.0 Python library [28]. The free energy differences (Δ*G*) for each alchemical transformation were integrated using the TI [29] estimator. The final relative binding free energy was calculated using the standard thermodynamic cycle: ΔΔ*G* = Δ*G*_complex _ − Δ*G*_solvent_ . The reported ΔΔ*G* values represent the mean across the three replicas, with uncertainties expressed as the standard error of the mean (SEM) in kcal/mol. To ensure reproducibility and statistical robustness, all simulations were performed using three independent replicas.

## 3 Results and Discussion

### 3.1 Experimental Kinetics and MD Simulations

Here, six nitrile‐based inhibitors—**Neq1171.1**, **Neq1172.1**, **Neq1181.1**, **Neq1182.1**, **Neq1268,** and **Neq1276** (Figure 2)—were evaluated through enzyme kinetic assay against hCatL and Cz. The inhibition curves for each compound are provided in the supporting information against both Cz and hCatL (Figure S5). For the computational predictions using TI, the calculated integration curves are depicted in Figures S10, S21, S32, S43, S54, and S65. The experimentally measured p*K*_*i*_ values are depicted in Table 2.

**TABLE 2:** Experimental inhibitory potency (p*K*_*i*_) of the Neq series against cruzain and hCatL.

Compound	*K*_*i*_^Cz^, µM	p*K*_*i*_^Cz^	*K*_*i*_^hCatL^, µM	p*K*_*i*_^hCatL^
Neq1172.1	0.0794 ± 0.0060	7.1 ± 0.03	0.0505 ± 0.0060	7.3 ± 0.03
Neq1171.1	0.0116 ± 0.0016	7.9 ± 0.03	0.0113 ± 0.0046	8.0 ± 0.01
Neq1181.1	0.2879 ± 0.0303	6.5 ± 0.01	0.0486 ± 0.0038	7.3 ± 0.03
Neq1182.1	0.0520 ± 0.0150	7.3 ± 0.01	0.0033 ± 0.0006	8.5 ± 0.02
Neq1268	0.1117 ± 0.0039	6.9 ± 0.03	0.0013 ± 0.0004	8.9 ± 0.01
Neq1276	0.0400 ± 0.0047	7.4 ± 0.02	0.0001 ± 0.0004	9.9 ± 0.05

The obtained p*K*_*i*_ values show that the introduction of an indole group at the P2 position, as seen in **Neq1276**, results in an increase in binding affinity for hCatL (p*K*_*i*_ = 9.9). In contrast, the binding affinity of **Neq1276** for Cz remains significantly lower (p*K*_*i*_ = 7.4).

Root‐mean‐square deviation (RMSD) analysis was performed to evaluate the global structural stability of the enzyme–ligand complexes during the 100 ns MD simulations (Figures S6 and S7). In general, all systems reached conformational equilibration during the initial stage of the simulations and remained stable throughout the trajectories, without significant structural deviations, indicating that the covalent complexes remained structurally preserved over time.

RMSD analysis was performed to evaluate the global structural stability of the enzyme–ligand complexes during the 100 ns MD simulations (Figures S6 and S7). In general, all systems reached conformational equilibration during the initial stage of the simulations and remained stable throughout the trajectories, without significant structural deviations.

For the hCatL complexes, the RMSD profiles remained relatively stable after the equilibration phase, with average oscillations compatible with the maintenance of the overall tertiary structure of the enzyme. Although small fluctuations were observed during the simulations, these variations are expected for flexible solvent‐exposed regions and do not indicate relevant conformational rearrangements. Among the analyzed systems, complexes containing **Neq1181.1** and **Neq1182.1** exhibited lower RMSD oscillations, suggesting that these ligands promote a more stable conformational profile within the catalytic site. In contrast, **Neq1171.1** and **Neq1268** displayed slightly larger fluctuations along the trajectories, indicating increased local flexibility and dynamic adaptation of the binding pocket.

Similarly, the Cz complexes exhibited stable RMSD behavior during the simulations, confirming the structural stability of the enzyme and the maintenance of the ligand binding mode throughout the trajectories. After the initial equilibration period, all systems converged to stable RMSD values, with no evidence of progressive structural drift. The complexes containing **Neq1181.1** and **Neq1182.1** again presented lower RMSD variation compared to the remaining systems, which is consistent with the more persistent hydrophobic (HY) and aromatic interactions established within the S2 subsite. In particular, the presence of the phenyl group at the P2 position appears to favor a more rigid interaction network involving residues such as Trp26 and Phe28, contributing to the reduction of conformational mobility during the simulations. Conversely, systems containing **Neq1172.1** and **Neq1171.1** exhibited slightly broader RMSD fluctuations.

Root Mean Square Fluctuation (RMSF) analysis was conducted to evaluate the local conformational flexibility of the protein residues upon ligand binding. For the hCatL complexes, the RMSF profiles remained largely stable, with residual fluctuations typically ranging between 1.0 and 2.5 Å. A notable exception was observed in the third loop region (residues 172–180), which comprises a predominantly polar and charged segment (including Glu, Asp, Lys, and Ser). In the complex with **Neq1171.1**, this region reached a peak fluctuation of 3.8 Å, whereas other systems maintained values below 3.0 Å (Figure S8). Furthermore, a highly flexible region was identified between residues 42 and 50, characterized by an amphipathic sequence of residues (Thr, Gly, Arg, Leu, Ile, Ser, Leu, Ser, and Glu). The terminal segments also exhibited pronounced conformational mobility throughout the simulations, a behavior typical of solvent‐exposed disordered regions.

In the Cz complexes, five distinct loop regions displayed higher relative flexibility compared to the core secondary structure elements, though overall fluctuations remained within a low range of 1.0–2.5 Å (Figure S9). Specifically, Loop 1 showed a significant reduction in mobility when bound to **Neq1181.1**, whereas complexes with **Neq1171.1** and **Neq1276** exhibited higher fluctuations, approximately 2.5 Å. The presence of aromatic moieties, such as the benzene and indole rings in **Neq1181.1** and **Neq1268**, appears to enhance the stabilization of Loop 5, a component of the S2 subsite, compared to ligands featuring a Leu residue at the P2 position. Despite these local variations, the overall RMSF profiles suggest a conserved binding mode across the series. Notably, Loops 3 and 5, which contribute to the S1 and S2 pockets, respectively, demonstrated high structural conservation with fluctuations not exceeding 1.5 Å across all Cz complexes, underscoring their role in maintaining proper ligand alignment within the catalytic site.

### 3.2 Fingerprint Analysis

The interactions between the ligands and the residues of the catalytic site of hCatL were also evaluated through fingerprint analysis using the ProLIF program, revealing a consistent interaction pattern across the analyzed inhibitors. The main residues involved in ligand recognition in all systems include Gln19, Gly23, Gly68, Asp162, Leu69, Met161, Met70, Ser24, Ser29, and Trp26, with occurrence values frequently higher than 0.5, indicating stable and persistent contacts throughout the simulations. Among the analyzed systems, ligands such as **Neq1172.1** and **Neq1181.1** presented a higher number of interactions with elevated occurrence, which is directly associated with a more stable conformational profile during the MD simulations. In contrast, **Neq1268** and **Neq1171.1** showed a more heterogeneous interaction distribution, with reduced occupancy in key regions, suggesting a less optimized binding mode (Figure 4).

**FIGURE 4:**
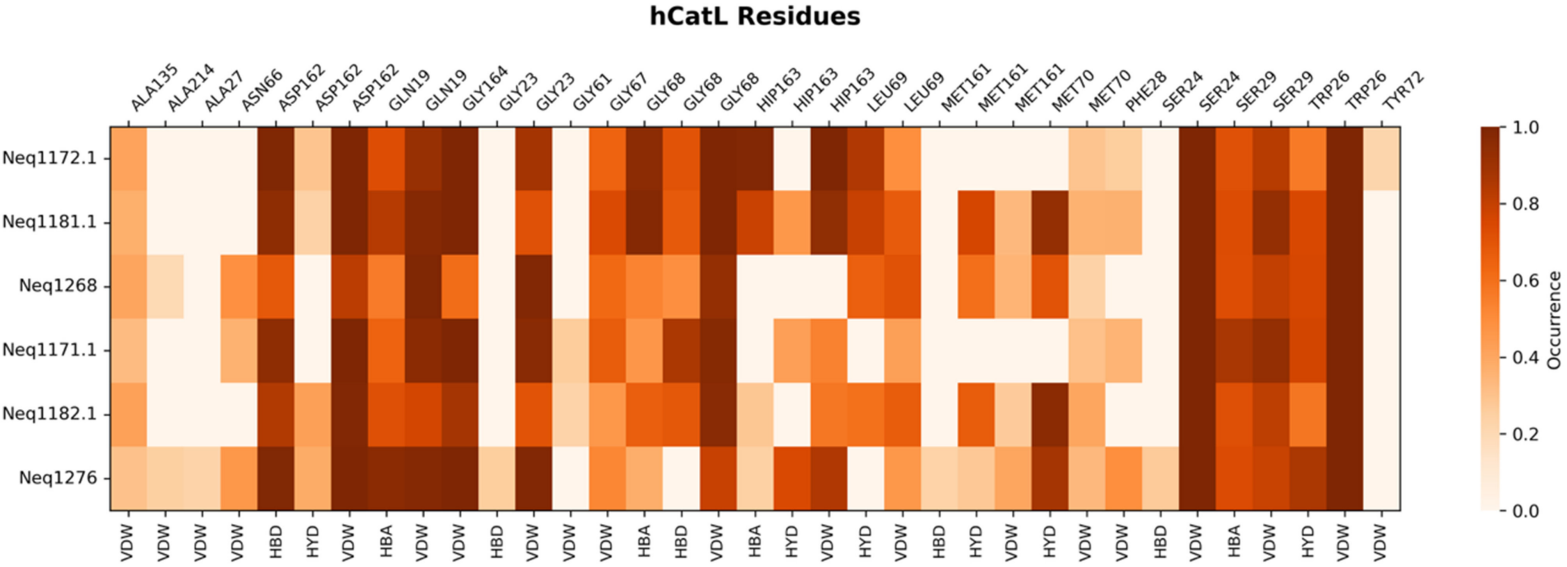
Interaction fingerprints (IFPs) of hCatL complexes, including interaction type and occurrence values, classified as hydrophobic (HY), van der Waals (vdW), hydrogen bond donor (HBD), and hydrogen bond acceptor (HBA) interactions for the Neq ligand series.

The hydrogen bond (H‐bond) analysis for the hCatL complexes reveals a subset of highly persistent interactions with occupancies exceeding 50% and mean distances consistently below 2.90 Å, indicating strong and stable electrostatic stabilization. Among the series, **Neq1172.1** exhibits a H‐bond profile anchored by a primary interaction between the ligand and His163 (91.73% occupancy, 2.78 Å) and a secondary contact with the carbonyl oxygen of Asp162 (69.32%, 2.87 Å). **Neq1181.1** demonstrates a distinct stabilization pattern, characterized by significant H‐bonds with the backbone of Gly68 (64.46%, 2.88 Å) and the sidechain of His163 (59.56%, 2.82 Å).

The role of Asp162 (Figure 4) as a key hydrogen bond acceptor (HBA) is further evidenced in the complexes with **Neq1268** and **Neq1171.1**, which maintain occupancies of 55.01% (2.83 Å) and 59.69% (2.88 Å), respectively. Furthermore, **Neq1182.1** and **Neq1276** are effectively anchored by Gln19, displaying occupancies of 56.14% (2.87 Å) and 67.92% (2.89 Å). **Neq1276** additionally maintains a high‐occupancy interaction with Asp162 (67.51%, 2.87 Å), suggesting a dual‐anchoring mechanism that likely contributes to its conformational stability within the catalytic site. Overall, these interactions are characterized by short donor–acceptor distances, reinforcing their role as critical determinants of ligand recognition and binding persistence in hCatL.

Moreover, residues Trp26 and Phe28 are involved in π‐type and HY interactions with the aromatic moieties of the ligands, contributing significantly to binding stabilization. The interaction profile also highlights the importance of polar residues such as Asp162 and Gln19, which frequently participate in hydrogen bond donor (HBD) and acceptor interactions, playing a central role in ligand anchoring within the binding site. In addition, residues Ser24 and Ser29 contribute to hydrogen bonding networks, although with more variable occurrence depending on the ligand (Figure 4). The complexes are predominantly stabilized by vdW and HY interactions, especially within regions formed by Leu69, Met161, and Met70, which constitute a well‐defined HY pocket. These interactions are complemented by hydrogen bonds that reinforce ligand positioning and orientation (Figure 4).

In all simulated systems, the covalent ligands consistently showed high occupancy (above 0.5) in interactions with the Cz catalytic site. Key residues involved in these interactions include Asp161, Gln19, Gly23, Gly65, Gly66, Gly163, His162, Ser24, Ser29, and Trp26. This signifies the presence of highly stable and persistent contacts over the course of the trajectories. The increased occupancy observed for **Neq1181.1** and **Neq1182.1** is primarily driven by the structural complementarity and HY burial provided by their P2 phenyl substituent within the S2 subsite; the interaction with Phe28 and Trp184 is stabilized by aromatic π‐stacking (displaced or T‐shaped) and π‐CH interactions, where the electron‐rich systems of the ligand’s benzene ring and the residues’ side chains overlap to provide significant dispersion‐driven stabilization. Furthermore, the contact with Glu208 contributes to binding through HY effects involving its methylene groups, which effectively wrap around the ligand’s P2 ring. This combination of HY complementarity and π‐network stabilization creates a more rigid and persistent binding profile for the benzenic series. This enhanced interaction network appears to contribute to the structural stability during the MD simulations. Conversely, compounds such as **Neq1171.1** and **Neq1172.1** displayed a more scattered interaction profile, lacking high occupancy in these supplementary regions and depending on localized contacts like Ser64, which points to a less favorable binding conformation.

Although MD simulations indicate that the benzenic series (**Neq1182**) establishes a highly rigid interaction network within the S2 pocket, the experimental data reveals a strong preference for the aliphatic leucine substitution; the rigid π‐stacking interactions observed for the benzyl group likely incur a severe conformational entropy penalty and imperfect solvent displacement. Conversely, the isobutyl group of leucine provides shape complementarity and minimizes steric strain, exploiting the classical HY effect to achieve superior overall thermodynamic affinity in Cz.

Furthermore, an analysis of the interaction types underscores the critical function of polar residues within the binding pocket. Asp161 operates predominantly as a HBD, whereas Gln19 and Ser29 function as reliable HBAs, collectively serving as primary anchor points for the ligands. These polar contacts are further reinforced by hydrogen bonding networks involving the backbones or sidechains of Gly23 and Gly66, which show variable occurrence depending on the ligand but contribute significantly to binding stabilization when present. Residue Gln19 acts as a highly persistent HBA for the inhibitor’s backbone amides in both systems. Furthermore, residues Asp161 (in cruzain) and Asp162 (in hCatL) form essential hydrogen bonds that stabilize the positioning of the nitrile warhead and the nitrogen of the thioimidate adduct. Quantitative occupancy analysis reveals that potent inhibitors, such as **Neq1182.1** in hCatL, maintain these specific interactions with mean distances below 2.90 Å for more than 60% of the simulation time.

Similarly to hCatL, the Cz complexes are predominantly stabilized by vdW and HY interactions, especially within regions formed by Leu67, Met68, Leu160, and His162, which constitute a well‐defined HY pocket (Figure 5). These noncovalent interactions are complemented by extensive and persistent vdW contacts from Trp26, Phe28, and glycine‐rich segments that reinforce ligand positioning and orientation. Overall, the results indicate that ligands exhibiting a greater number of interactions with high occurrence values, particularly those combining strong contacts within the HY pocket with consistent hydrogen bonding, tend to display more stable behavior.

**FIGURE 5:**
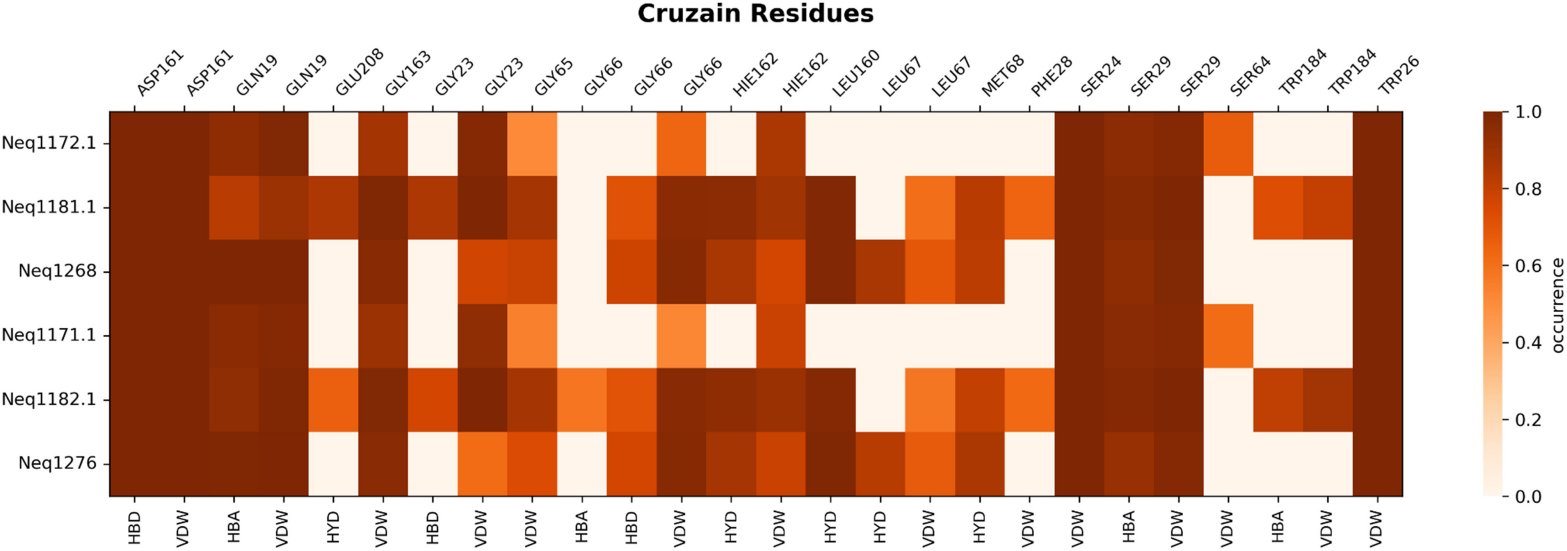
Interaction fingerprints (IFP) of cruzain complexes, including interaction type and occurrence values, classified as hydrophobic (HY), van der Waals (vdW), hydrogen bond donor (HBD), and hydrogen bond acceptor (HBA) interactions for the Neq ligand series.

The H‐bond analysis for the Cz complexes demonstrates a highly conserved and persistent interaction network (Figure 5), with several key contacts exceeding 50% occupancy and maintaining mean distances between 2.80 and 2.89 Å. The residue Asp161 acts as a primary anchor across the covalent inhibitors, forming an H‐bond with the nitrogen atoms of ligands with occupancies ranging from 85.01% to 95.33% and mean distances consistently around 2.81–2.84 Å. Another key interaction is observed with Gln19. This contact occurs in the complexes with **Neq1268** (94.14%, 2.83 Å) and **Neq1276** (93.26%, 2.83 Å), though it remains significant for **Neq1172.1** (79.41%) and Neq1171.1 (64.52%). Additionally, Ser29 contributes to the stabilization of several ligands through its backbone nitrogen, showing high‐occupancy H‐bonds with Neq1182.1 (73.43%, 2.87 Å), Neq1181.1 (71.00%, 2.87 Å), and **Neq1268** (56.90%, 2.88 Å). Notably, **Neq1181.1** is further stabilized by an interaction with the carbonyl oxygen of Gly23 (58.19%, 2.84 Å), a contact that is less prevalent or falls below the 50% threshold in other systems.

Interestingly, the S2 sub‐pocket differs markedly between the two enzymes, thereby governing ligand selectivity. In hCatL, the residue corresponding to Cz Glu205 is an Ala214 (Figure 6), resulting in a more HY S2 pocket. Consequently, the introduction of an indole group at the P2 position, as observed for **Neq1276**, promotes favorable HY packing and enhances binding affinity toward hCatL. In contrast, Cz possesses a Glu205 residue within the S2 pocket. This structural difference allows Cz to accommodate more polar substituents at the P2 position. Therefore, high potency against Cz generally requires substituents capable of effectively interacting with Glu205. This may explain why **Neq1276** displays significantly lower affinity for Cz (p*K*_*i*_ = 7.4) despite its enhanced potency toward hCatL.

**FIGURE 6:**
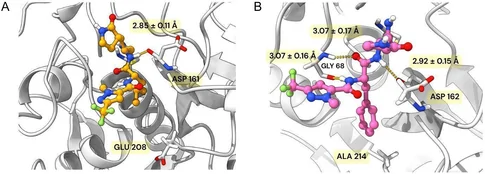
Detailed view of the binding modes of the covalent ligands within the catalytic sites of cruzain and hCatL. (A) Neq1172.1 complexed with cruzain, highlighting the stable hydrogen bond formed with residue Asp161. The S2 subsite is characterized by the presence of the Glu208 residue. (B) Neq1181.1 within the active site of hCatL, illustrating the dual interaction pattern of Gly68, which acts as both a hydrogen bond donor and acceptor. The corresponding position in the S2 subsite is occupied by the Ala214 residue. Hydrogen bonds are represented by dashed lines; key residues and the ligand are shown in stick representation.

### 3.3 Relative Binding Free Energy

A major objective of this work was to assess whether computational binding free energy calculations are capable of reproducing the experimentally observed affinities of reversible covalent inhibitors by modeling only the covalent state of the protein–ligand complex. In this context, alchemical free energy calculations based on TI were employed to investigate whether this approach could reliably capture the differences in binding affinity among the nitrile‐based inhibitors **Neq1171.1**, **Neq1172.1**, **Neq1181.1**, **Neq1182.1**, **Neq1268**, and **Neq1276** toward Cz and hCatL, as determined by enzymatic kinetic assays. For the **Neq1172.1** → **Neq1171.1** transformation, an excellent agreement is observed for both targets, with minimal deviations between calculated and experimental values. The calculated ΔΔ*G* of −1.04 ± 0.02 kcal/mol (Table 3) for the Cz complex reproduces the experimental affinity difference of −1.10 kcal/mol between ligands **Neq1171.1** (p*K*_*i*_ = 7.9; Δ*G*_exp_ = −10.84 kcal/mol) and **Neq1172.1** (p*K*_*i*_ = 7.1; Δ*G*_exp_ = −9.75 kcal/mol), while for hCatL, the calculated ΔΔ*G* of −0.98 ± 0.10 kcal/mol (Table 3) closely matches the experimental shift of −0.96 kcal/mol (pK_*i*_ = 8.0; Δ*G*_exp_ = −10.96 kcal/mol vs. p*K*_*i*_ = 7.3; Δ*G*_exp_ = −10.00 kcal/mol).

**TABLE 3:** Experimental and predicted relative binding free energies (ΔΔ*G*) for the Neq inhibitor series in complex with cruzain and human cathepsin L (hCatL). Values represent the free energy shift (kcal mol^−1^) associated with specific structural modifications. Calculated values include standard error from triplicate trajectories.

	Cruzain	hCatL
ΔΔ*G*_exp_, kcal mol^−1^		
Neq1172.1 → Neq1171.1	−1.1	−0.96
Neq1181.1 → Neq1182.1	−1.1	−1.65
Neq1268 → Neq1276	−0.69	−1.37
ΔΔ*G*_calc_, kcal mol^−1^		
Neq1172.1 → Neq1171.1	−1.04 ± 0.02	−0.98 ± 0.10
Neq1181.1 → Neq1182.1	−0.81 ± 0.09	−1.15 ± 0.18
Neq1268 → Neq1276	−0.76 ± 0.27	−1.83 ± 0.28

In the case of **Neq1181.1** → **Neq1182.1**, the calculated values slightly underestimate the experimental gains in binding affinity, particularly for hCatL. For this pair, the Cz simulation yields a ΔΔ*G* of −0.81 ± 0.09 kcal/mol (Table 3) against an experimental difference of −1.10 kcal/mol (p*K*_*i*_ = 7.3; Δ*G*_exp_ = −10.00 kcal/mol vs. p*K*_*i*_ = 6.5; Δ*G*_exp_ = −8.91 kcal/mol), whereas the hCatL calculation yields a ΔΔ*G* of −1.15 ± 0.18 kcal/mol, underestimating the more pronounced experimental gain of −1.65 kcal/mol (p*K*_*i*_ = 8.5; Δ*G*_exp_ = −11.65 kcal/mol vs. p*K*_*i*_ = 7.3; Δ*G*_exp_ = −10.00 kcal/mol). Although the favorable character of the transformation is correctly reproduced, the observed deviations may be associated with more complex structural or solvation effects, which are known to be more challenging to fully capture in free energy calculations. For the **Neq1268** → **Neq1276** transformation, the ΔΔ*G* correspond to −0.76 ± 0.27 kcal/mol compared to the experimental −0.69 kcal/mol in Cz (p*K*_*i*_ = 7.4; Δ*G*_exp_ = −10.14 kcal/mol vs. p*K*_*i*_ = 6.9; Δ*G*_exp_ = −9.45 kcal/mol), whereas in hCatL calculation yields a ΔΔ*G* of −1.83 ± 0.28 kcal/mol, overestimating the experimental difference of −1.37 kcal/mol (p*K*_*i*_ = 9.9; Δ*G*_exp_ = −13.56 kcal/mol vs. p*K*_*i*_ = 8.9; Δ*G*_exp_ = −12.19 kcal/mol). Experimental binding free energy was estimated using the following equation:

(4) $$\text{Δ}G_{\mathrm{bind},\exp}=-RT\ln\left(\frac{1}{K_{i}}\right)$$

Experimental relative binding free energies (ΔΔ*G*) for the Neq inhibitor series in complex with Cz and hCatL were obtained by calculating the difference in the corresponding experimental binding free energies $\text{Δ}G_{\mathrm{bind},\exp}$ of ligands (Table 3).

The replacement of the P3 trifluoromethyl group with a tert‐butyl moiety increases binding affinity through multiple thermodynamic and structural factors. While C─F bonds are polarized and form dipole–dipole interactions with water, the tert‐butyl group is nonpolar and HY. Consequently, the desolvation penalty for the tert‐butyl group is lower, and its burial in the HY S3 subpocket is driven by an increase in solvent entropy upon the release of structured water molecules. Structurally, the tert‐butyl group optimizes vdW contacts with residues such as Gly61 in hCatL and Gly66 in Cz, leading to enhanced complex stabilization. Furthermore, the high electron density of fluorine atoms in the trifluoromethyl group induces electrostatic repulsion from the lone pairs of backbone carbonyl oxygens, such as those in Gly61, whereas the methyl groups in the tert‐butyl moiety avoid this repulsion and engage in CH–π interactions with proximal aromatic residues.

Despite these deviations, the calculated results consistently reproduce the correct direction of the binding free energy changes across all transformations. This ability to capture relative trends is particularly relevant in the context of ligand optimization. In addition, the differences between Cz and hCatL are qualitatively preserved, indicating that the method is able to describe the distinct interaction patterns within each binding site. The reliability of these relative trends is supported by robust sampling statistics across all transformations for both enzymes, with internal TI errors remaining below 0.14 kcal/mol and final forward–backward hysteresis values predominantly lower than 0.05 kcal/mol. Detailed TI curves, block averages, forward–backward convergence profiles, and the convergence of each lambda window per replica confirming this statistical stability are provided in the Supporting Information (Figures S10–S51). Overall, the results support the use of TI as a reliable approach for estimating relative binding free energies in this system, providing useful insights to guide the rational design and optimization of inhibitors.

## 4 Conclusions

Here, we integrated experimental enzyme kinetics with MD simulations and relative binding free energy calculations to evaluate a series of nitrile‐based reversible covalent inhibitors of hCatL and Cz, namely, **Neq1171.1**, **Neq1172.1**, **Neq1181.1**, **Neq1182.1**, **Neq1268**, **and Neq1276**. MD simulations further confirm that all covalent enzyme‐ligand complexes remain structurally stable over three replicas of 100 ns trajectories for each system. IFP analysis reveals a conserved network of key residues governing binding in both enzymes. In hCatL, persistent hydrogen bonding interactions with residues such as Gln19, Gly68, and Asp162, together with HY and π‐type contacts involving Trp26 and Phe28, are central to ligand stabilization. In Cz, a similarly stable interaction network is observed, dominated by Asp161 as a primary anchoring residue and reinforced by contributions from Gln19, Ser29, and surrounding HY pocket residues. Importantly, the S2 sub‐pocket composition emerges as a key determinant of selectivity. In hCatL, the position equivalent to Cz Glu205 is occupied by Ala, resulting in a more HY pocket that favors **Neq1276‐**hCatL interactions. In contrast, Cz contains Glu205, which allows polar substituents and reduces optimal complementarity for purely HY P2 groups, explaining the reduced affinity of **Neq1276** toward Cz. We also show that the relative binding affinities estimated using alchemical transformation simulation and TI are in agreement with the experimental data. The results reported here may be used for rational design and optimization of cysteine protease inhibitors for applications in Chagas disease and anticancer drug discovery.

## Funding

This work was supported by the Conselho Nacional de Desenvolvimento Científico e Tecnológico; 305182/2021‐8, 402311/2024‐8 and 404652/2025‐5.

## Conflicts of Interest

The authors declare no conflicts of interest.

## Supporting information

Supplementary Material

## Data Availability

The data that support the findings of this study are available from the corresponding author upon reasonable request.
