# Supplementary material for: Assessment of the Binding Affinity of Covalent Inhibitors of Cruzain and Cathepsin L Using Relative Binding Free Energy
Source: ChemMedChem. 2026 Sep 27;21(19):e70508. doi: 10.1002/cmdc.70508 (PMC13616992; doi:10.1002/cmdc.70508)
Supplement: Supplementary file 1 [file CMDC-21-e70508-s001.pdf]

Support Information:

Predicting the Binding Affinity of Covalent Inhibitors of cruzain and cathepsin L Using Relative Binding Free Energy

Yan Carvalho Magno[1], Luiz Fernando Barbosa[2], Maycon Vinicius Damasceno de Oliveira[1], Jerônimo Lameira[1*] Carlos A. Montanari[2*]

[1] *Laboratório de Planejamento e Desenvolvimento de Fármacos, Instituto de Ciências Exatas e Naturais, Universidade Federal do Pará, 66075-110, Belém, Pará, Brasil.*
[2]*Medicinal and Biological Chemistry Group, São Carlos Institute of Chemistry, University of São Paulo. Avenida Trabalhador Sancarlense, 400, 13566-590, São Carlos, SP, Brazil.*

**Corresponding Authors: JL, lameira@ufpa.br; CAM, carlos.montanari@usp.br*

Yan Carvalho Magno: 0009-0000-7419-1864.
Maycon Vinicius Damasceno de Oliveira: 0000-0003-2263-2124.
Jerônimo Lameira: 0000-0001-7270-1517.
Luiz Fernando Barbosa: 0000-0002-3212-4513.
Carlos A. Montanari: 0000-0002-4963-0316

CONTENTS

Chemistry

Figure S1. Synthetic route to Neq1181. (a) - NH3 in MeOH (17 eq.), r.t, 48 h; (b) – DIPEA (3 eq.), THF dry, TFAA (1,3 eq.), 0°C → r.t., 2 h; (c) – HCl 4 M in Dioxane (3 eq.), ACN dry, 0 °C → r.t, 2 h; (d) – HATU (1,2 eq.), DIPEA (2,6 eq.), DMF dry, r.t, 20 h; (e) – LiOH (2 M), THF dry, r.t, 2 h;

Figure S2. 1H NMR Spectrum of Neq1181.

Figure S3. 13C NMR Spectrum of Neq1181.

Figure S4. HPLC chromatogram of Neq1181.

Biochemical assays

Figure S5. Biochemical assays of compounds against Cz and hCatL[1].

Figure S6. Root-mean-square deviation (RMSD) plots over 100 ns of simulation for the hCatL enzyme complexes with the Neq ligand series (Neq1172.1, Neq1181.1, Neq1268, Neq1171.1, Neq1182.1, Neq1276) in triplicate.

Figure S7. Root-mean-square deviation (RMSD) plots over 100 ns of simulation for the cruzain enzyme complexes with the Neq ligand series (Neq1172.1, Neq1181.1, Neq1268, Neq1171.1, Neq1182.1, Neq1276) in triplicate.

Figure S8. Root-mean-square fluctuation (RMSF) analysis per residue for the hCatL complexes, with a three-dimensional structural projection highlighting the regions of greatest flexibility (Loops 1, 2, and 3).

Figure S9. Root-mean-square fluctuation (RMSF) analysis per residue for the cruzain complexes, with a three-dimensional structural projection highlighting the regions of greatest flexibility (Loops 1 to 5).

Figure S10. Free energy convergence analysis for the alchemical transformation from cruzain-Neq1172.1 to cruzain-Neq1171.1. (a) Thermodynamic Integration (TI) curve showing the gradient (U/) as a function of the coupling parameter for both the complex and solvent phases. (b) Block average convergence analysis and (c) Forward-Backward bidirectional convergence estimate of the free energy variation (ΔG) for the complex phase. (d) Block average convergence analysis and (e) Forward-Backward bidirectional convergence estimate of ΔG for the solvent leg.

Figure S11. Temporal convergence profile of ⟨∂U/∂λ⟩ across all simulated λ windows in the complex step for the alchemical transformation from cruzain-Neq1172.1 to cruzain-Neq1171.1, (replicate 1).

Figure S12. Temporal convergence profile of ⟨∂U/∂λ⟩ across all simulated λ windows in the complex step for the alchemical transformation from cruzain-Neq1172.1 to cruzain-Neq1171.1, (replicate 2).

Figure S13. Temporal convergence profile of ⟨∂U/∂λ⟩ across all simulated λ windows in the complex step for the alchemical transformation from cruzain-Neq1172.1 to cruzain-Neq1171.1, (replicate 3).

Figure S14. Temporal convergence profile of ⟨∂U/∂λ⟩ across all simulated λ windows in the solvent step for the alchemical transformation from cruzain-Neq1172.1 to cruzain-Neq1171.1, (replicate 1).

Figure S15. Temporal convergence profile of ⟨∂U/∂λ⟩ across all simulated λ windows in

the solvent step for the alchemical transformation from cruzain-Neq1172.1 to cruzain-Neq1171.1, (replicate 2).

Figure S16. Temporal convergence profile of $\langle \partial U/\partial \lambda \rangle$ across all simulated λ windows in the solvent step for the alchemical transformation from cruzain-Neq1172.1 to cruzain-Neq1171.1, (replicate 3).

Figure S17. Free energy convergence analysis for the alchemical transformation from cruzain-Neq1181.1 to cruzain-Neq1182.1. (a) Thermodynamic Integration (TI) curve showing the gradient (U/) as a function of the coupling parameter for both the complex and solvent phases. (b) Block average convergence analysis and (c) Forward-Backward bidirectional convergence estimate of the free energy variation (ΔG) for the complex phase. (d) Block average convergence analysis and (e) Forward-Backward bidirectional convergence estimate of ΔG for the solvent leg.

Figure S18. Temporal convergence profile of $\langle \partial U/\partial \lambda \rangle$ across all simulated λ windows in the complex step for the alchemical transformation from cruzain-Neq1181.1 to cruzain-Neq1182.1, (replicate 1).

Figure S19. Temporal convergence profile of $\langle \partial U/\partial \lambda \rangle$ across all simulated λ windows in the complex step for the alchemical transformation from cruzain-Neq1181.1 to cruzain-Neq1182.1, (replicate 2).

Figure S20. Temporal convergence profile of $\langle \partial U/\partial \lambda \rangle$ across all simulated λ windows in the complex step for the alchemical transformation from cruzain-Neq1181.1 to cruzain-Neq1182.1, (replicate 3).

Figure S21. Temporal convergence profile of $\langle \partial U/\partial \lambda \rangle$ across all simulated λ windows in the solvent step for the alchemical transformation from cruzain-Neq1181.1 to cruzain-Neq1182.1, (replicate 1).

Figure S22. Temporal convergence profile of $\langle \partial U/\partial \lambda \rangle$ across all simulated λ windows in the solvent step for the alchemical transformation from cruzain-Neq1181.1 to cruzain-Neq1182.1, (replicate 2).

Figure S23. Temporal convergence profile of $\langle \partial U/\partial \lambda \rangle$ across all simulated λ windows in the solvent step for the alchemical transformation from cruzain-Neq1181.1 to cruzain-Neq1182.1, (replicate 3).

Figure S24. Free energy convergence analysis for the alchemical transformation from cruzain-Neq1268 to cruzain-Neq1276. (a) Thermodynamic Integration (TI) curve showing the gradient (U/) as a function of the coupling parameter for both the complex and solvent phases. (b) Block average convergence analysis and (c) Forward-Backward bidirectional convergence estimate of the free energy variation (ΔG) for the complex phase. (d) Block average convergence analysis and (e) Forward-Backward bidirectional convergence estimate of ΔG for the solvent leg.

Figure S25. Temporal convergence profile of $\langle \partial U/\partial \lambda \rangle$ across all simulated λ windows in the complex step for the alchemical transformation from cruzain-Neq1268 to cruzain-Neq1276, (replicate 1).

Figure S26. Temporal convergence profile of $\langle \partial U/\partial \lambda \rangle$ across all simulated λ windows in the complex step for the alchemical transformation from cruzain-Neq1268 to cruzain-Neq1276, (replicate 2).

Figure S27. Temporal convergence profile of $\langle \partial U/\partial \lambda \rangle$ across all simulated λ windows in the complex step for the alchemical transformation from cruzain-Neq1268 to cruzain-Neq1276, (replicate 3).

Figure S28. Temporal convergence profile of $\langle \partial U/\partial \lambda \rangle$ across all simulated λ windows in the solvent step for the alchemical transformation from cruzain-Neq1268 to cruzain-Neq1276, (replicate 1).

Figure S29. Temporal convergence profile of $\langle \partial U/\partial \lambda \rangle$ across all simulated λ windows in the solvent step for the alchemical transformation from cruzain-Neq1268 to cruzain-Neq1276, (replicate 2).

Figure S30. Temporal convergence profile of $\langle \partial U/\partial \lambda \rangle$ across all simulated λ windows in the solvent step for the alchemical transformation from cruzain-Neq1268 to cruzain-Neq1276, (replicate 3).

Figure S31. Free energy convergence analysis for the alchemical transformation from hCatL-Neq1172.1 to hCatL-Neq1171.1. (a) Thermodynamic Integration (TI) curve showing the gradient (U/) as a function of the coupling parameter for both the complex and solvent phases. (b) Block average convergence analysis and (c) Forward-Backward bidirectional convergence estimate of the free energy variation (ΔG) for the complex phase. (d) Block average convergence analysis and (e) Forward-Backward bidirectional convergence estimate of ΔG for the solvent leg.

Figure S32. Temporal convergence profile of $\langle \partial U/\partial \lambda \rangle$ across all simulated λ windows in the complex step for the alchemical transformation from hCatL-Neq1172.1 to hCatL-Neq1171.1, (replicate 1).

Figure S33. Temporal convergence profile of $\langle \partial U/\partial \lambda \rangle$ across all simulated λ windows in the complex step for the alchemical transformation from hCatL-Neq1172.1 to hCatL-Neq1171.1, (replicate 2).

Figure S34. Temporal convergence profile of $\langle \partial U/\partial \lambda \rangle$ across all simulated λ windows in the complex step for the alchemical transformation from hCatL-Neq1172.1 to hCatL-Neq1171.1, (replicate 3).

Figure S35. Temporal convergence profile of $\langle \partial U/\partial \lambda \rangle$ across all simulated λ windows in the solvent step for the alchemical transformation from hCatL-Neq1172.1 to hCatL-Neq1171.1, (replicate 1).

Figure S36. Temporal convergence profile of $\langle \partial U/\partial \lambda \rangle$ across all simulated λ windows in the solvent step for the alchemical transformation from hCatL-Neq1172.1 to hCatL-Neq1171.1, (replicate 2).

Figure S37. Temporal convergence profile of $\langle \partial U/\partial \lambda \rangle$ across all simulated λ windows in the solvent step for the alchemical transformation from hCatL-Neq1172.1 to hCatL-Neq1171.1, (replicate 3).

Figure S38. Free energy convergence analysis for the alchemical transformation from hCatL-Neq1181.1 to hCatL-Neq1182.1. (a) Thermodynamic Integration (TI) curve showing the gradient (U/) as a function of the coupling parameter for both the complex and solvent phases. (b) Block average convergence analysis and (c) Forward-Backward bidirectional convergence estimate of the free energy variation (ΔG) for the complex phase. (d) Block

average convergence analysis and (e) Forward-Backward bidirectional convergence estimate of ΔG for the solvent leg.

Figure S39. Temporal convergence profile of $\langle \partial U/\partial \lambda \rangle$ across all simulated λ windows in the complex step for the alchemical transformation from hCatL-Neq1181.1 to hCatL-Neq1182.1, (replicate 1).

Figure S40. Temporal convergence profile of $\langle \partial U/\partial \lambda \rangle$ across all simulated λ windows in the complex step for the alchemical transformation from hCatL-Neq1181.1 to hCatL-Neq1182.1, (replicate 2).

Figure S41. Temporal convergence profile of $\langle \partial U/\partial \lambda \rangle$ across all simulated λ windows in the complex step for the alchemical transformation from hCatL-Neq1181.1 to hCatL-Neq1182.1, (replicate 3).

Figure S42. Temporal convergence profile of $\langle \partial U/\partial \lambda \rangle$ across all simulated λ windows in the solvent step for the alchemical transformation from hCatL-Neq1181.1 to hCatL-Neq1182.1, (replicate 1).

Figure S43. Temporal convergence profile of $\langle \partial U/\partial \lambda \rangle$ across all simulated λ windows in the solvent step for the alchemical transformation from hCatL-Neq1181.1 to hCatL-Neq1182.1, (replicate 2).

Figure S44. Temporal convergence profile of $\langle \partial U/\partial \lambda \rangle$ across all simulated λ windows in the solvent step for the alchemical transformation from hCatL-Neq1181.1 to hCatL-Neq1182.1, (replicate 3).

Figure S45. Free energy convergence analysis for the alchemical transformation from hCatL-Neq1268 to hCatL-Neq1276. (a) Thermodynamic Integration (TI) curve showing the gradient (U/) as a function of the coupling parameter for both the complex and solvent phases. (b) Block average convergence analysis and (c) Forward-Backward bidirectional convergence estimate of the free energy variation (ΔG) for the complex phase. (d) Block average convergence analysis and (e) Forward-Backward bidirectional convergence estimate of ΔG for the solvent leg.

Figure S46. Temporal convergence profile of $\langle \partial U/\partial \lambda \rangle$ across all simulated λ windows in the complex step for the alchemical transformation from hCatL-Neq1268 to hCatL-Neq1276, (replicate 1).

Figure S47. Temporal convergence profile of $\langle \partial U/\partial \lambda \rangle$ across all simulated λ windows in the complex step for the alchemical transformation from hCatL-Neq1268 to hCatL-Neq1276, (replicate 2).

Figure S48. Temporal convergence profile of $\langle \partial U/\partial \lambda \rangle$ across all simulated λ windows in the complex step for the alchemical transformation from hCatL-Neq1268 to hCatL-Neq1276, (replicate 3).

Figure S49. Temporal convergence profile of $\langle \partial U/\partial \lambda \rangle$ across all simulated λ windows in the solvent step for the alchemical transformation from hCatL-Neq1268 to hCatL-Neq1276, (replicate 1).

Figure S50. Temporal convergence profile of $\langle \partial U/\partial \lambda \rangle$ across all simulated λ windows in the solvent step for the alchemical transformation from hCatL-Neq1268 to hCatL-Neq1276, (replicate 2).

Figure S51. Temporal convergence profile of ⟨∂U/∂λ⟩ across all simulated λ windows in the solvent step for the alchemical transformation from hCatL-Neq1268 to hCatL-Neq1276, (replicate 3).

Chemistry

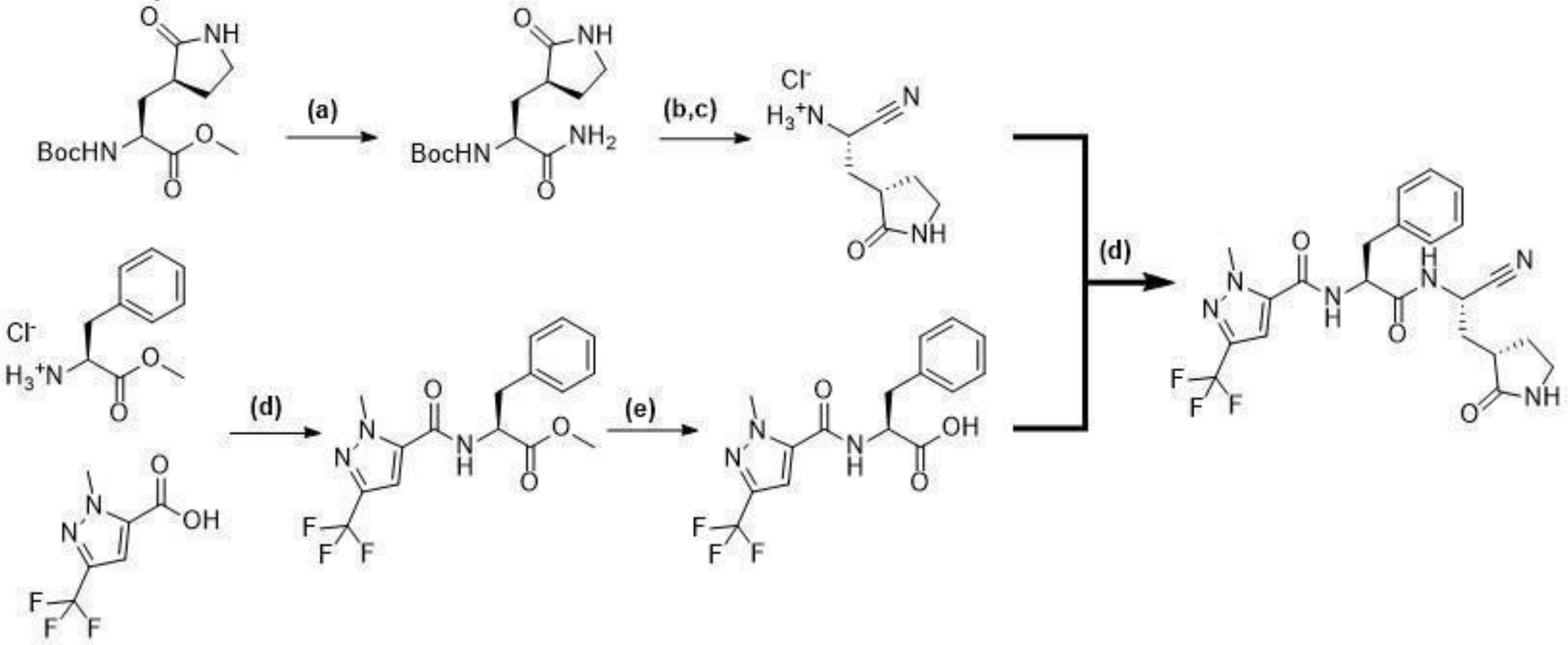

Figure S1. Synthetic route to Neq1181. (a) - NH_3 in MeOH (17 eq.), r.t, 48 h; (b) – DIPEA (3 eq.), THF dry, TFAA (1,3 eq.), 0°C → r.t., 2 h; (c) – HCl 4 M in Dioxane (3 eq.), ACN dry, 0 °C → r.t, 2 h; (d) – HATU (1,2 eq.), DIPEA (2,6 eq.), DMF dry, r.t, 20 h; (e) – LiOH (2 M), THF dry, r.t, 2 h;

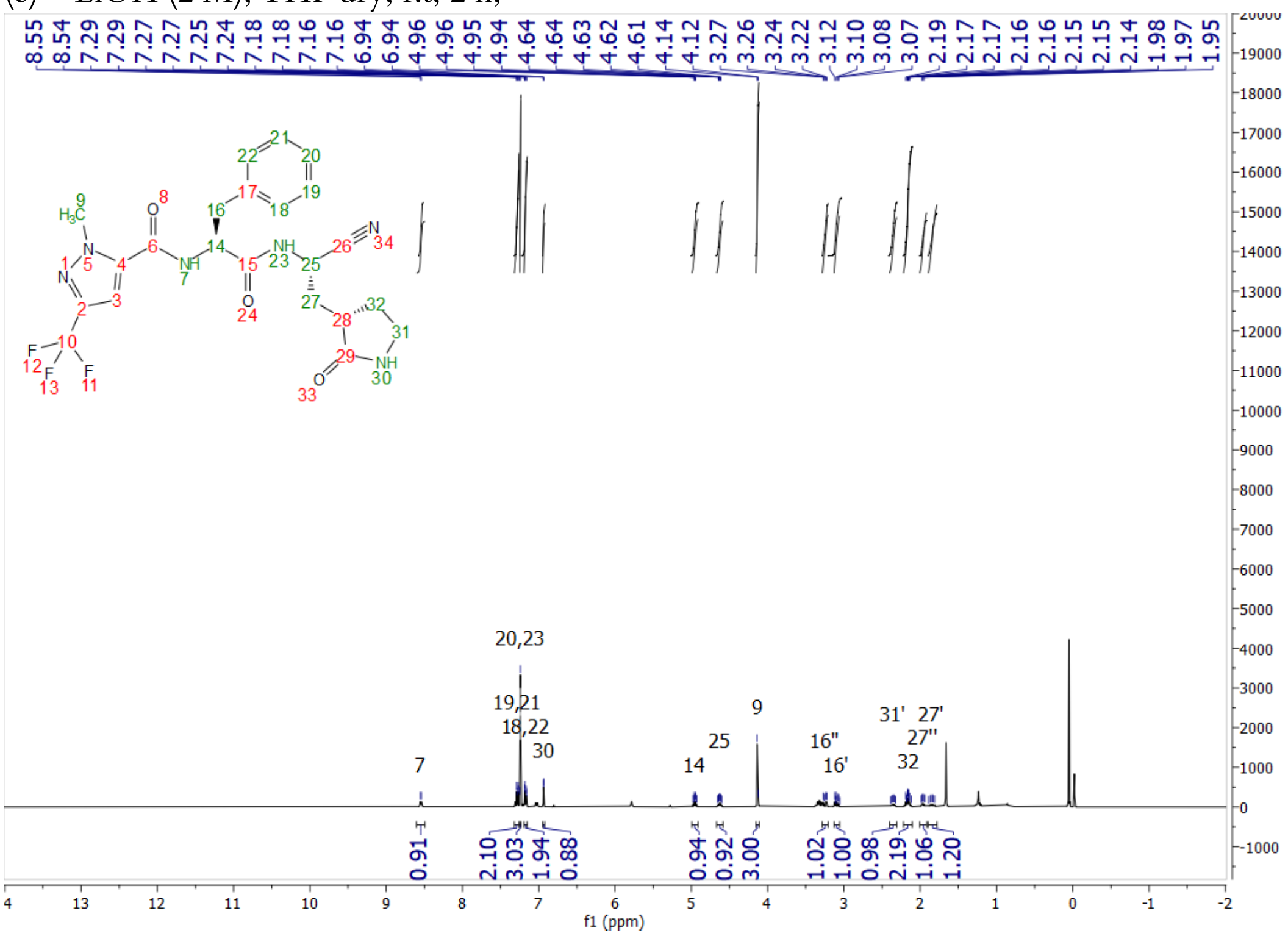

Figure S2. ^{1}H NMR Spectrum of Neq1181.

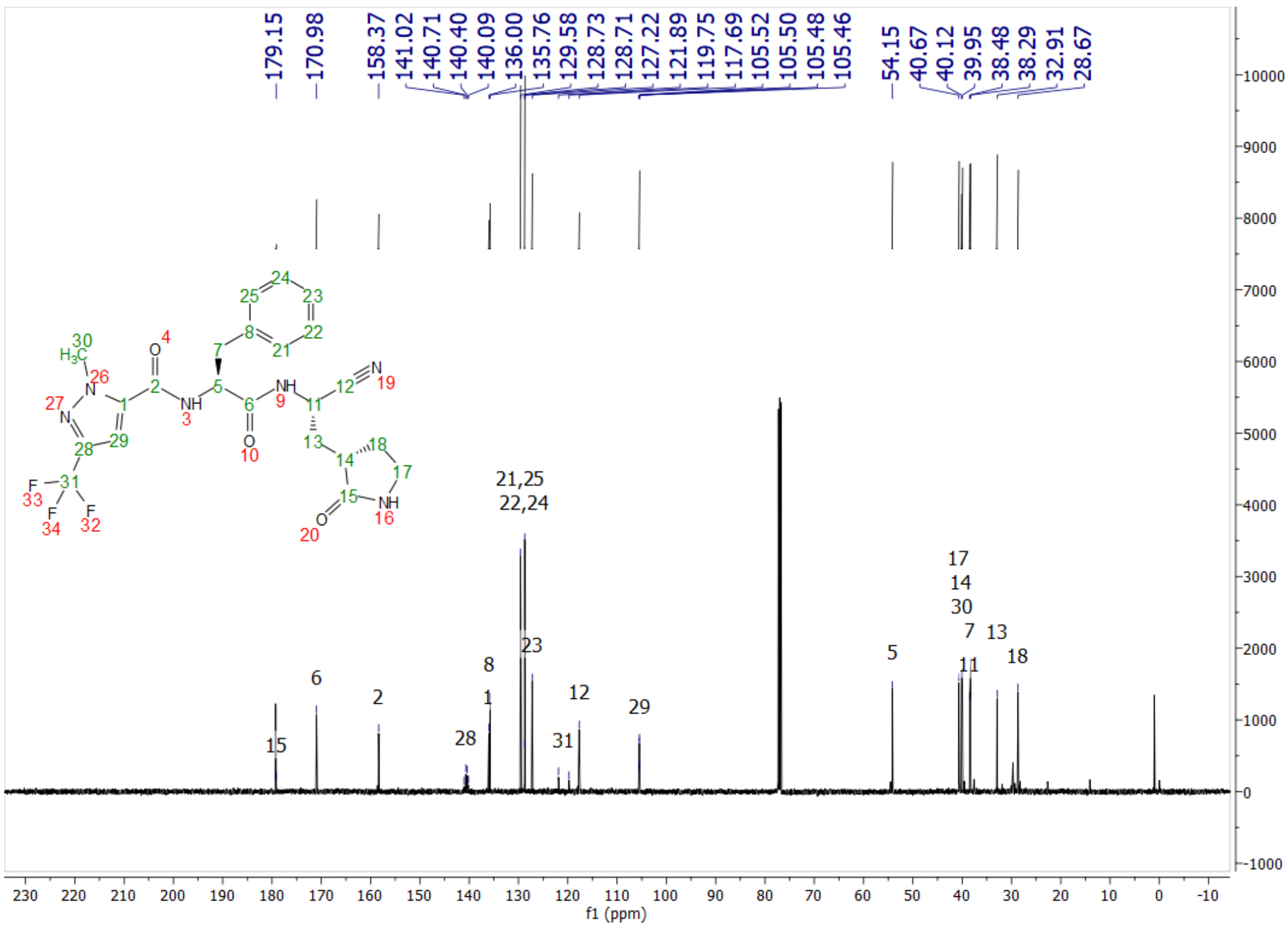

Figure S3. ^{13}C NMR Spectrum of Neq1181.

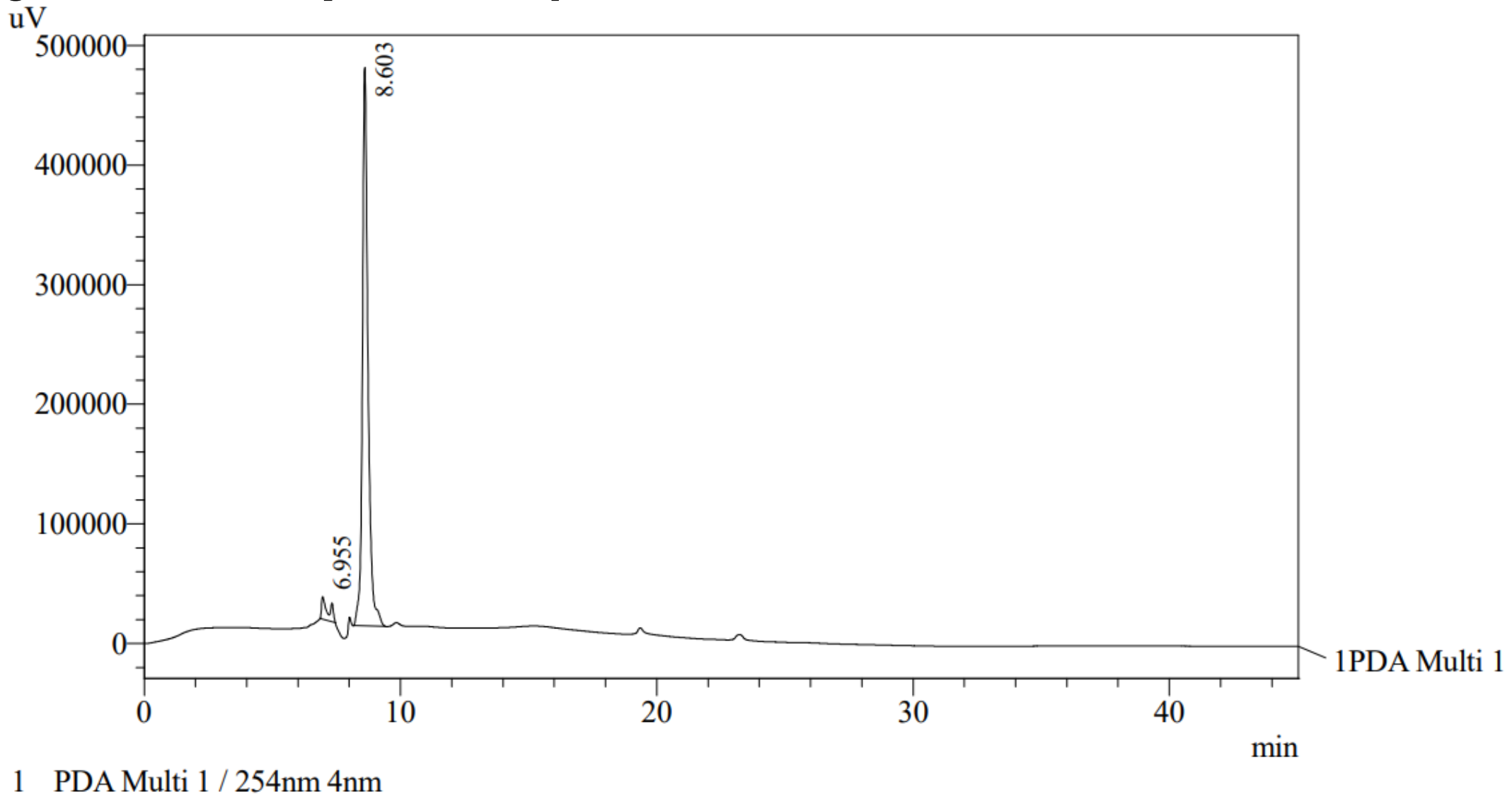

PeakTable

PDA Ch1 254nm 4nm

Peak#	Ret. Time	Area	Height	Area %	Height %
1	6.955	339716	19002	4.574	3.911
2	8.603	7087456	466826	95.426	96.089
Total		7427172	485828	100.000	100.000

Figure S4. HPLC chromatogram of Neq1181.

White solid. Melting point: 106-108 ºC. ^{1}H NMR (400 MHz, cdcl3) δ 8.55 (d, J = 5.8 Hz, 1H), 7.28 (dd, J = 6.9, 1.2 Hz, 2H), 7.24 (s, 3H), 7.22 – 7.15 (m, 2H), 6.94 (d, J = 0.7 Hz, 1H), 4.95 (dt, J = 8.6, 6.1 Hz, 1H), 4.63 (dt, J =

10.8, 5.6 Hz, 1H), 4.14 (s, 3H), 3.25 (dd, J = 13.7, 5.9 Hz, 1H), 3.09 (dd, J = 13.7, 6.3 Hz, 1H), 2.36 (dt, J = 13.0, 6.9 Hz, 1H), 2.22 – 2.11 (m, 2H), 2.01 – 1.91 (m, 1H), 1.90 – 1.78 (m, 1H). ^{13}C NMR (126 MHz, $CDCl_3$) δ 179.15, 170.98, 158.37, 136.00, 135.76, 129.58, 128.73, 127.22, 117.69, 105.49 (q, *J* = 2.2 Hz), 54.15, 40.67, 40.12, 39.95, 38.48, 38.29, 32.91, 28.67. ESI-MS $[M + H]^+$ = 477.6. HPLC chiralpak IB column 5 μm, 4.6mm Ø x 250 mm, gradient: HEX/IPA 5-100%, flow 0,5 $ml.min^{-1}$, 25 °C, 40 minutes. T_R = 8.6 minutes, purity: 95.42%.

Biochemical assays

K_M^{hCatL}	K_M^{hCatL}
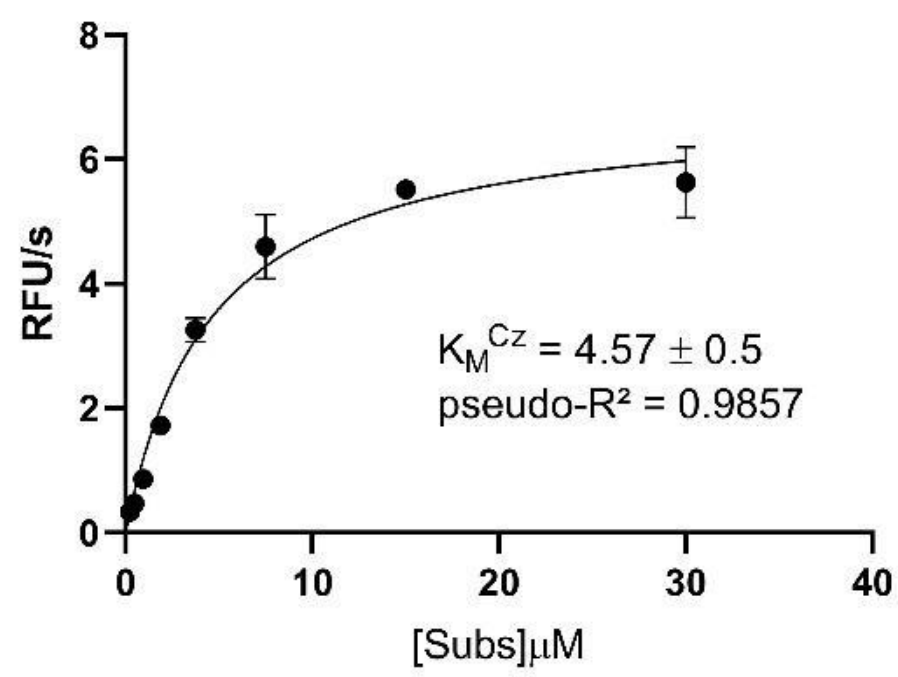	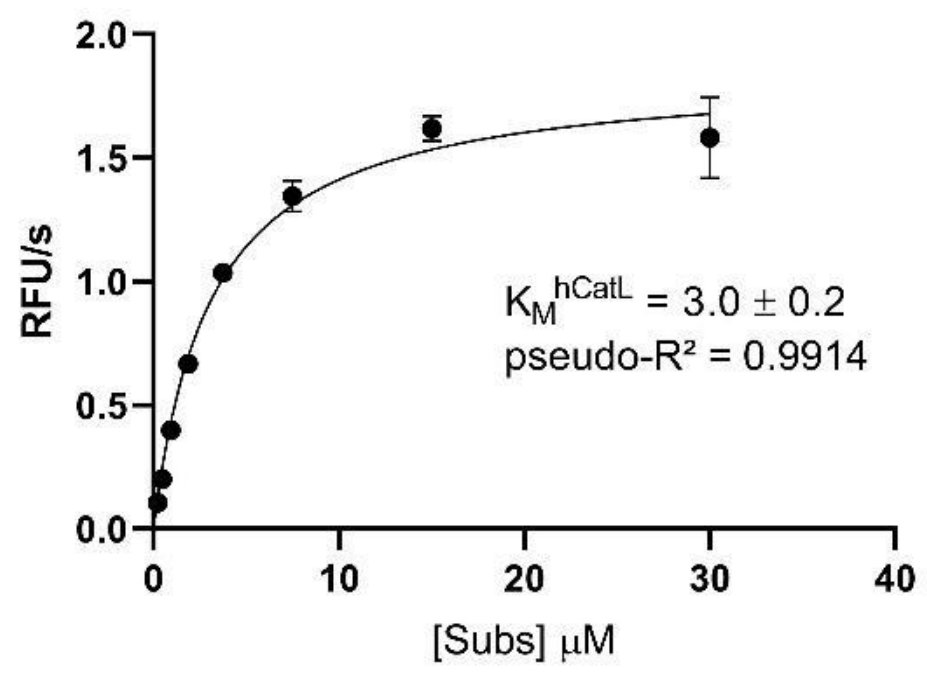

pK_i^{Cz}	pK_i^{hCatL}
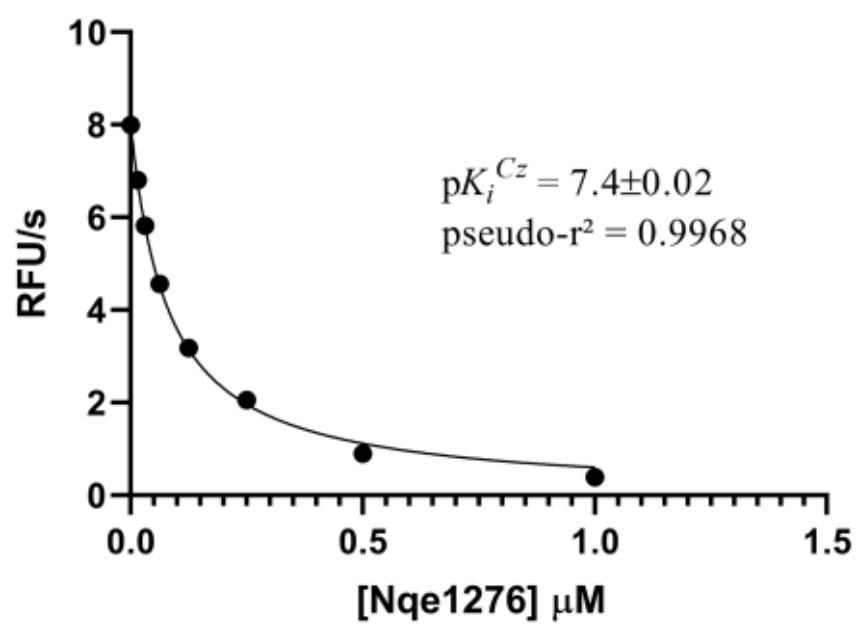	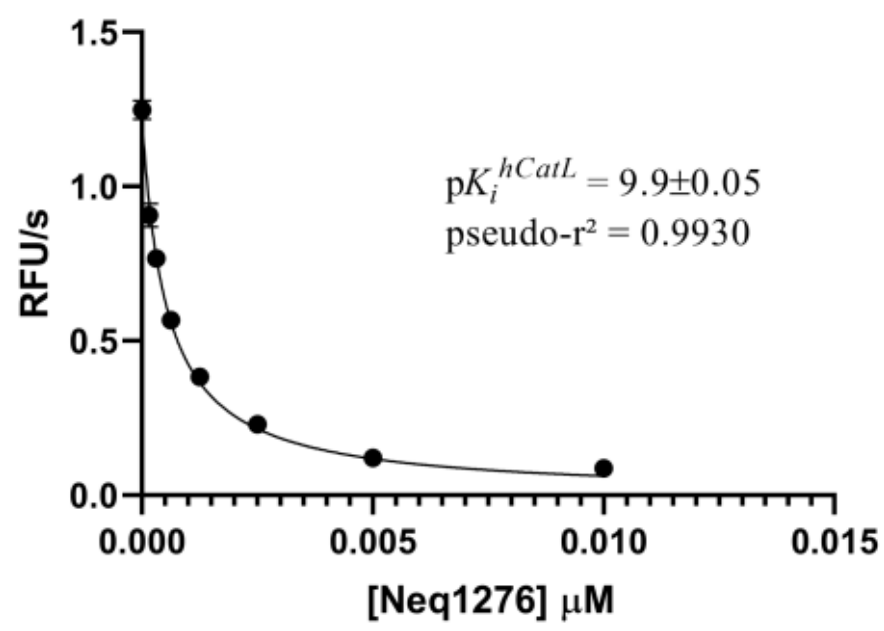
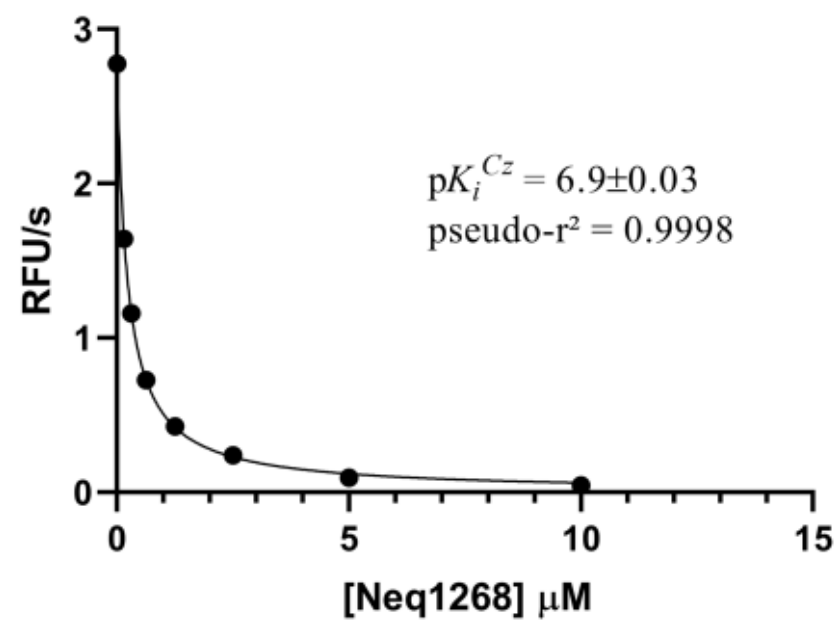	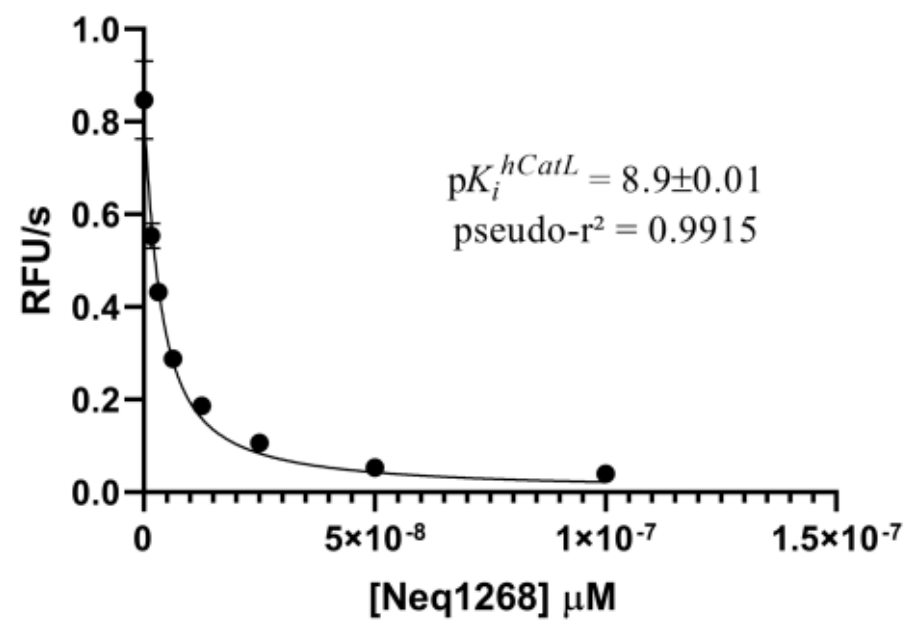

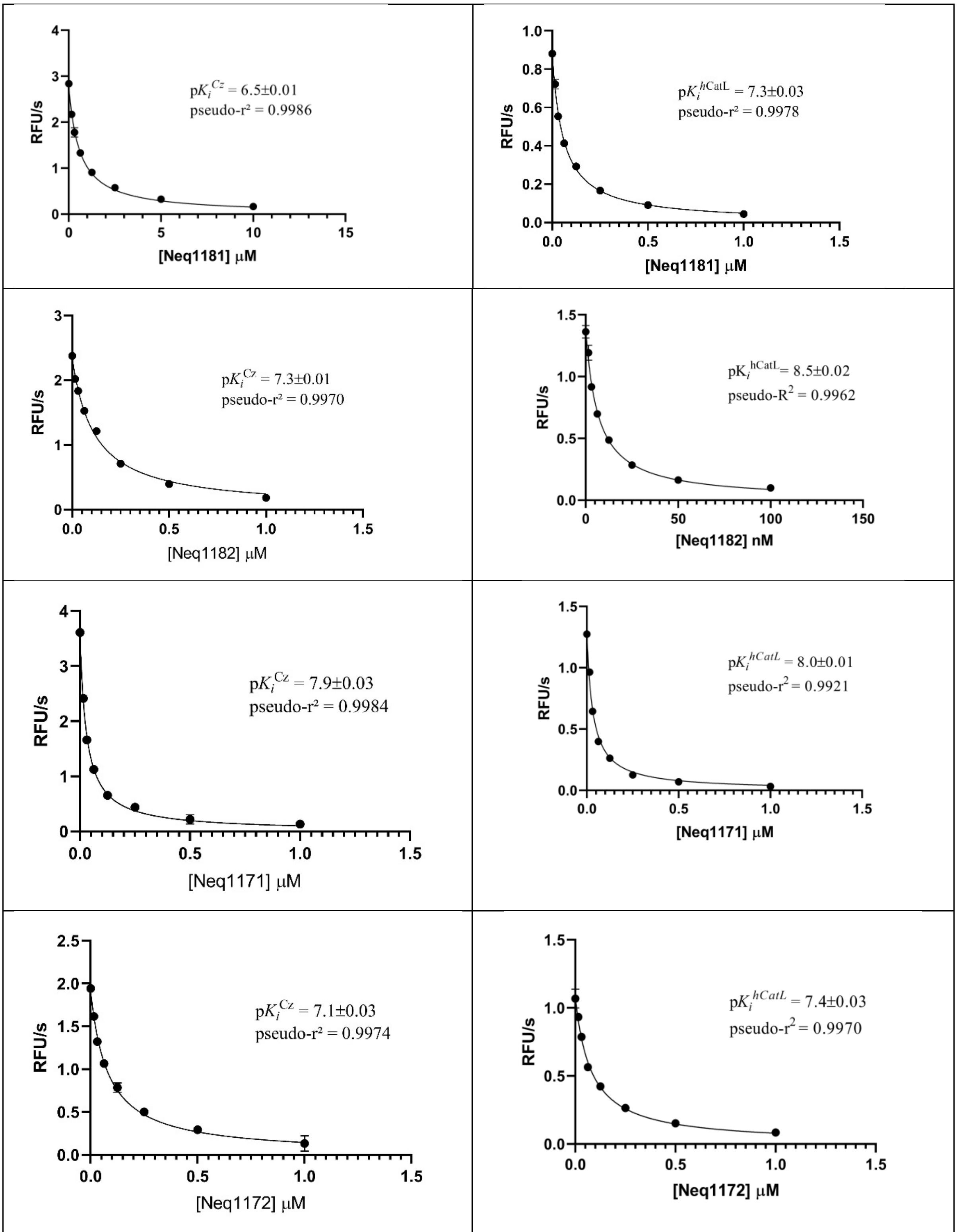

Figure S5. Biochemical assays of compounds against Cz and hCatL[1]

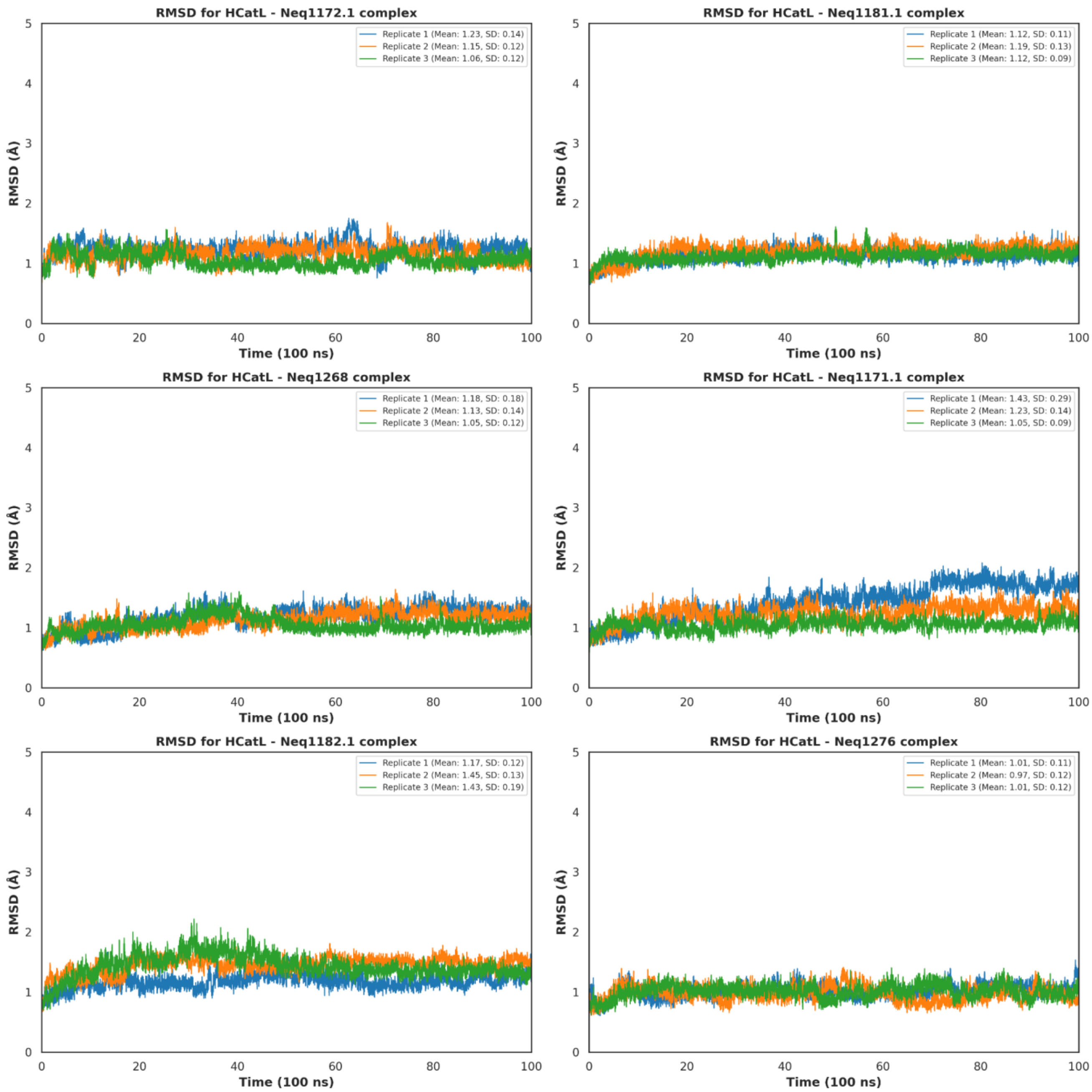

Figure S6. Root-mean-square deviation (RMSD) plots over 100 ns of simulation for the hCatL enzyme complexes with the Neq ligand series (Neq1172.1, Neq1181.1, Neq1268, Neq1171.1, Neq1182.1, Neq1276) in triplicate.

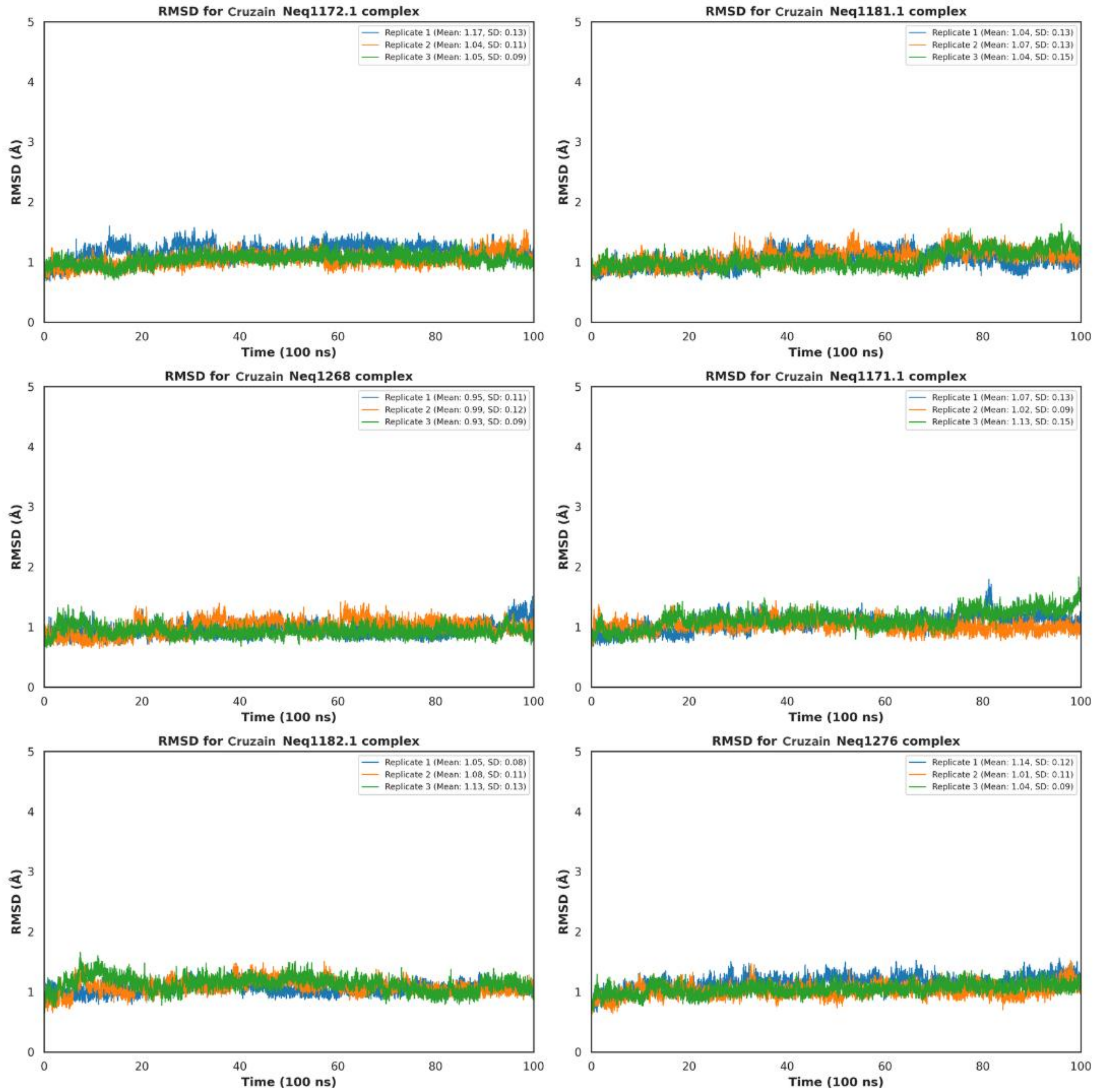

Figure S7. Root-mean-square deviation (RMSD) plots over 100 ns of simulation for the cruzain enzyme complexes with the Neq ligand series (Neq1172.1, Neq1181.1, Neq1268, Neq1171.1, Neq1182.1, Neq1276) in triplicate.

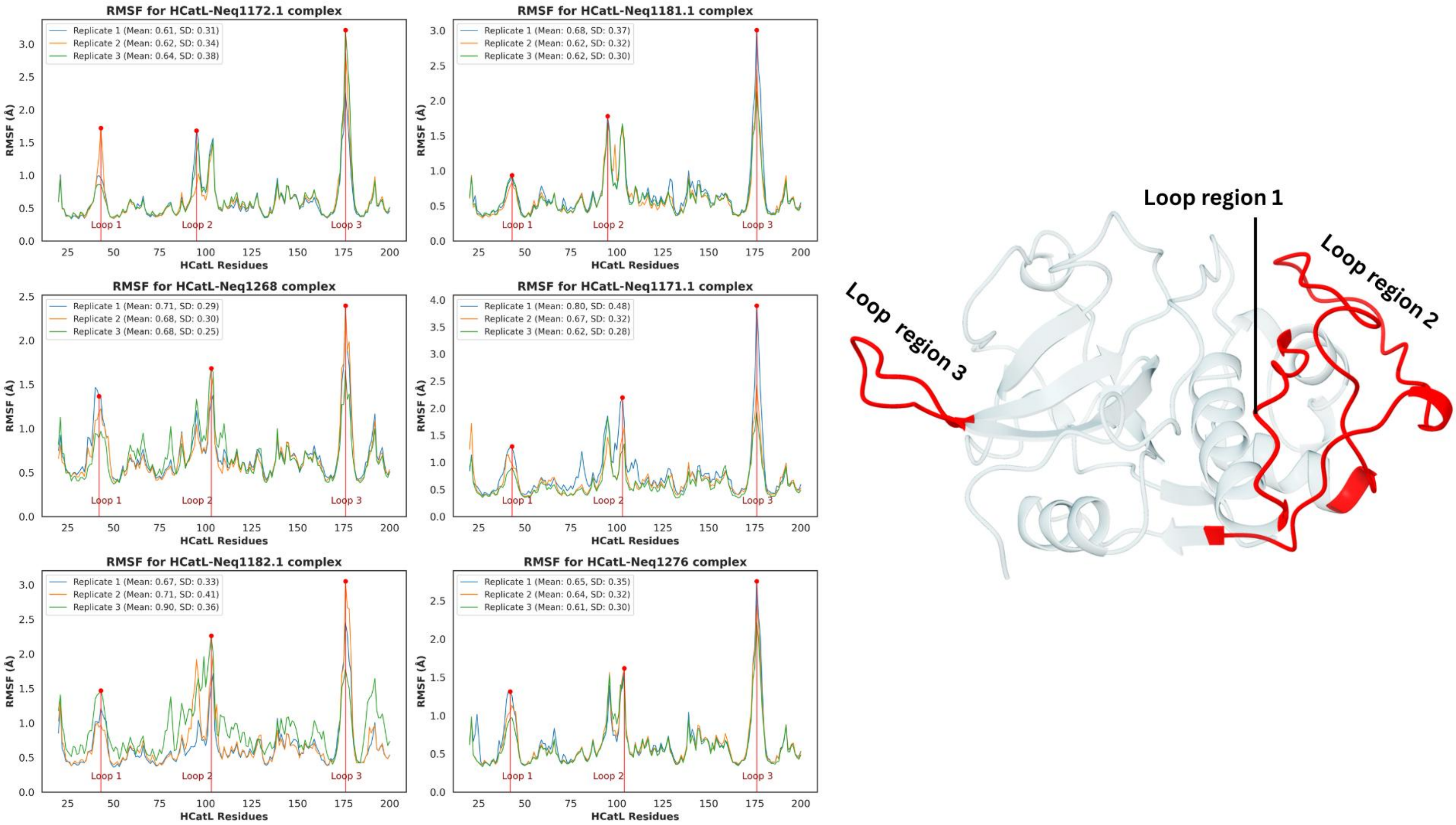

Figure S8. Root-mean-square fluctuation (RMSF) analysis per residue for the hCatL complexes, with a three-dimensional structural projection highlighting the regions of greatest flexibility (Loops 1, 2, and 3).

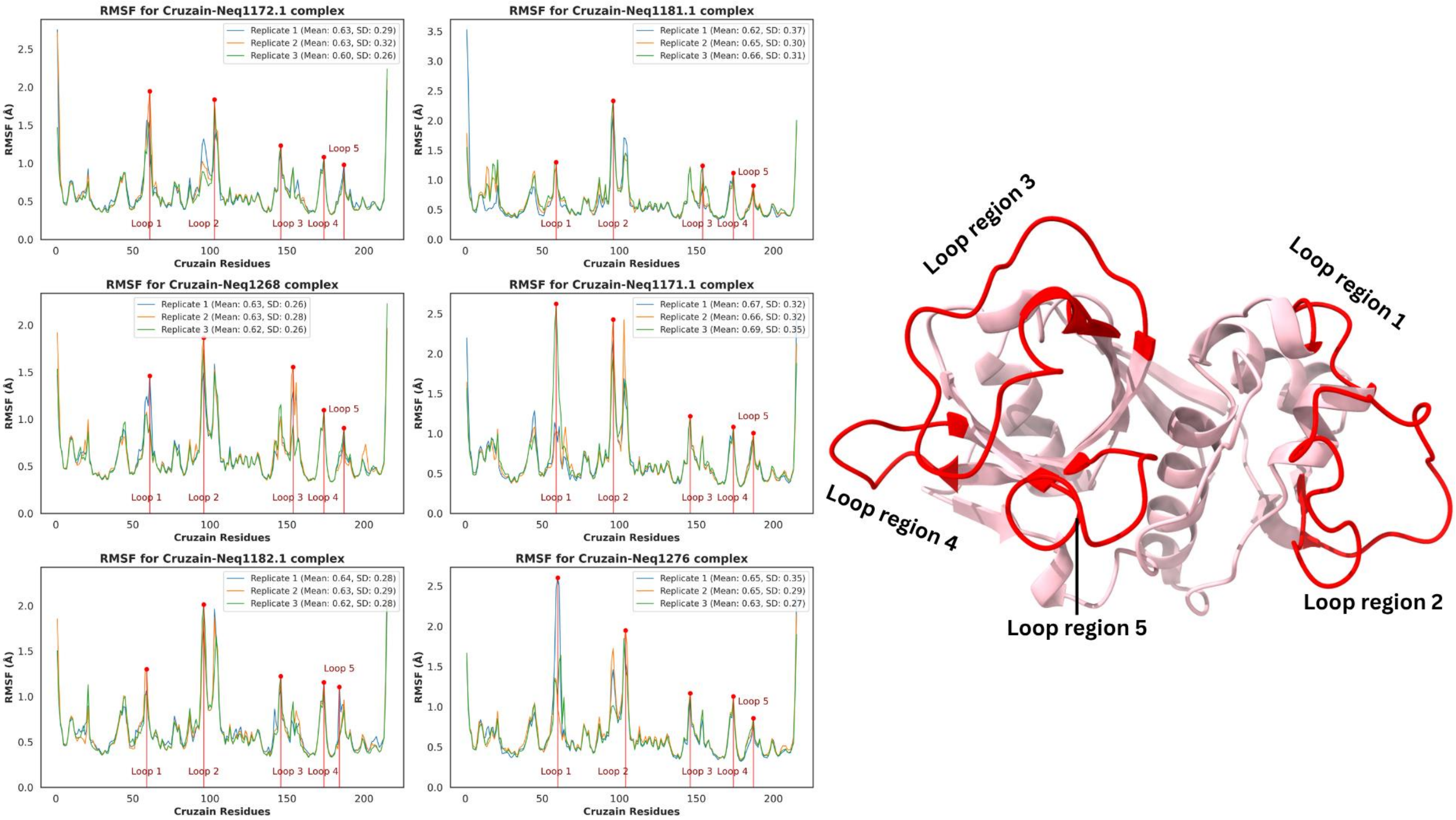

Figure S9. Root-mean-square fluctuation (RMSF) analysis per residue for the cruzain complexes, with a three-dimensional structural projection highlighting the regions of greatest flexibility (Loops 1 to 5).

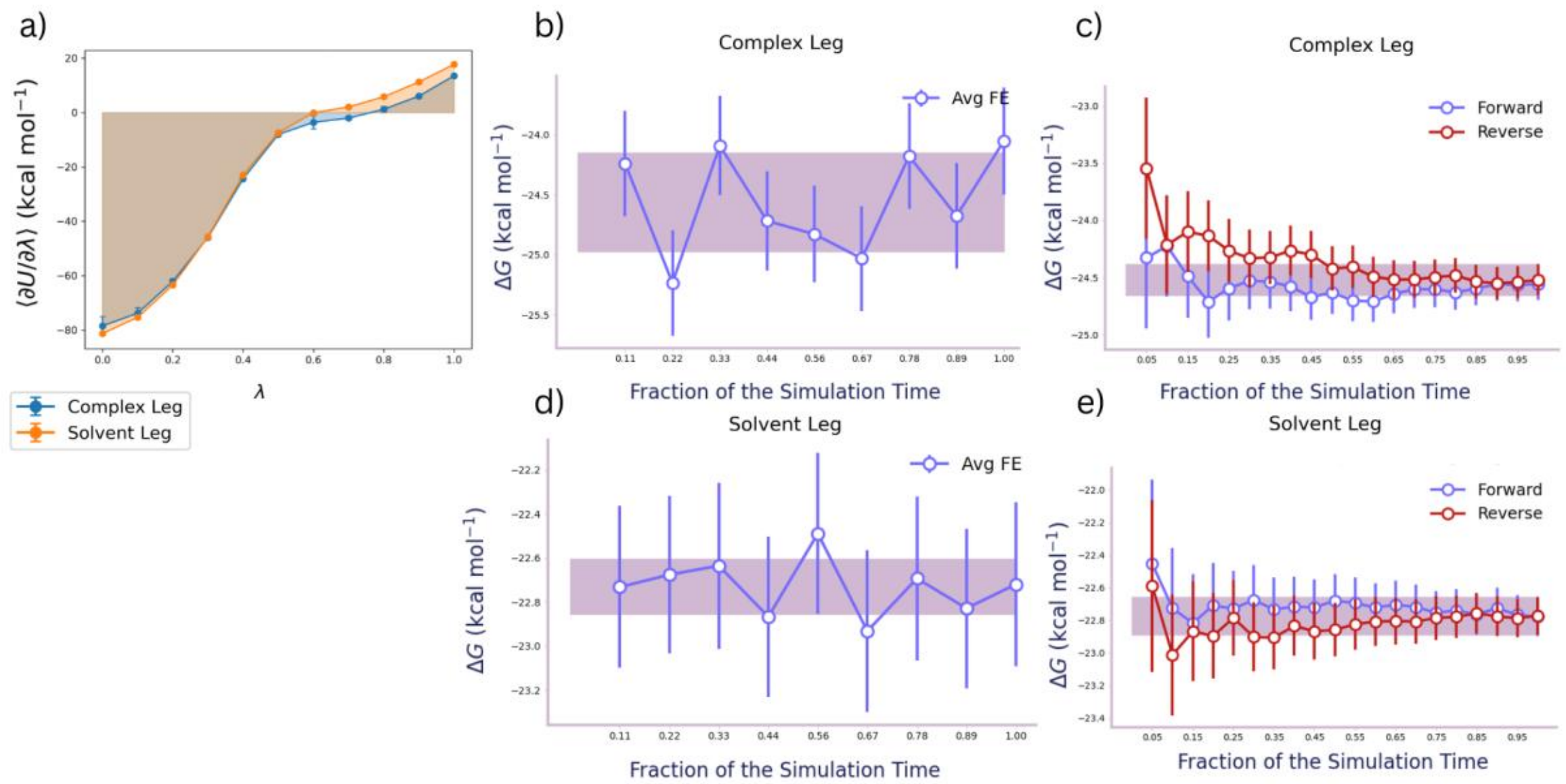

Figure S10. Free energy convergence analysis for the alchemical transformation from cruzain-) as a function of the coupling parameter λ for both the complex and solvent phases. (b) Block average convergence analysis and (c) Forward-Backward bidirectional convergence estimate of the free energy variation (ΔG) for the complex phase. (d) Block average convergence analysis and (e) Forward-Backward bidirectional convergence estimate of ΔG for the solvent leg.

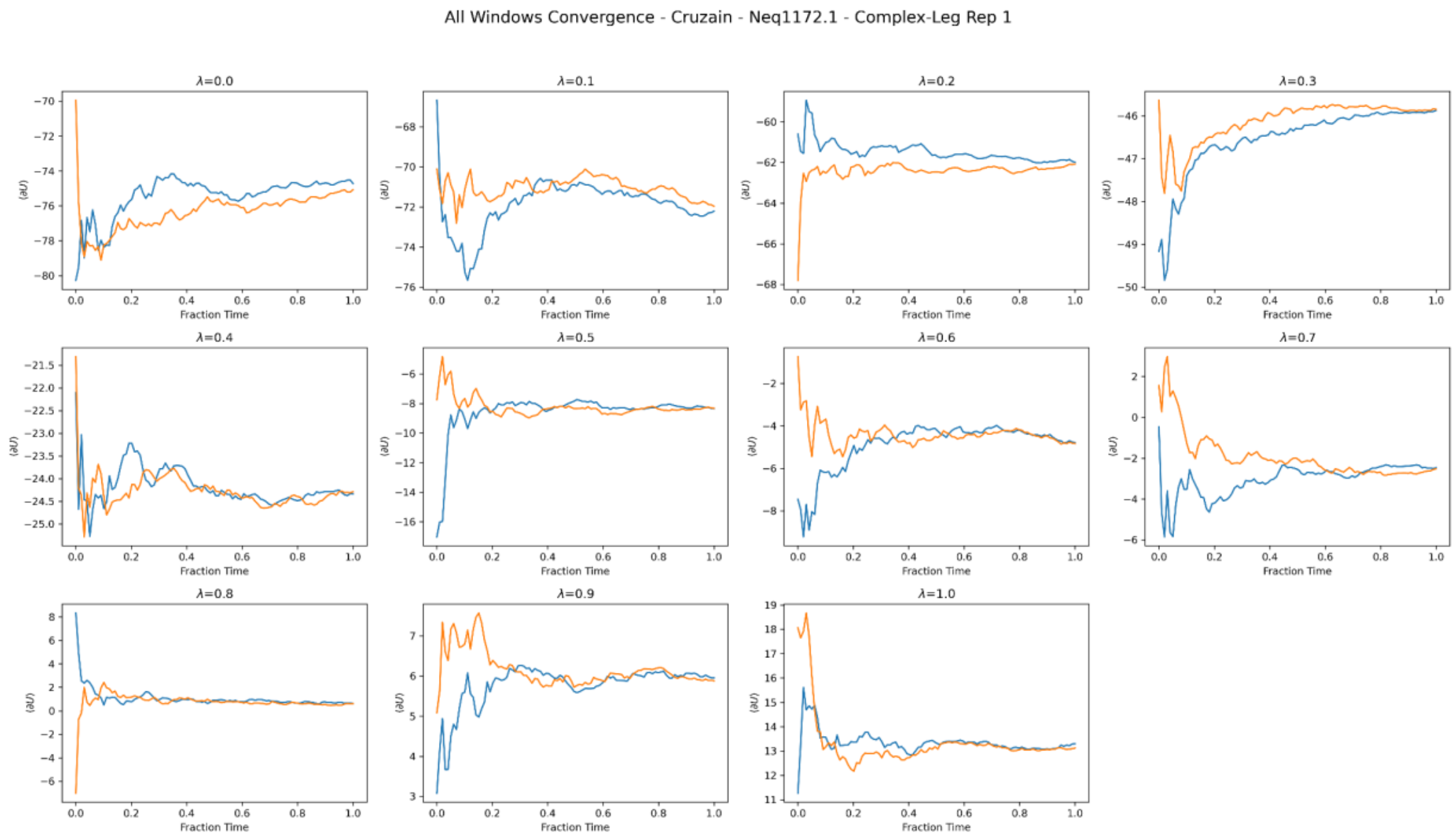

Figure S11. Temporal convergence profile of ⟨∂U/∂λ⟩ across all simulated λ windows in the complex step for the alchemical transformation from cruzain-Neq1172.1 to cruzain-Neq1171.1, (replicate 1).

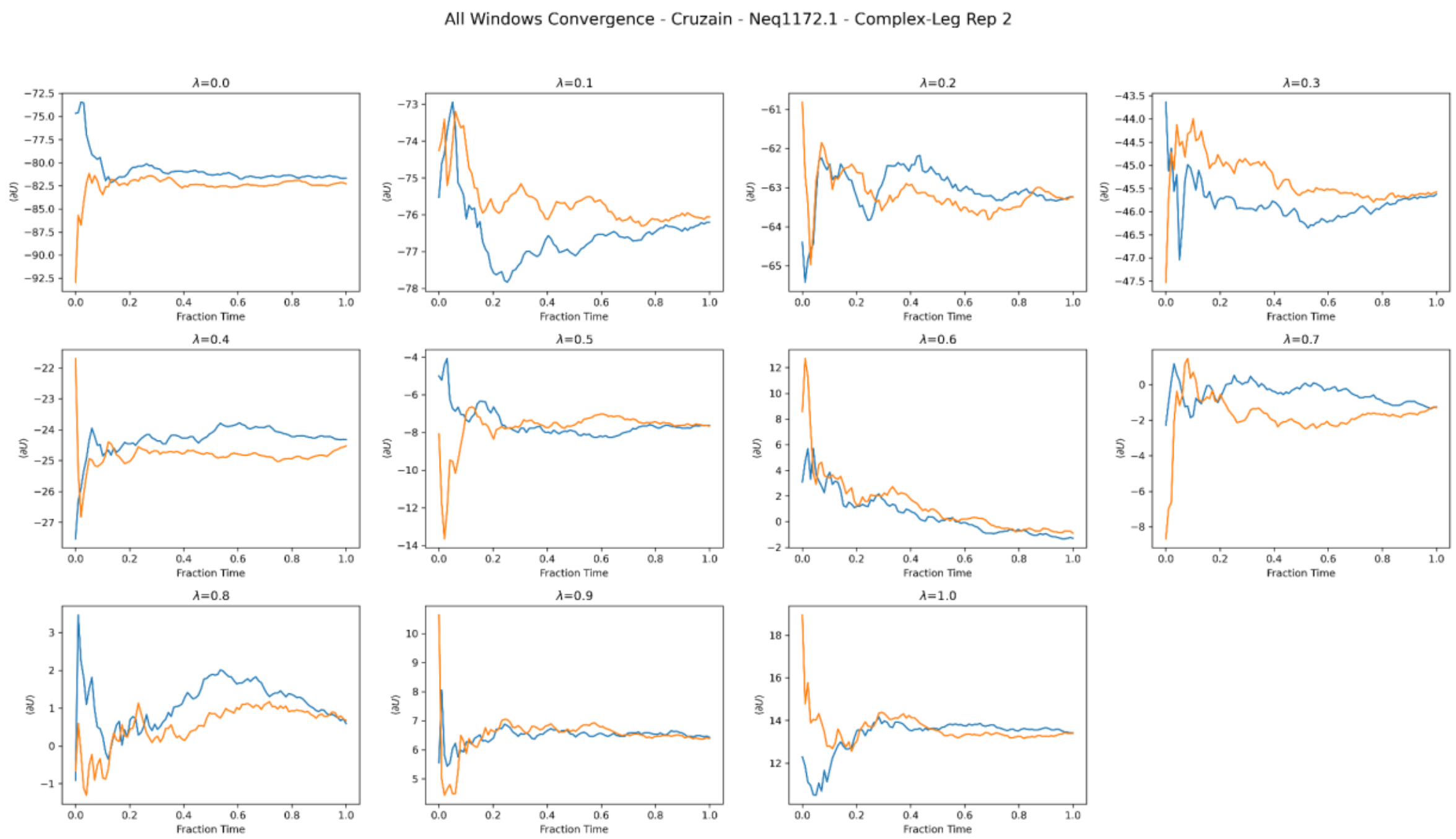

Figure S12. Temporal convergence profile of ⟨∂U/∂λ⟩ across all simulated λ windows in the complex step for the alchemical transformation from cruzain-Neq1172.1 to cruzain-Neq1171.1, (replicate 2).

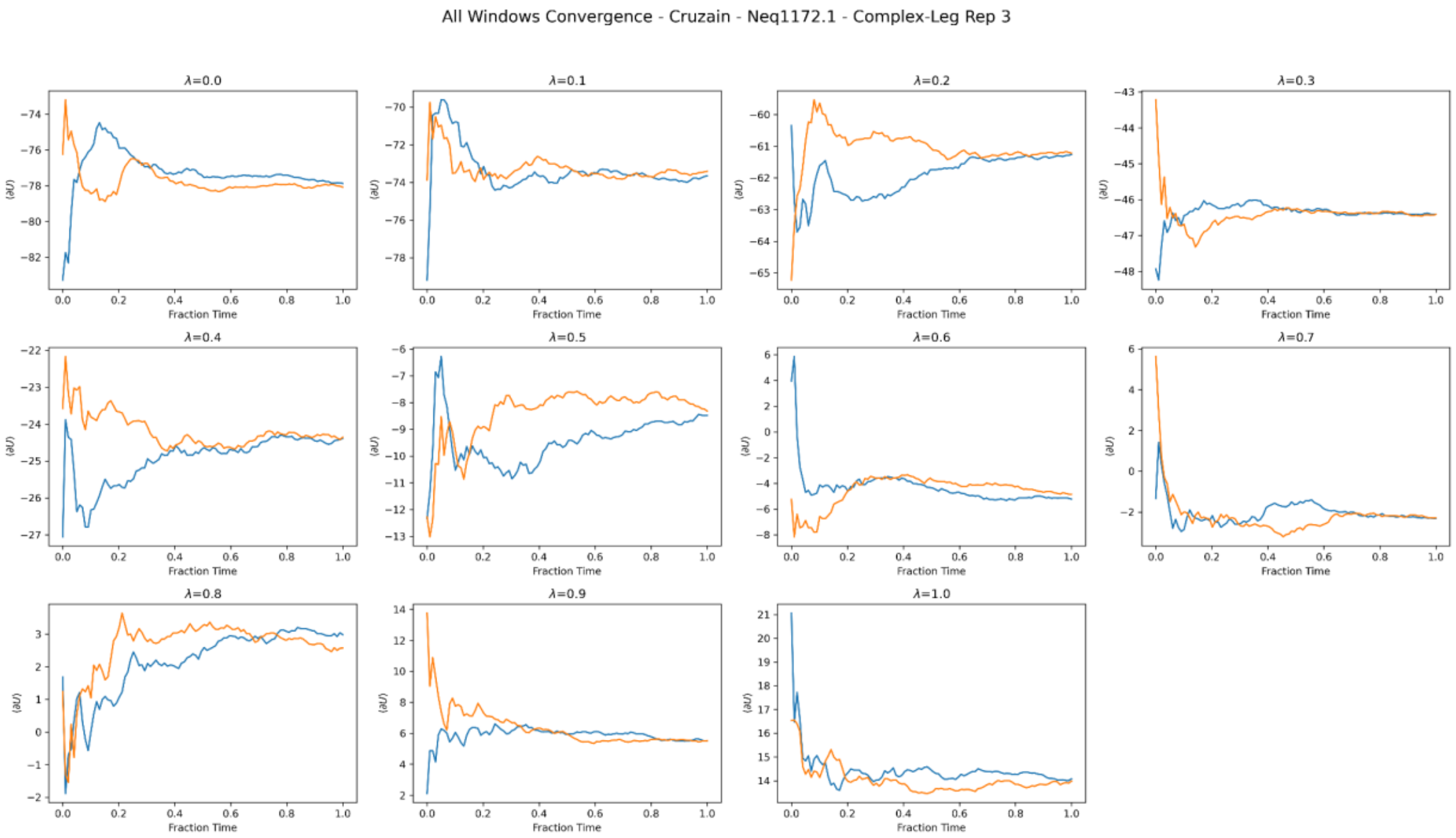

Figure S13. Temporal convergence profile of ⟨∂U/∂λ⟩ across all simulated λ windows in the complex step for the alchemical transformation from cruzain-Neq1172.1 to cruzain-Neq1171.1, (replicate 3).

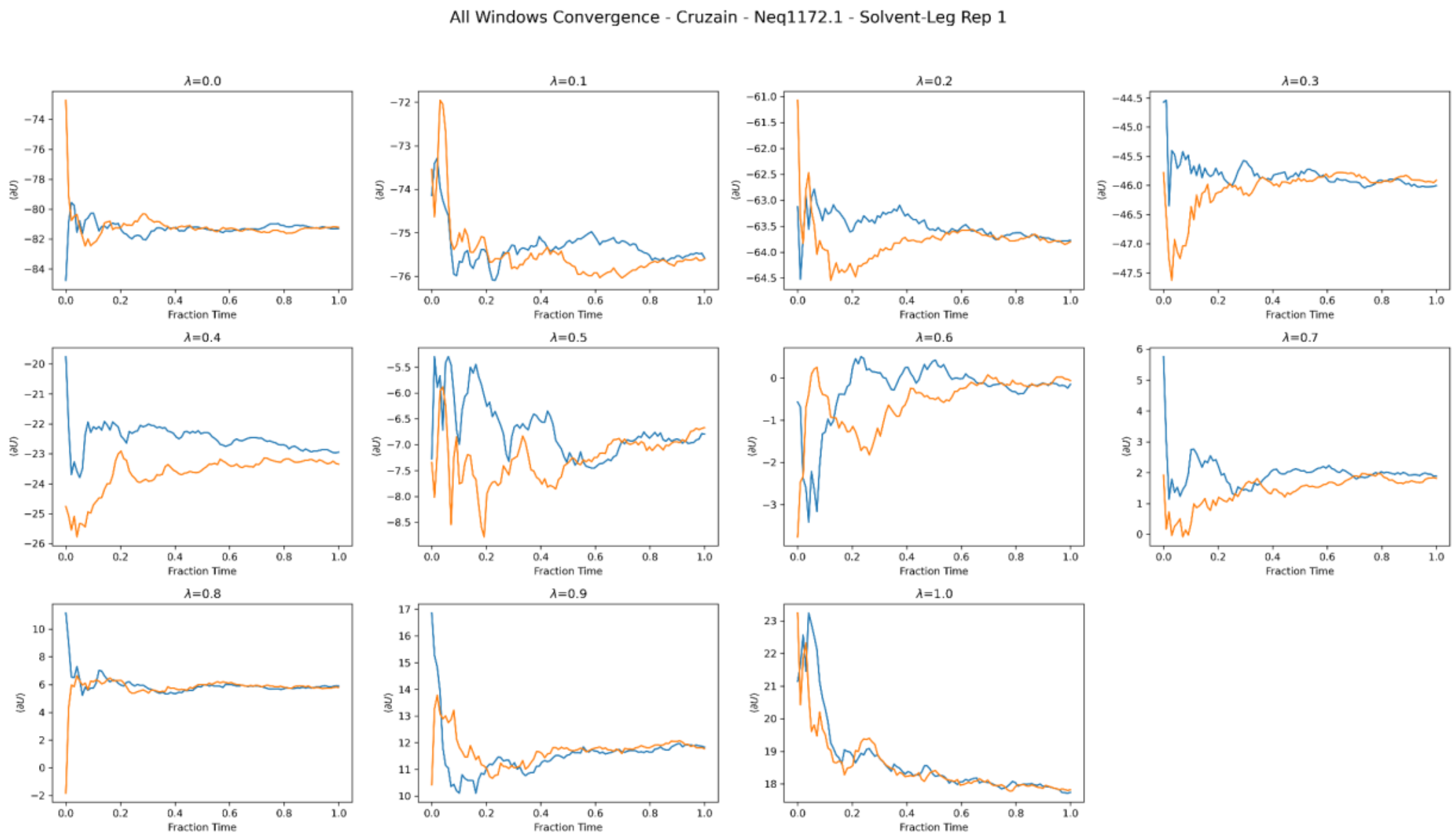

Figure S14. Temporal convergence profile of ⟨∂U/∂λ⟩ across all simulated λ windows in the solvent step for the alchemical transformation from cruzain–Neq1172.1 to cruzain–Neq1171.1, (replicate 1).

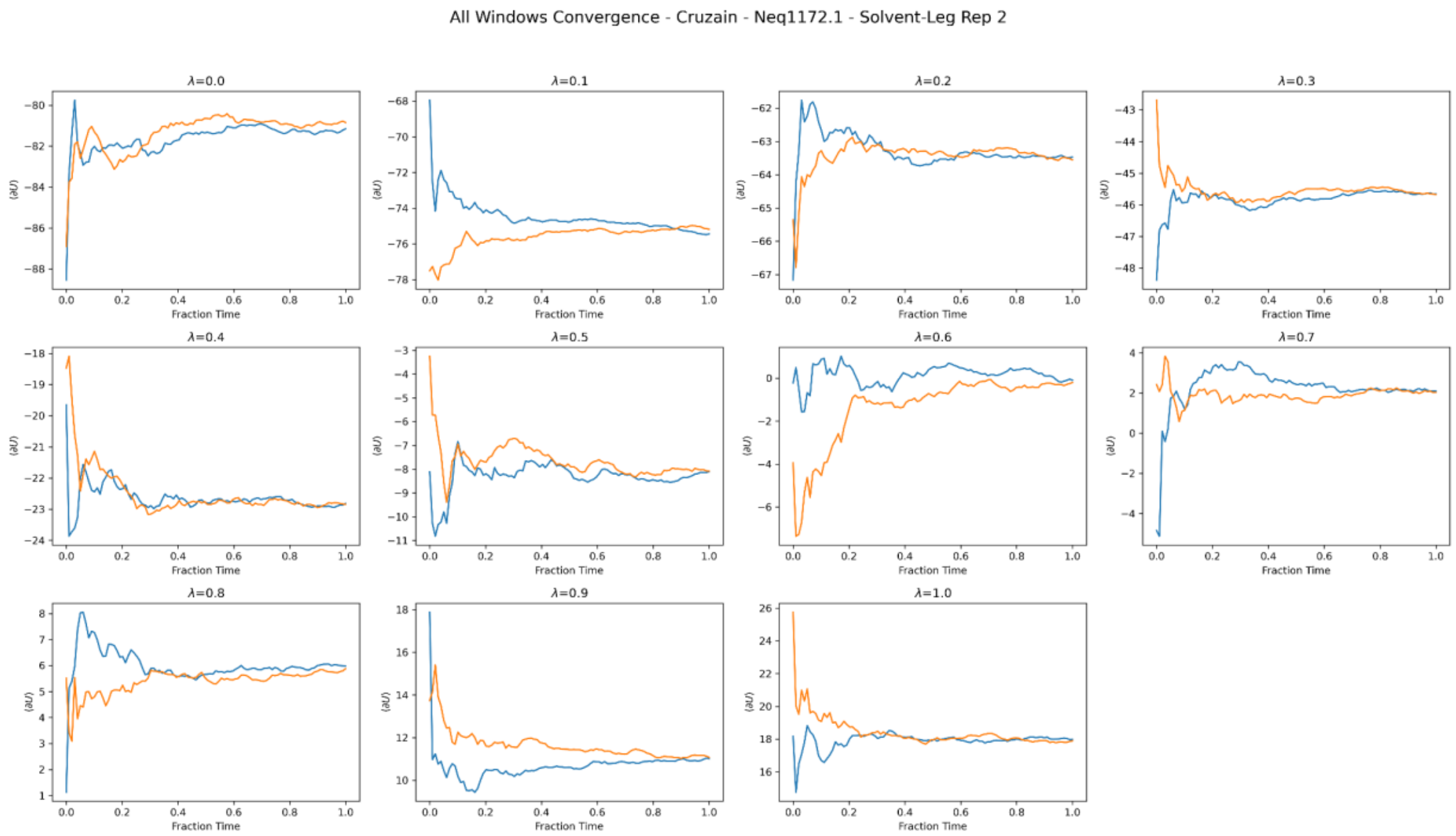

Figure S15. Temporal convergence profile of ⟨∂U/∂λ⟩ across all simulated λ windows in the solvent step for the alchemical transformation from cruzain–Neq1172.1 to cruzain–Neq1171.1, (replicate 2).

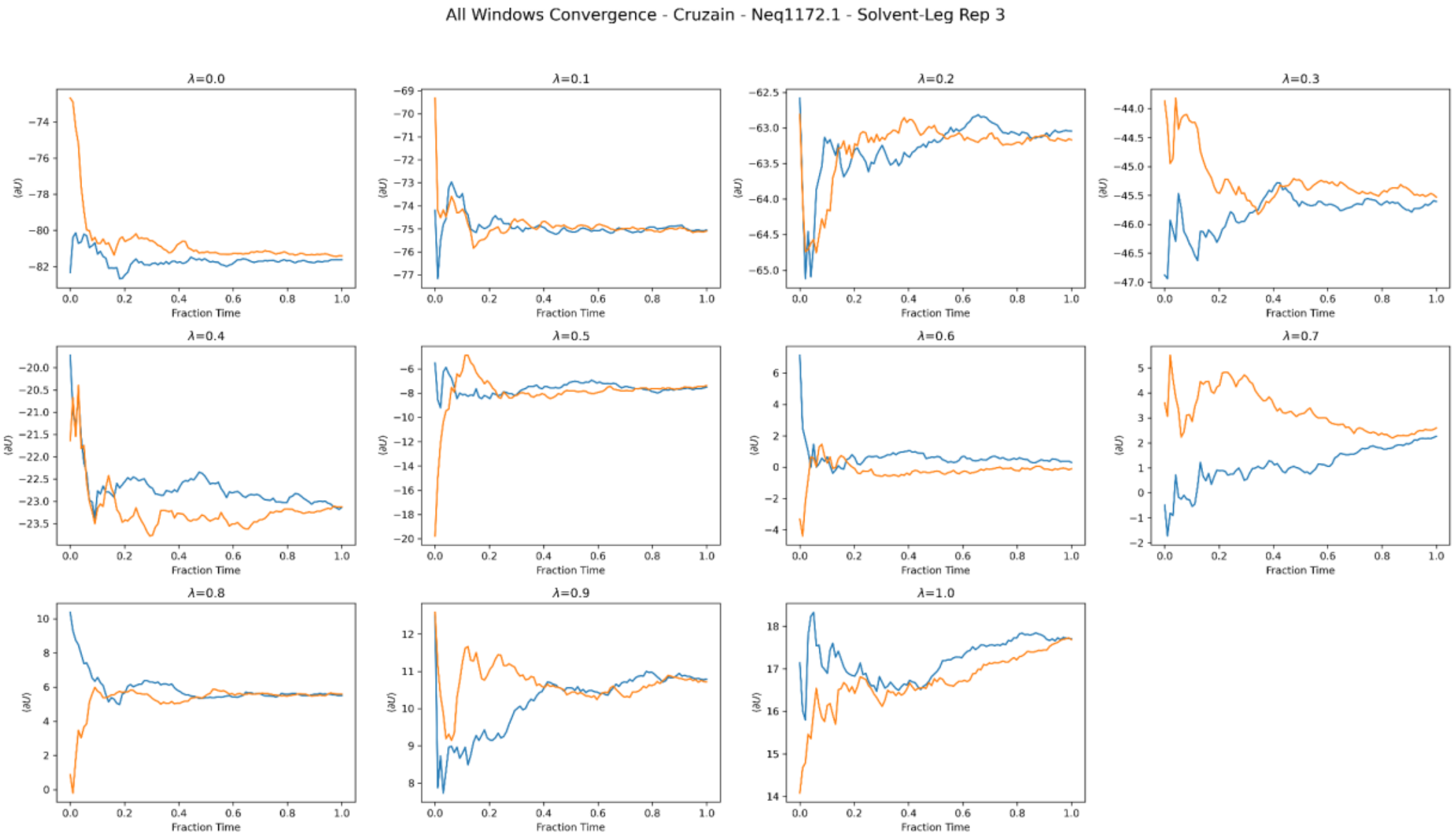

Figure S16. Temporal convergence profile of ⟨∂U/∂λ⟩ across all simulated λ windows in the solvent step for the alchemical transformation from cruzain–Neq1172.1 to cruzain–Neq1171.1, (replicate 3).

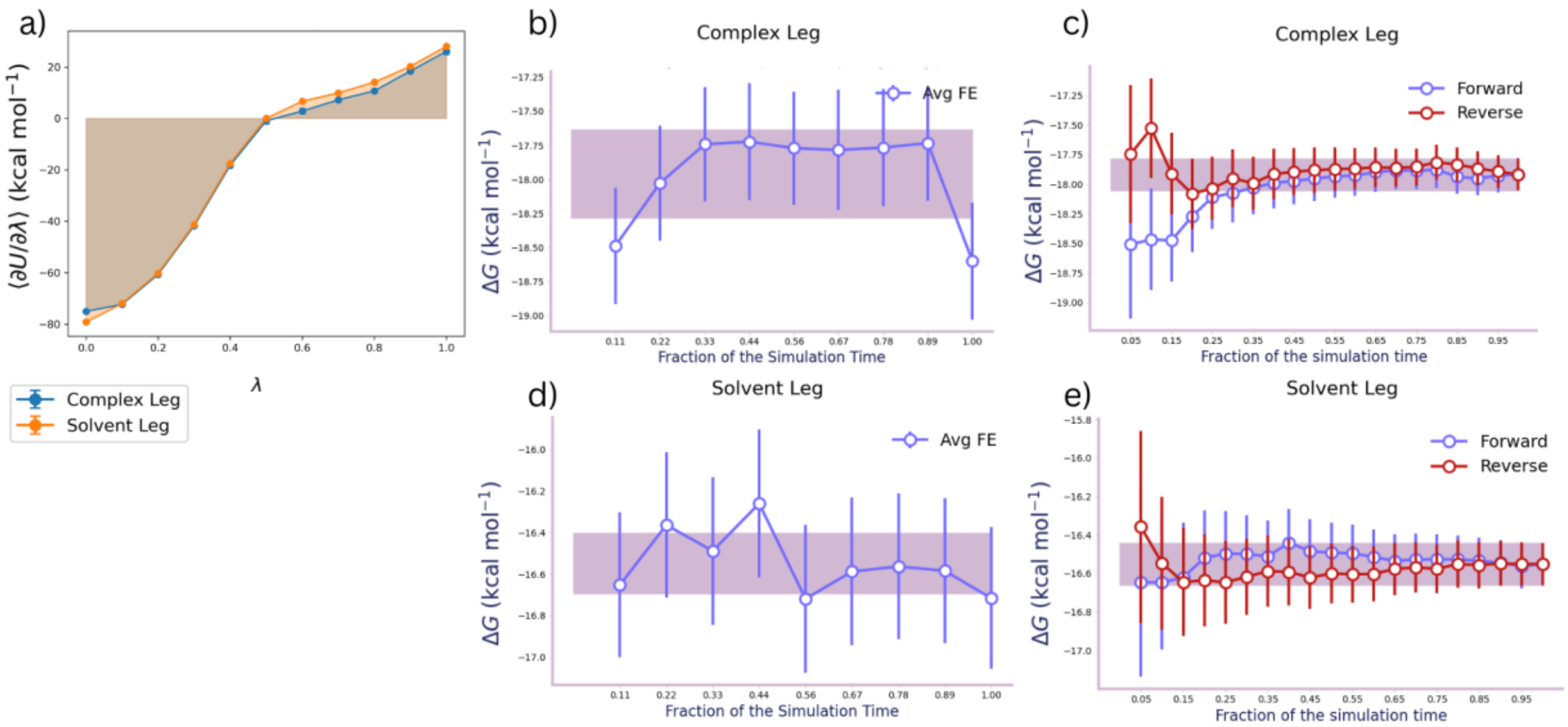

Figure S17. Free energy convergence analysis for the alchemical transformation from cruzain–) as a function of the coupling parameter **λ** for both the complex and solvent phases. (b) Block average convergence analysis and (c) Forward–Backward bidirectional convergence estimate of the free energy variation (ΔG) for the complex phase. (d) Block average convergence analysis and (e) Forward–Backward bidirectional convergence estimate of ΔG for the solvent leg.

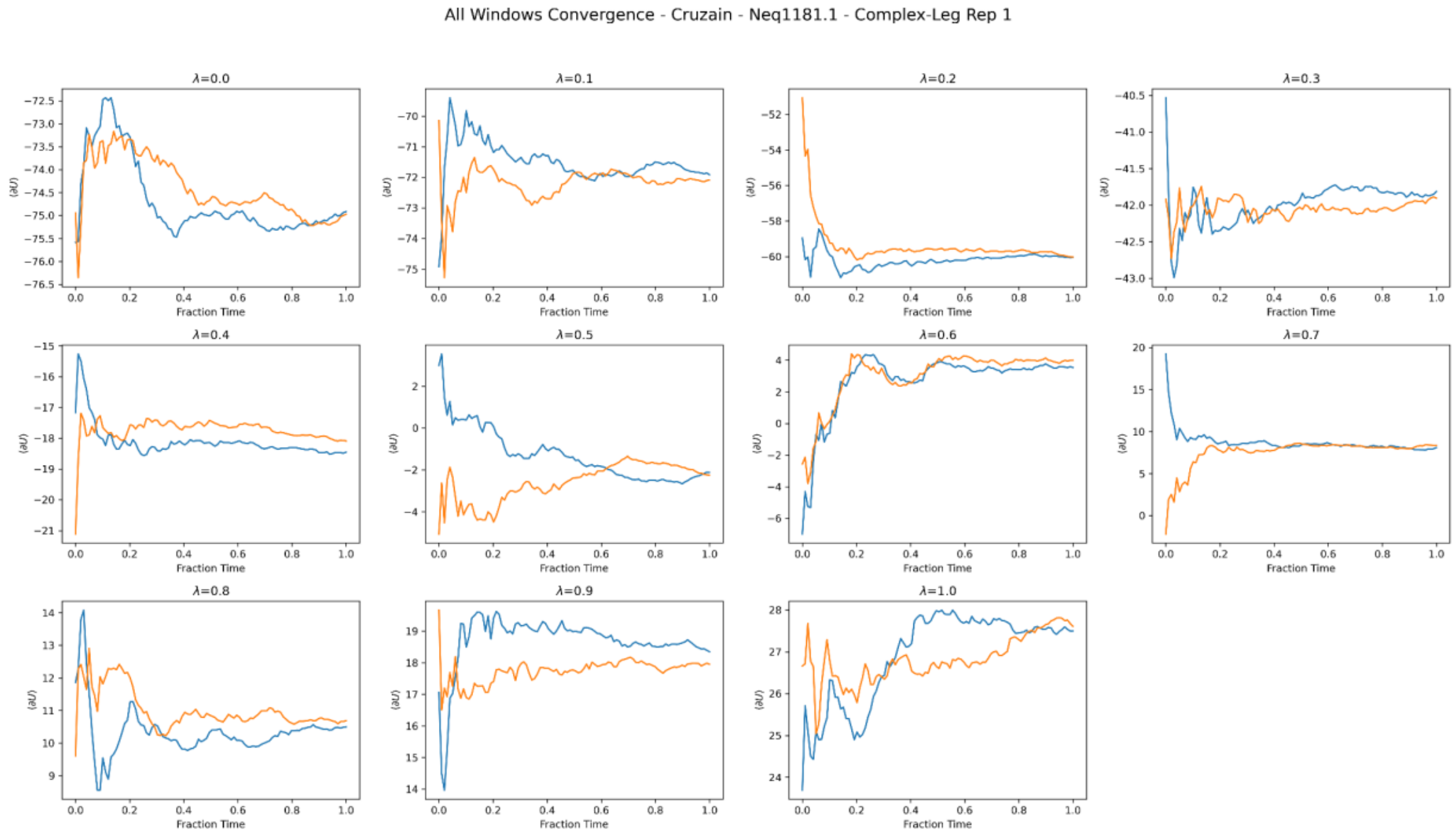

Figure S18. Temporal convergence profile of ⟨∂U/∂λ⟩ across all simulated λ windows in the complex step for the alchemical transformation from cruzain–Neq1181.1 to cruzain–Neq1182.1, (replicate 1).

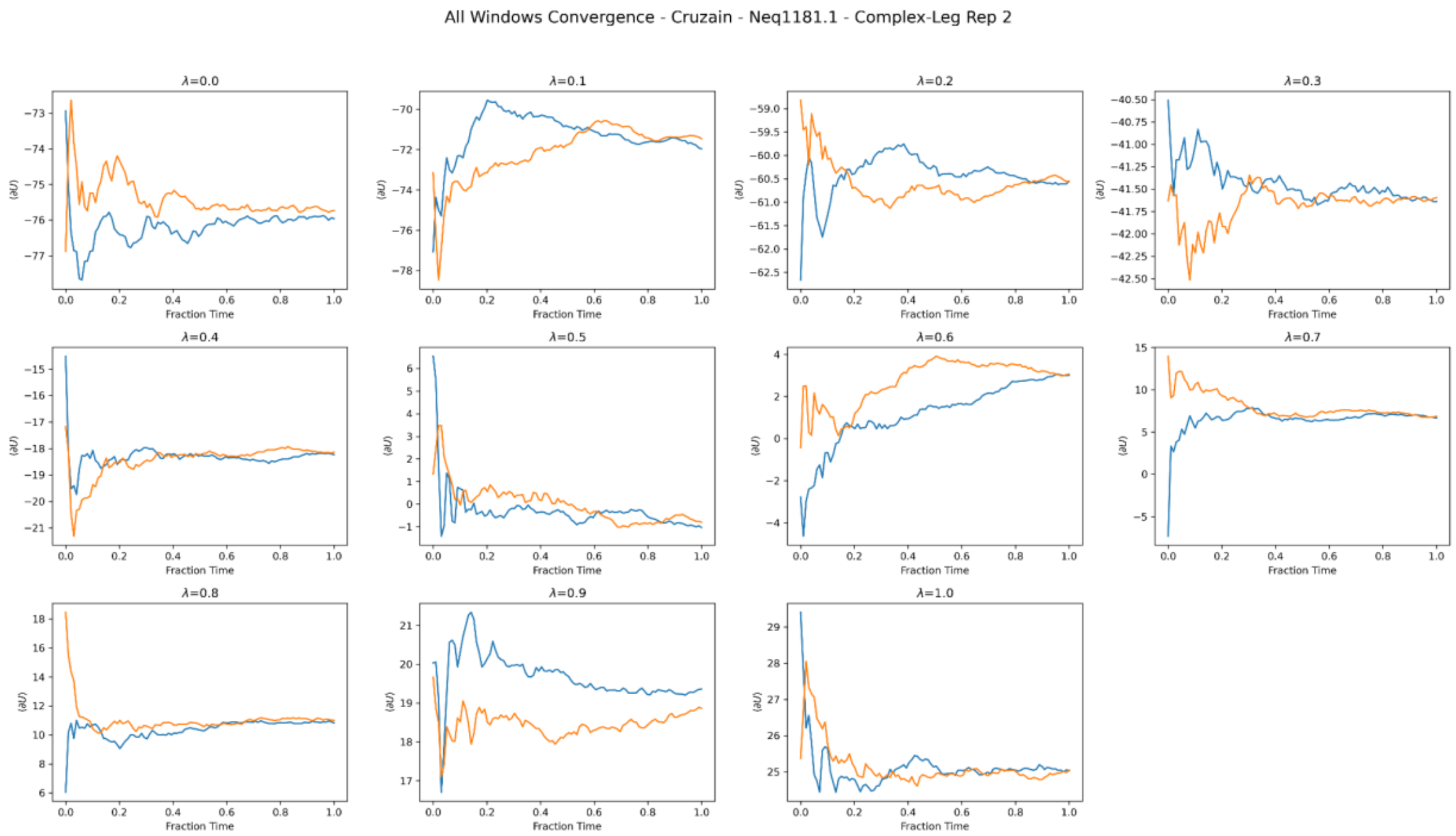

Figure S19. Temporal convergence profile of ⟨∂U/∂λ⟩ across all simulated λ windows in the complex step for the alchemical transformation from cruzain–Neq1181.1 to cruzain–Neq1182.1, (replicate 2).

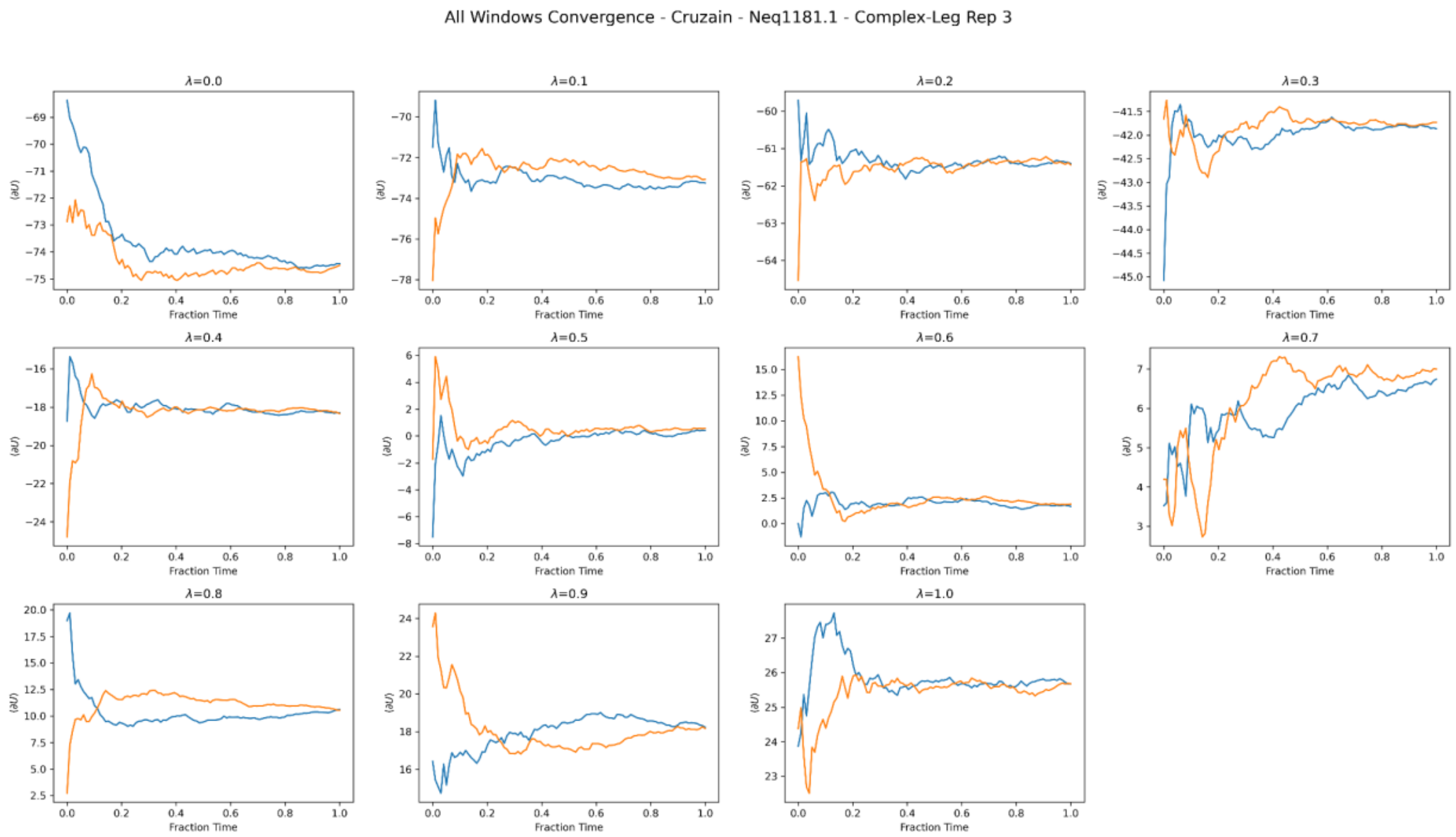

Figure S20. Temporal convergence profile of ⟨∂U/∂λ⟩ across all simulated λ windows in the complex step for the alchemical transformation from cruzain–Neq1181.1 to cruzain–Neq1182.1, (replicate 3).

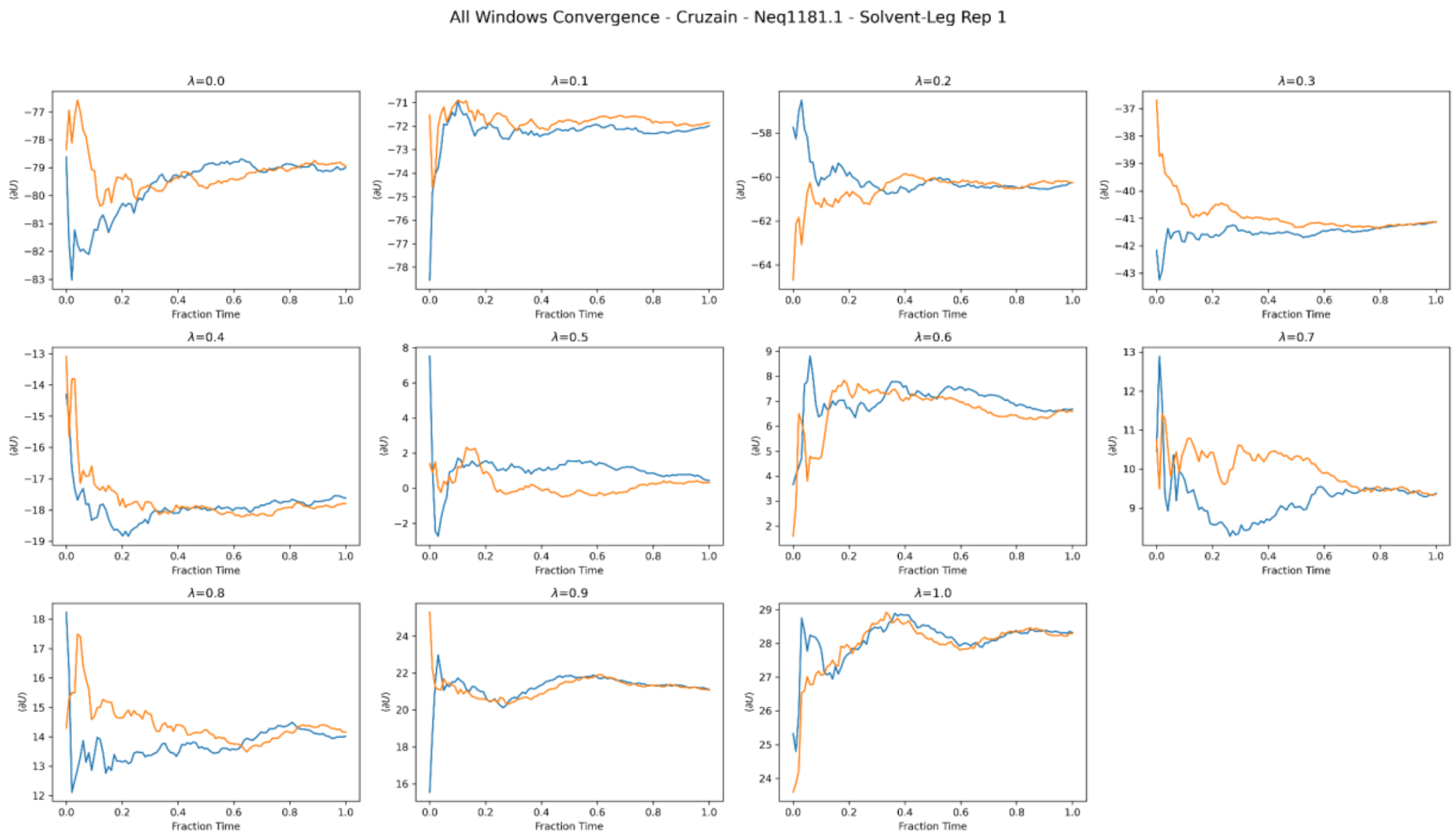

Figure S21. Temporal convergence profile of ⟨∂U/∂λ⟩ across all simulated λ windows in the solvent step for the alchemical transformation from cruzain–Neq1181.1 to cruzain–Neq1182.1, (replicate 1).

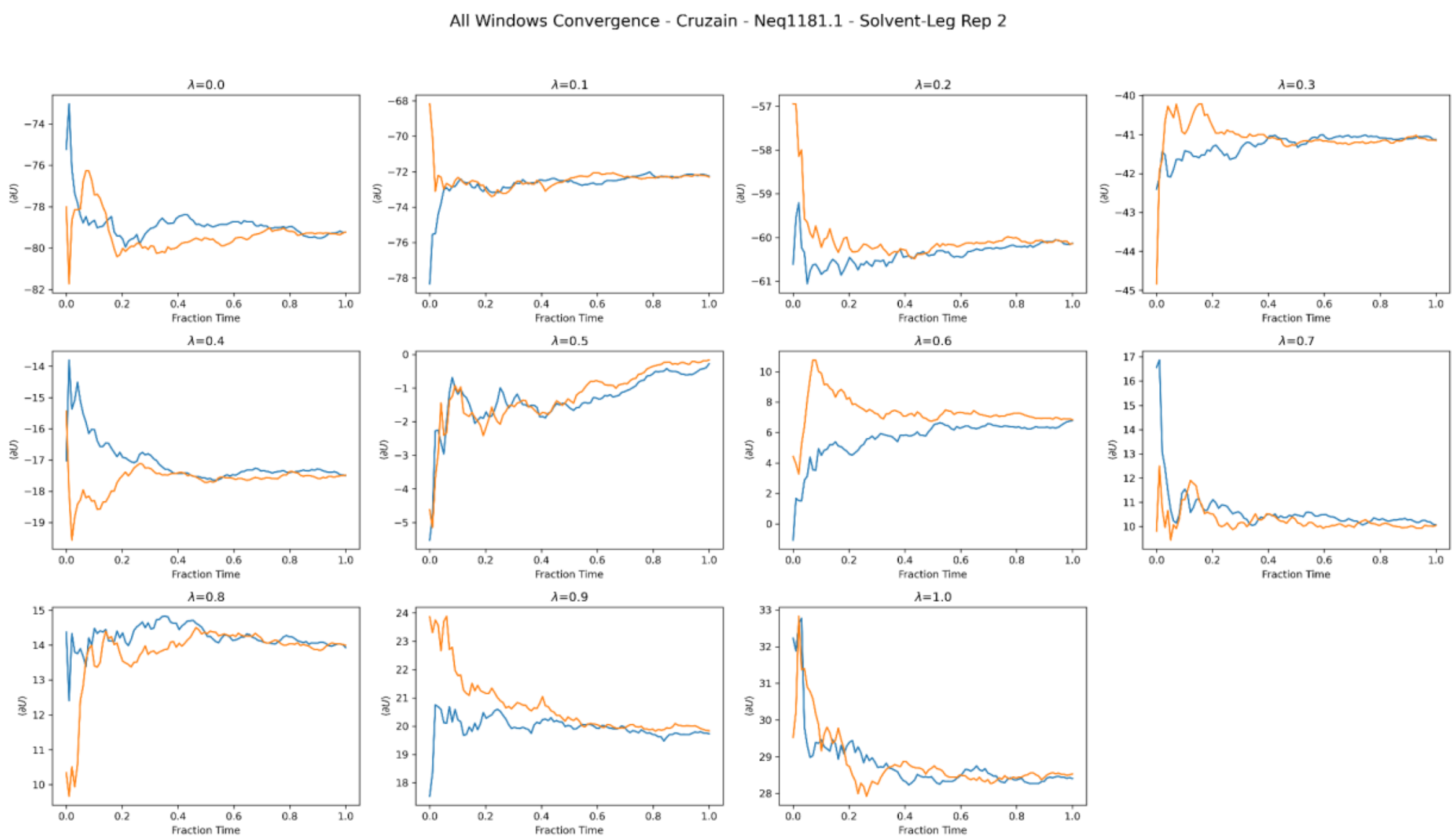

Figure S22. Temporal convergence profile of ⟨∂U/∂λ⟩ across all simulated λ windows in the solvent step for the alchemical transformation from cruzain–Neq1181.1 to cruzain–Neq1182.1, (replicate 2).

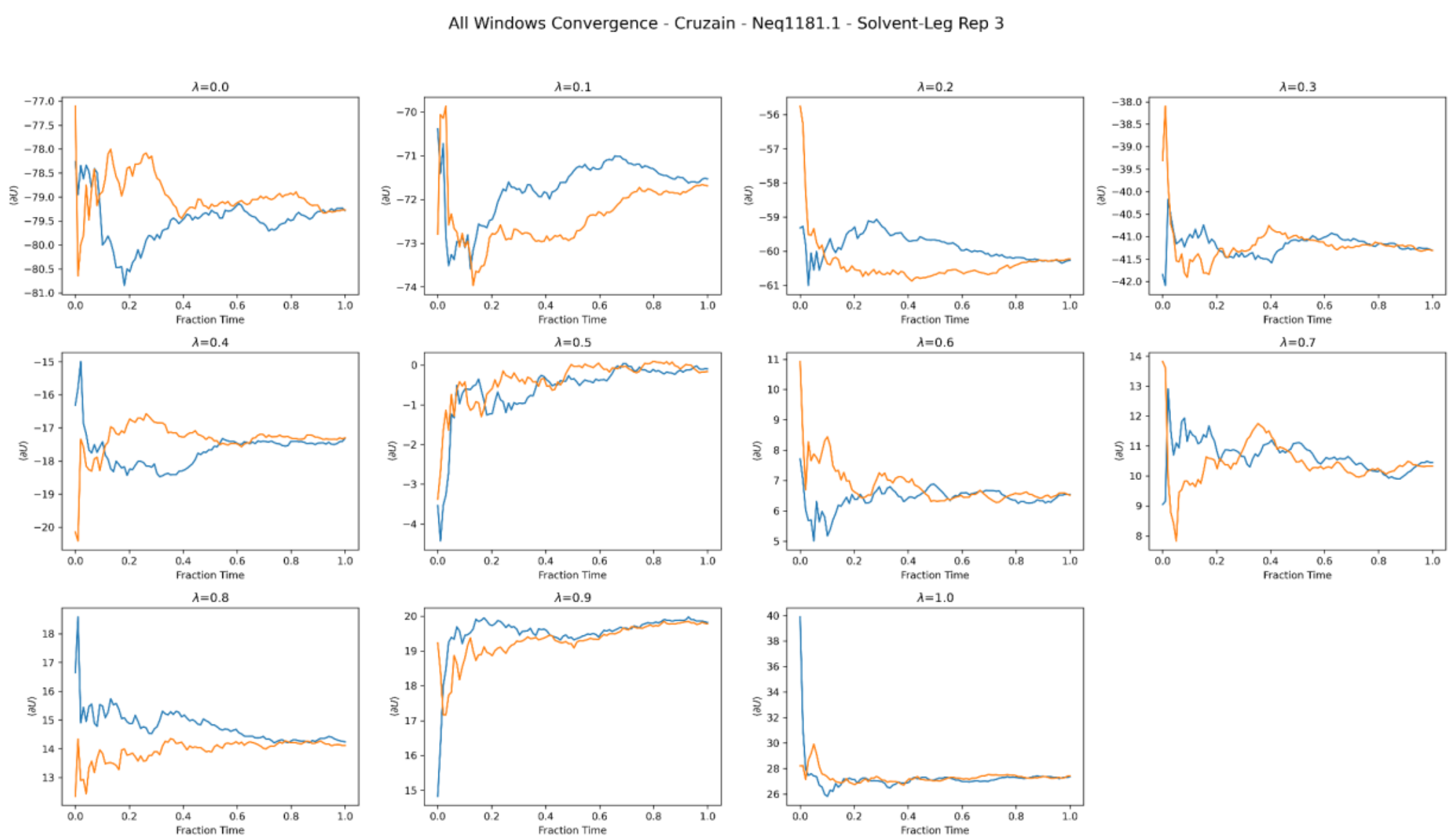

Figure S23. Temporal convergence profile of ⟨∂U/∂λ⟩ across all simulated λ windows in the solvent step for the alchemical transformation from cruzain–Neq1181.1 to cruzain–Neq1182.1, (replicate 3).

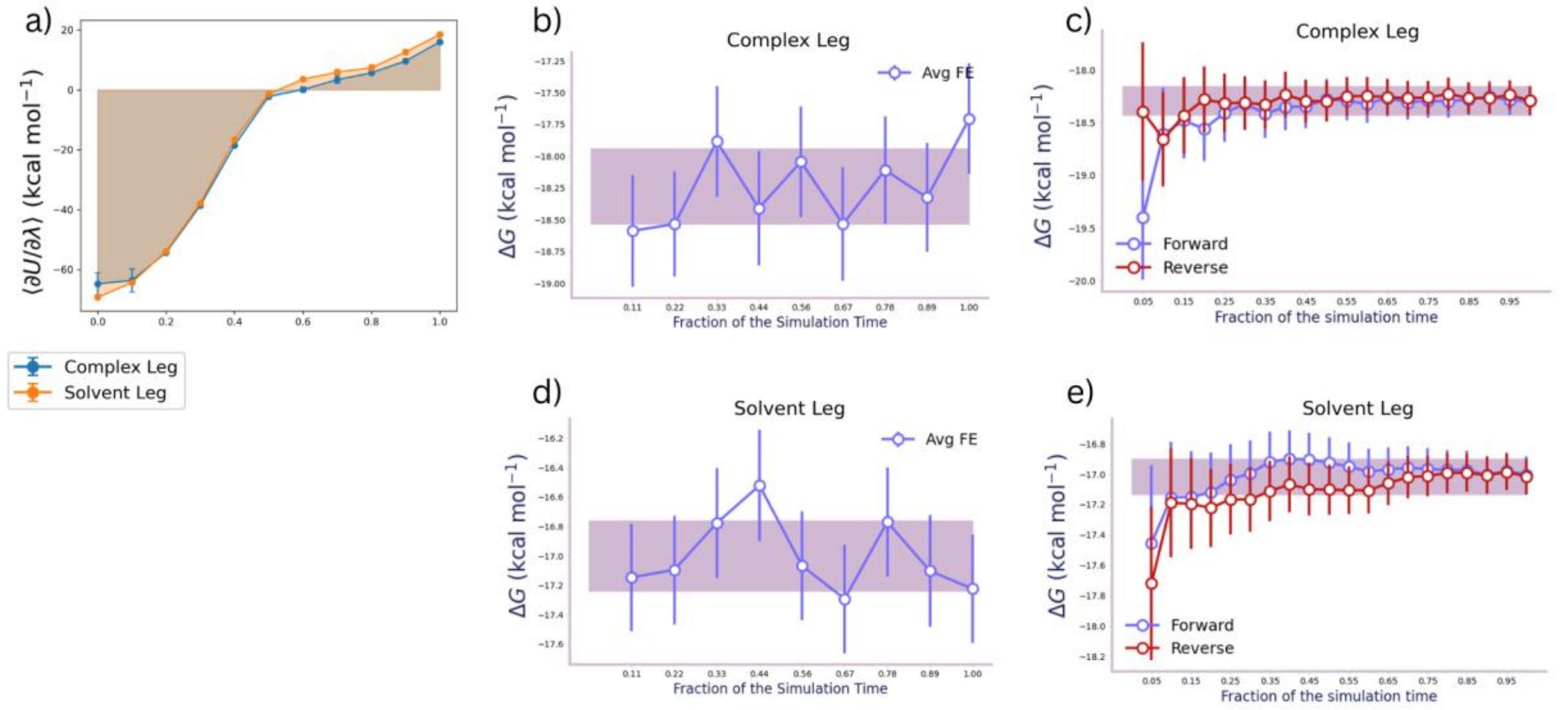

Figure S24. Free energy convergence analysis for the alchemical transformation from cruzain-Neq1268) as a function of the coupling parameter λ for both the complex and solvent phases. (b) Block average convergence analysis and (c) Forward-Backward bidirectional convergence estimate of the free energy variation (ΔG) for the complex phase. (d) Block average convergence analysis and (e) Forward-Backward bidirectional convergence estimate of ΔG for the solvent leg.

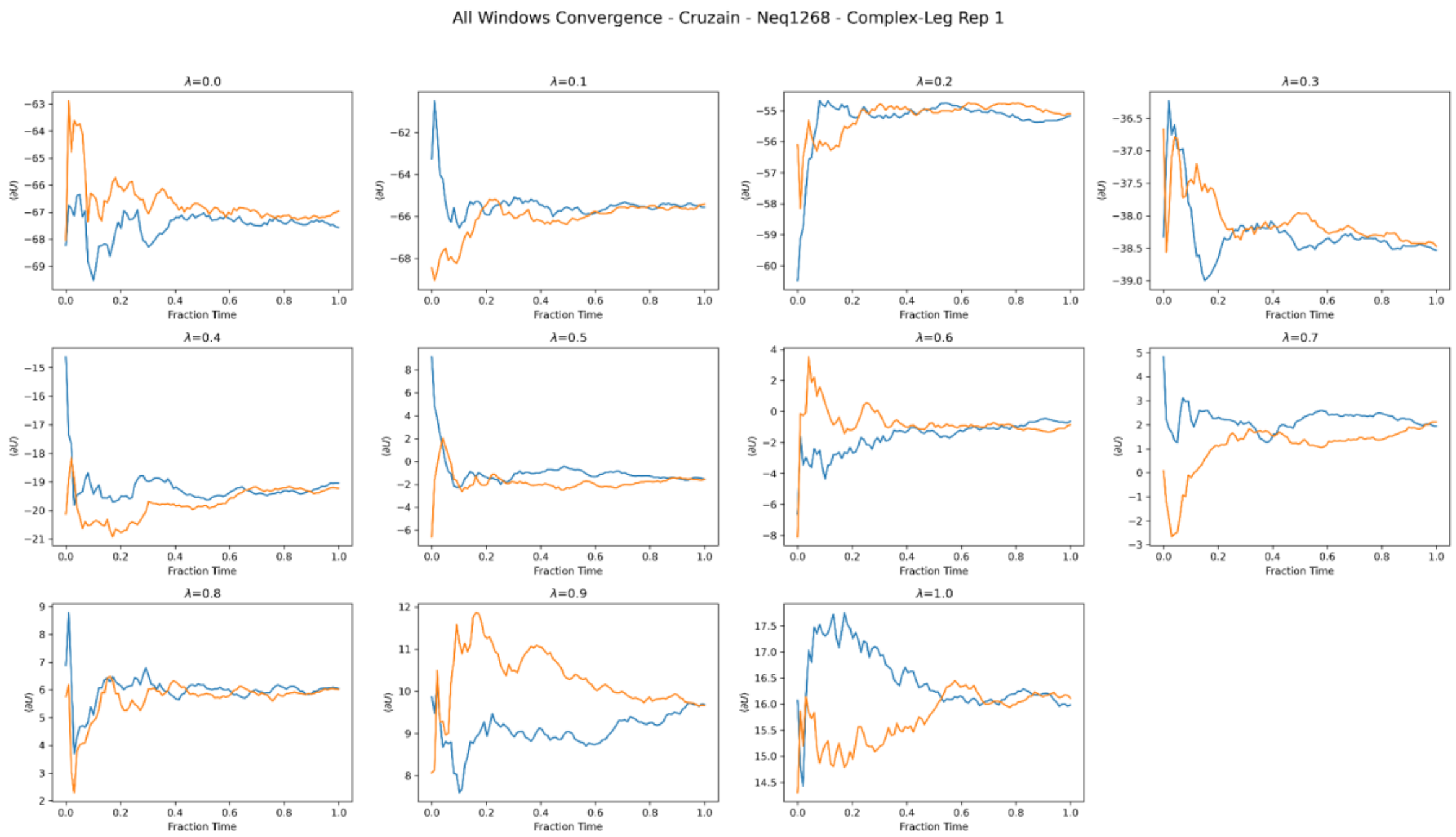

Figure S25. Temporal convergence profile of ⟨∂U/∂λ⟩ across all simulated λ windows in the complex step for the alchemical transformation from cruzain-Neq1268 to cruzain-Neq1276, (replicate 1).

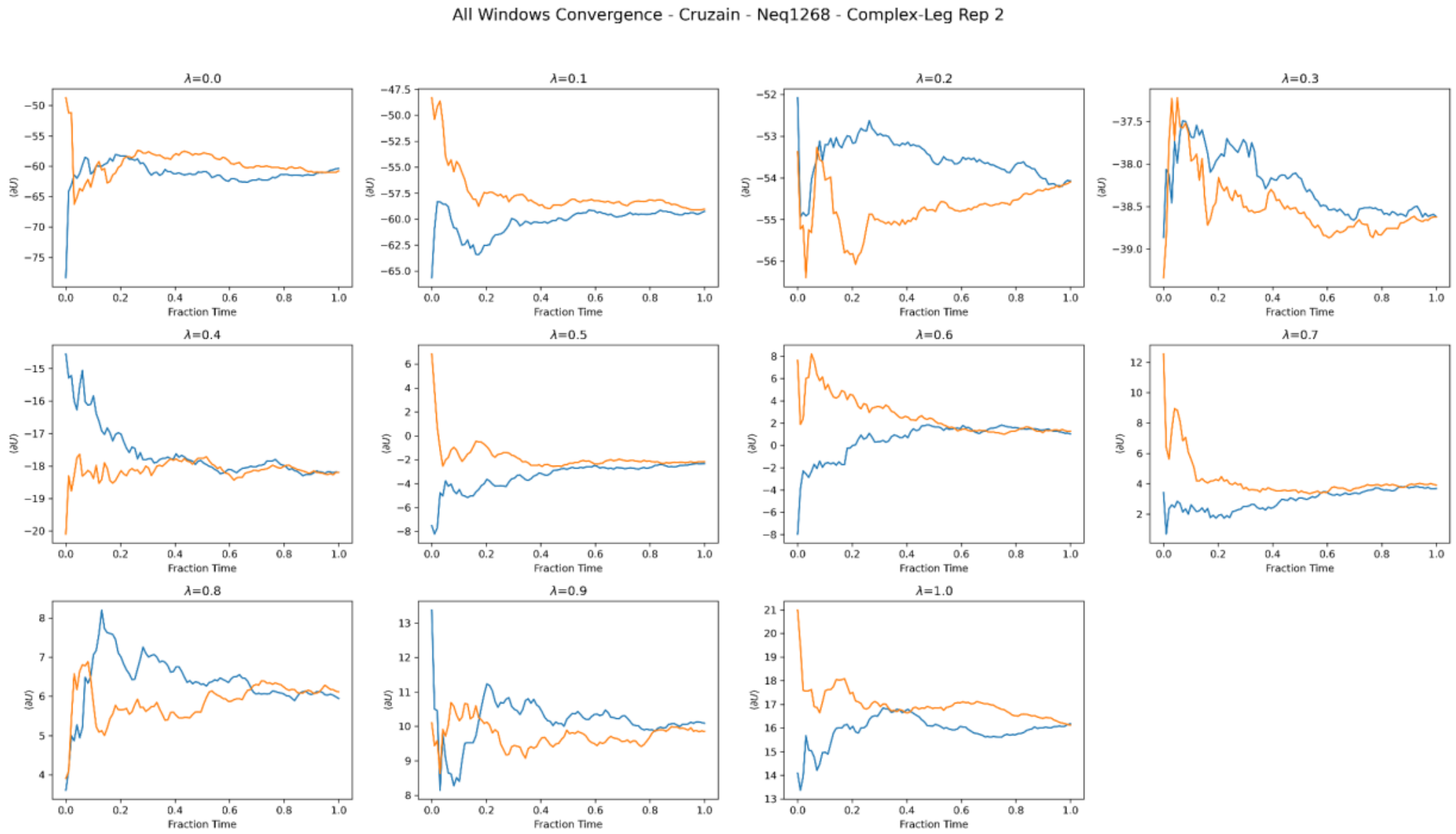

Figure S26. Temporal convergence profile of ⟨∂U/∂λ⟩ across all simulated λ windows in the complex step for the alchemical transformation from cruzain-Neq1268 to cruzain-Neq1276, (replicate 2).

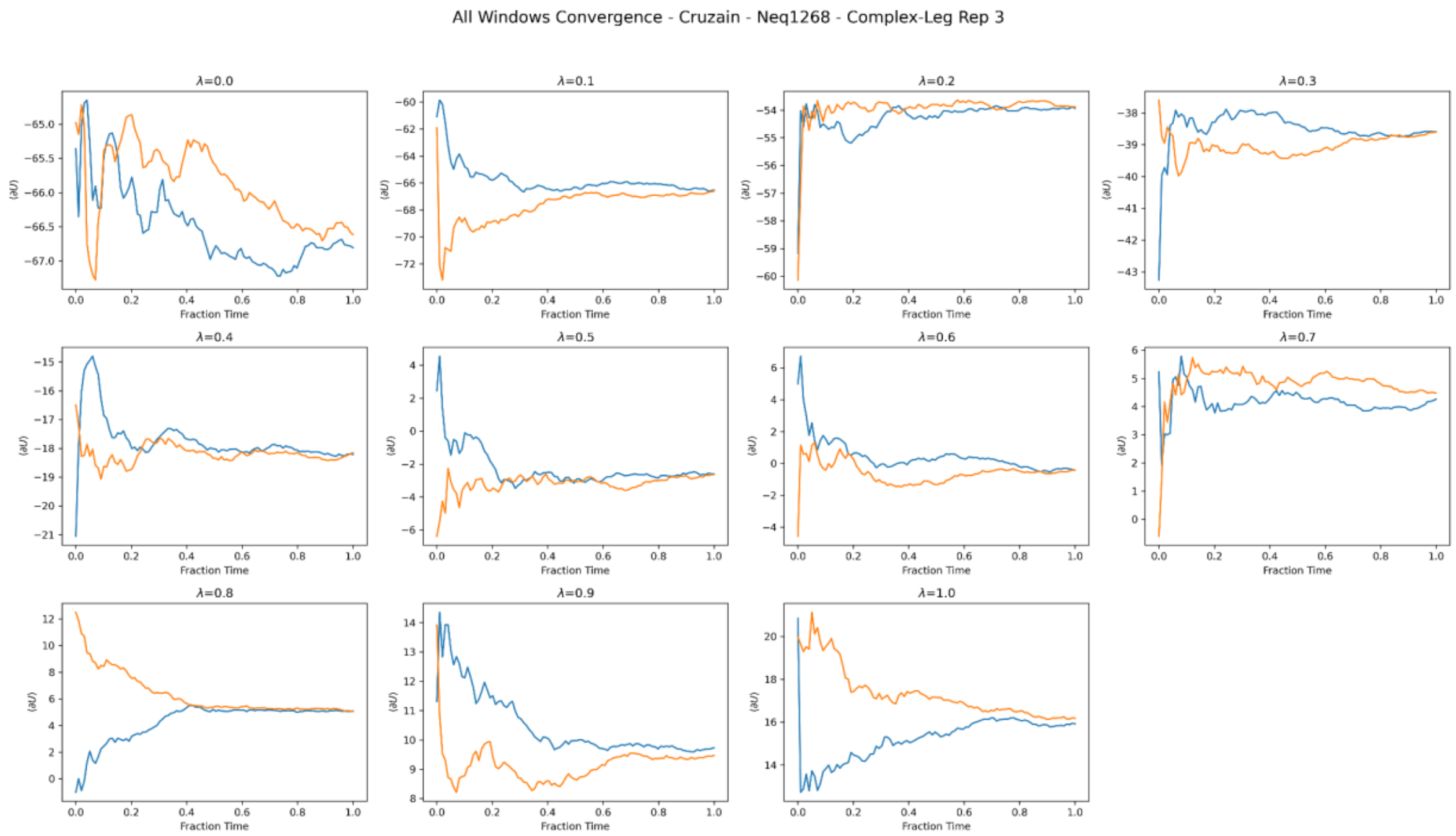

Figure S27. Temporal convergence profile of ⟨∂U/∂λ⟩ across all simulated λ windows in the complex step for the alchemical transformation from cruzain-Neq1268 to cruzain-Neq1276, (replicate 3).

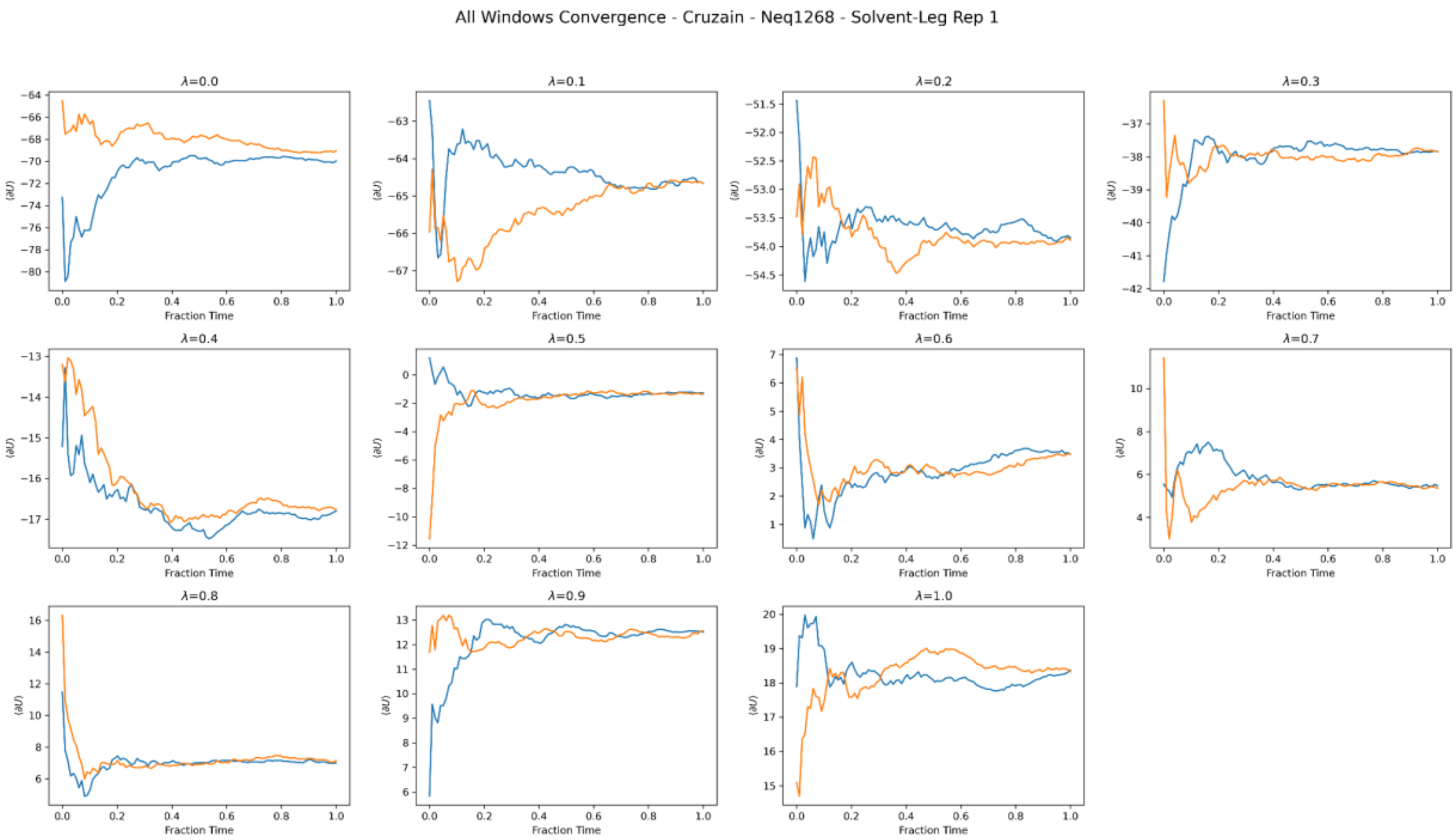

Figure S28. Temporal convergence profile of ⟨∂U/∂λ⟩ across all simulated λ windows in the solvent step for the alchemical transformation from cruzain–Neq1268 to cruzain–Neq1276, (replicate 1).

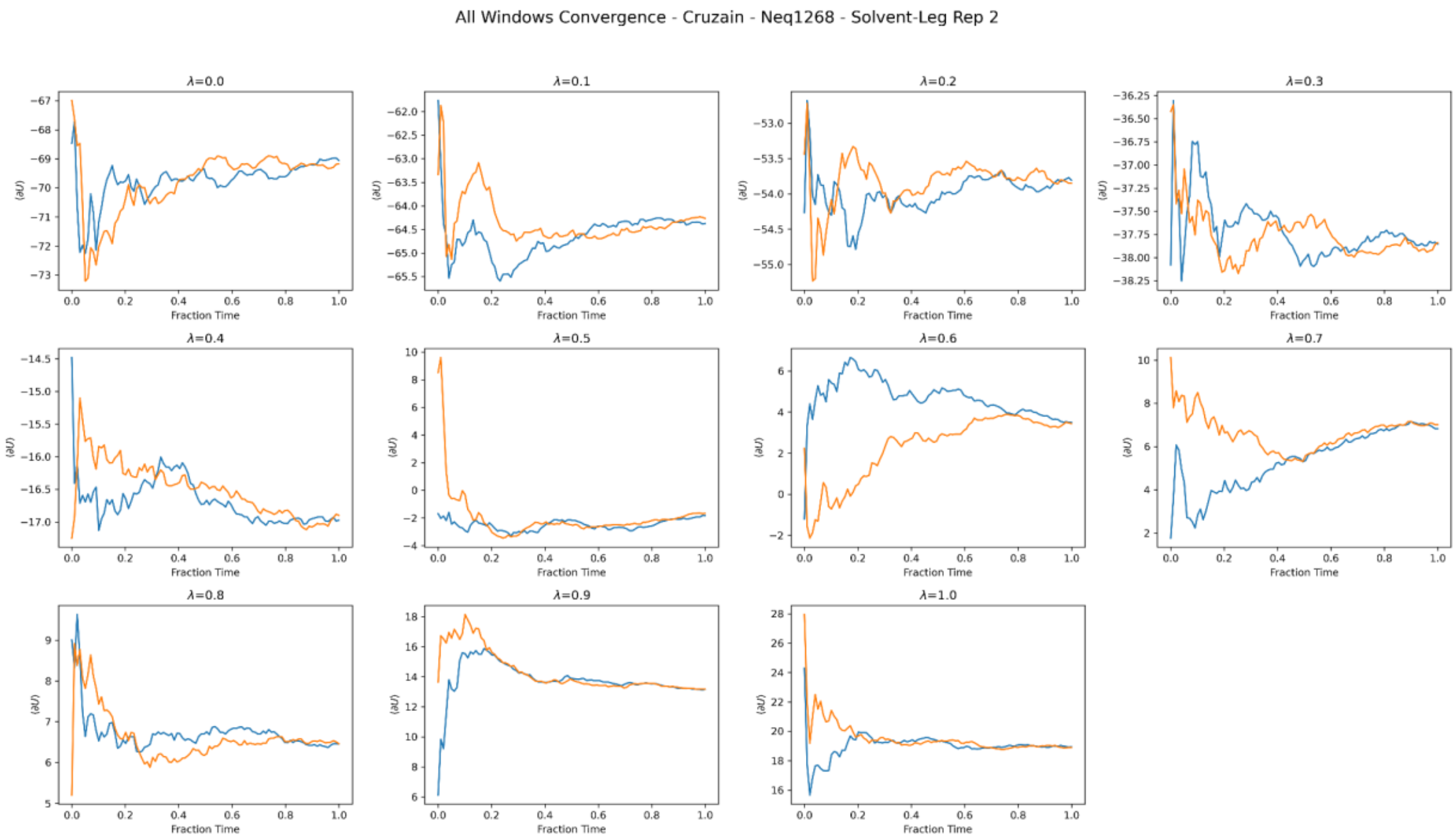

Figure S29. Temporal convergence profile of ⟨∂U/∂λ⟩ across all simulated λ windows in the solvent step for the alchemical transformation from cruzain–Neq1268 to cruzain–Neq1276, (replicate 2).

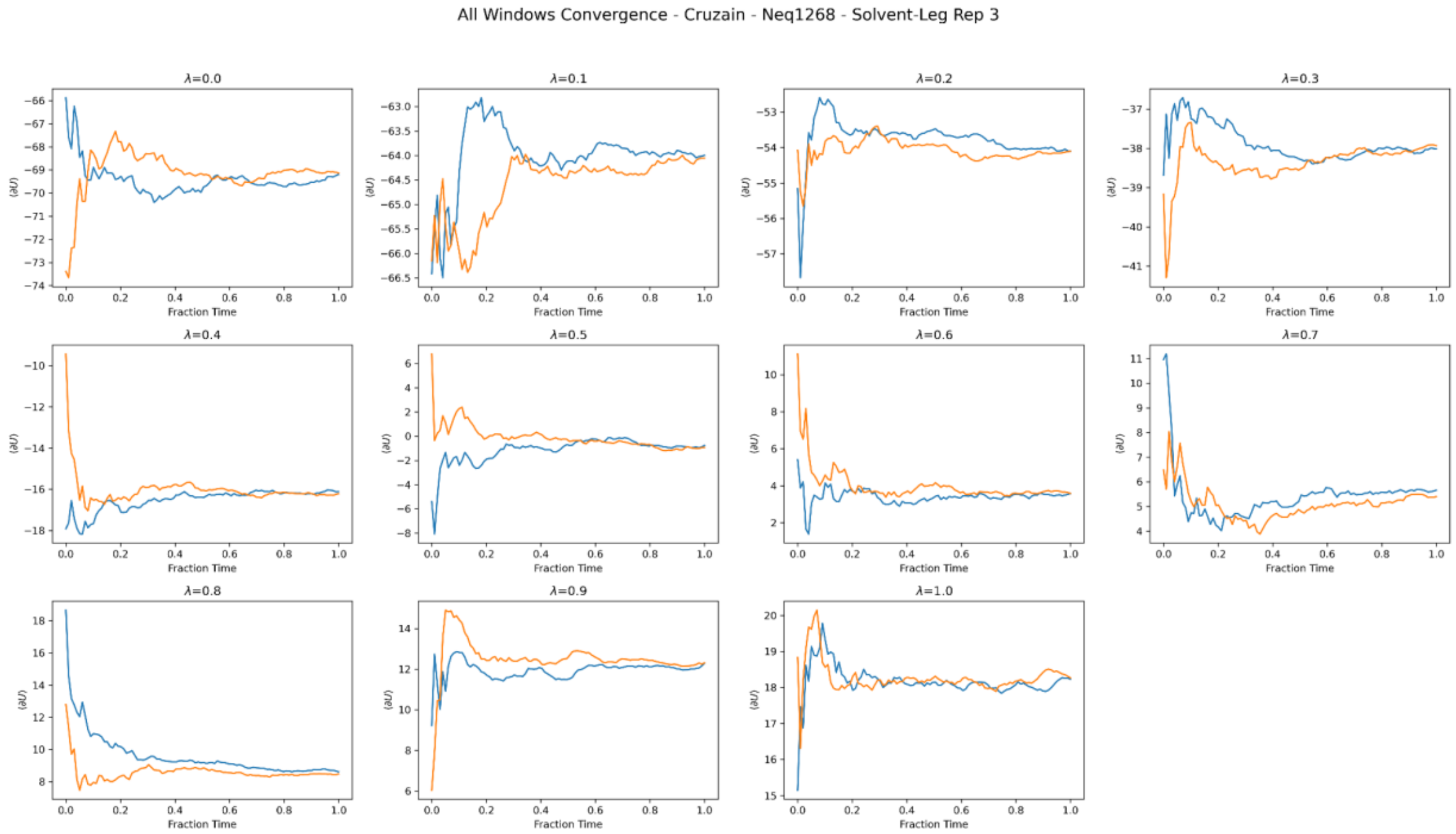

Figure S30. Temporal convergence profile of ⟨∂U/∂λ⟩ across all simulated λ windows in the solvent step for the alchemical transformation from cruzain-Neq1268 to cruzain-Neq1276, (replicate 3).

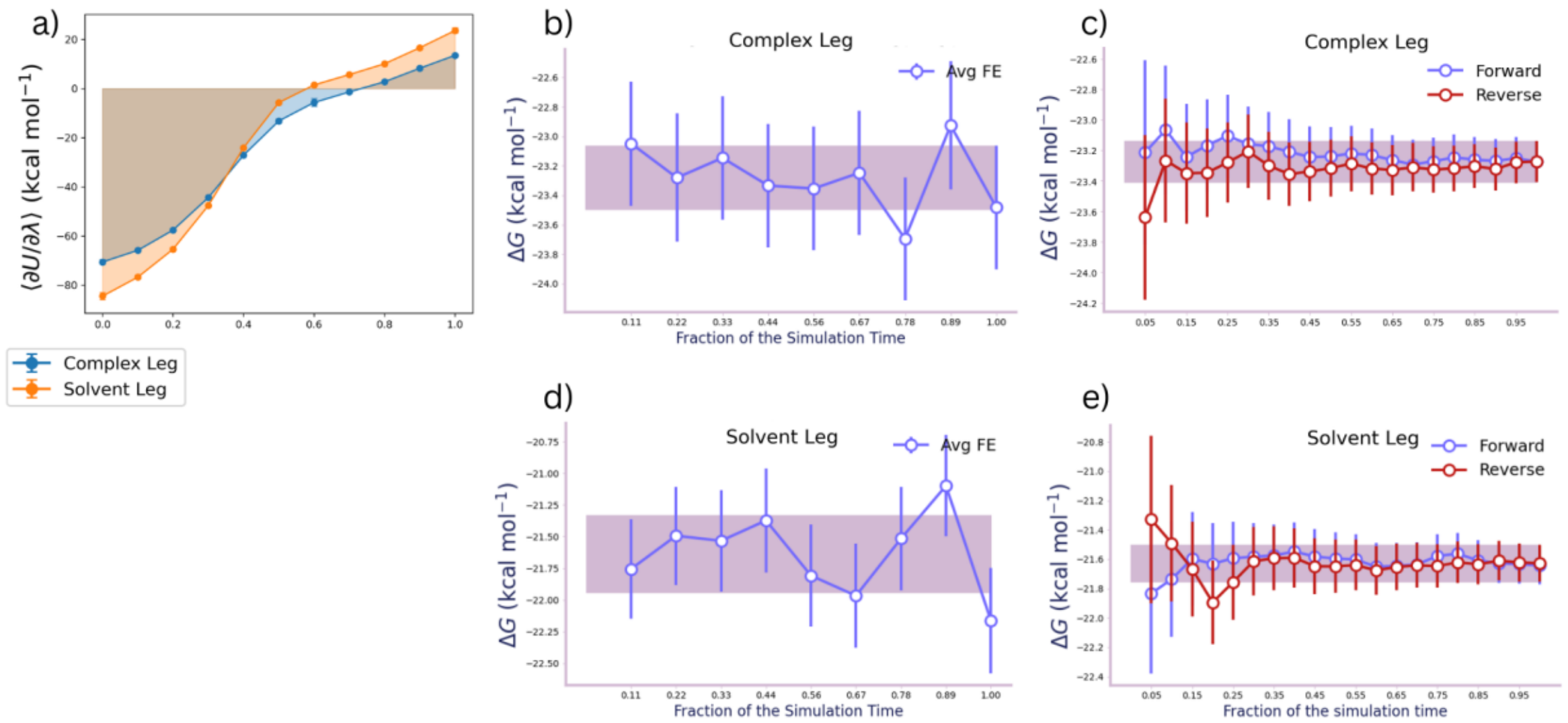

Figure S31. Free energy convergence analysis for the alchemical transformation from hCatL-Neq1172.1) as a function of the coupling parameter $\boldsymbol{\lambda}$ for both the complex and solvent phases. (b) Block average convergence analysis and (c) Forward-Backward bidirectional convergence estimate of the free energy variation (ΔG) for the complex phase. (d) Block average convergence analysis and (e) Forward-Backward bidirectional convergence estimate of ΔG for the solvent leg.

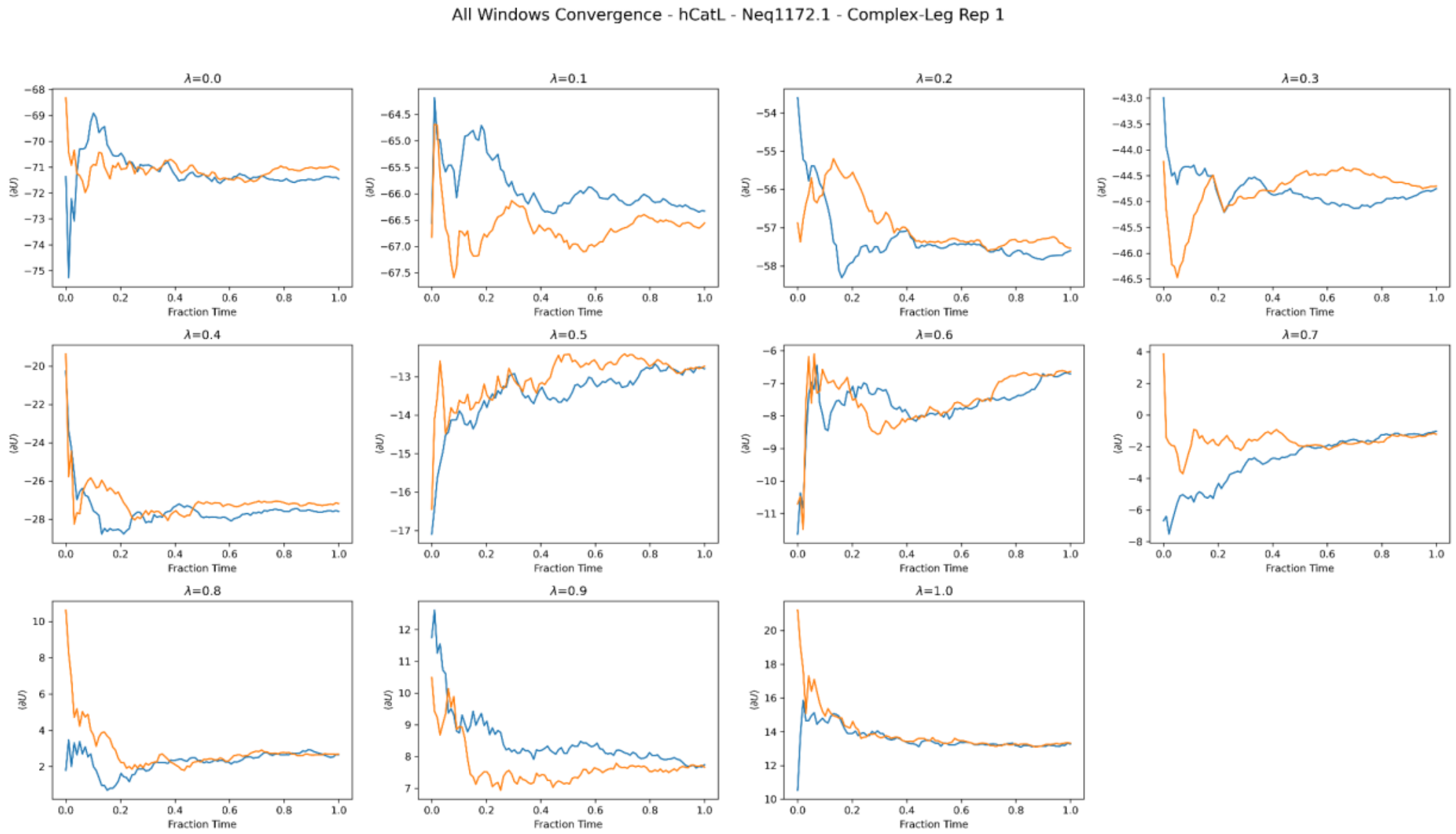

Figure S32. Temporal convergence profile of ⟨∂U/∂λ⟩ across all simulated λ windows in the complex step for the alchemical transformation from hCatL-Neq1172.1 to hCatL-Neq1171.1, (replicate 1).

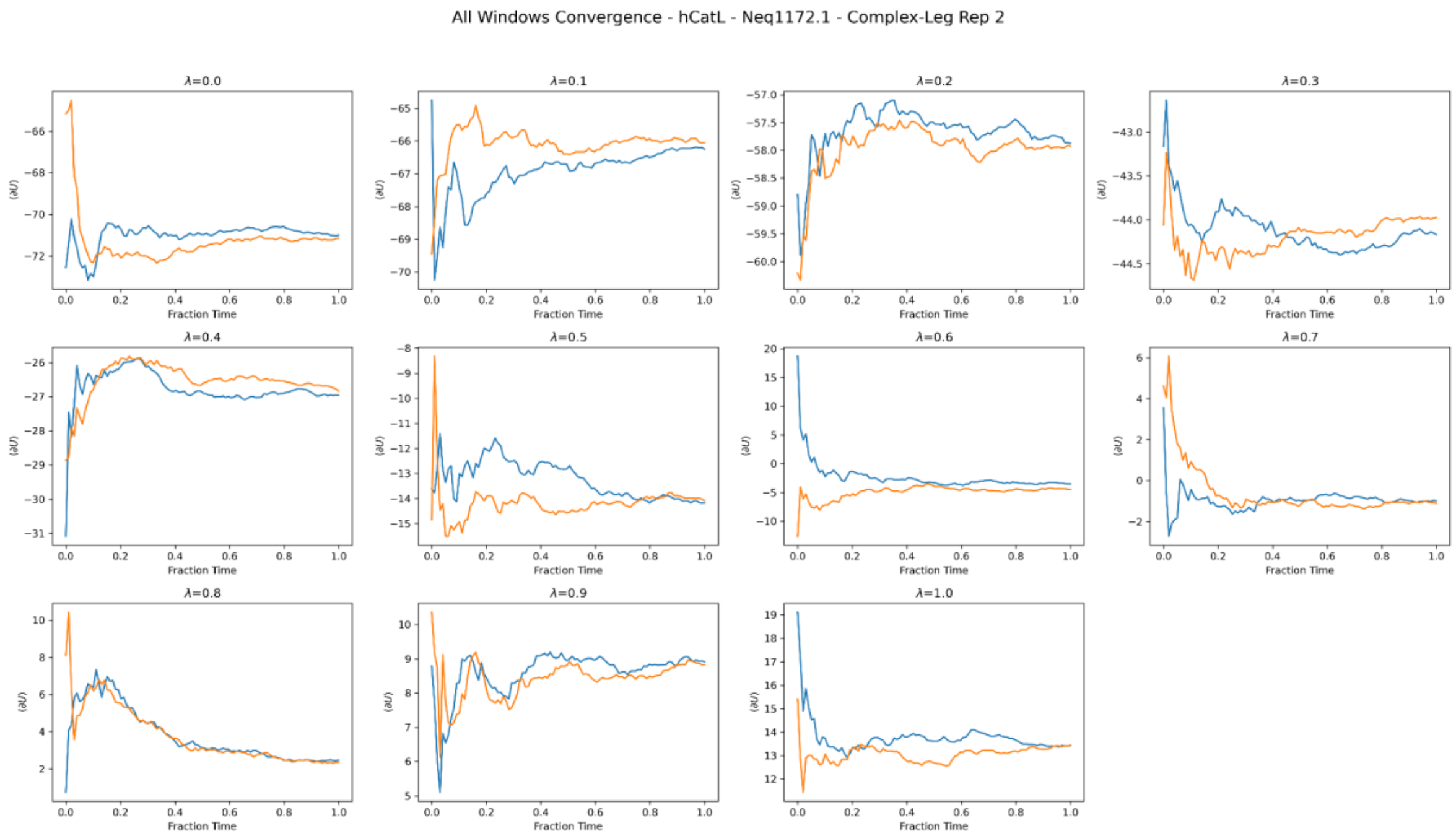

Figure S33. Temporal convergence profile of ⟨∂U/∂λ⟩ across all simulated λ windows in the complex step for the alchemical transformation from hCatL-Neq1172.1 to hCatL-Neq1171.1, (replicate 2).

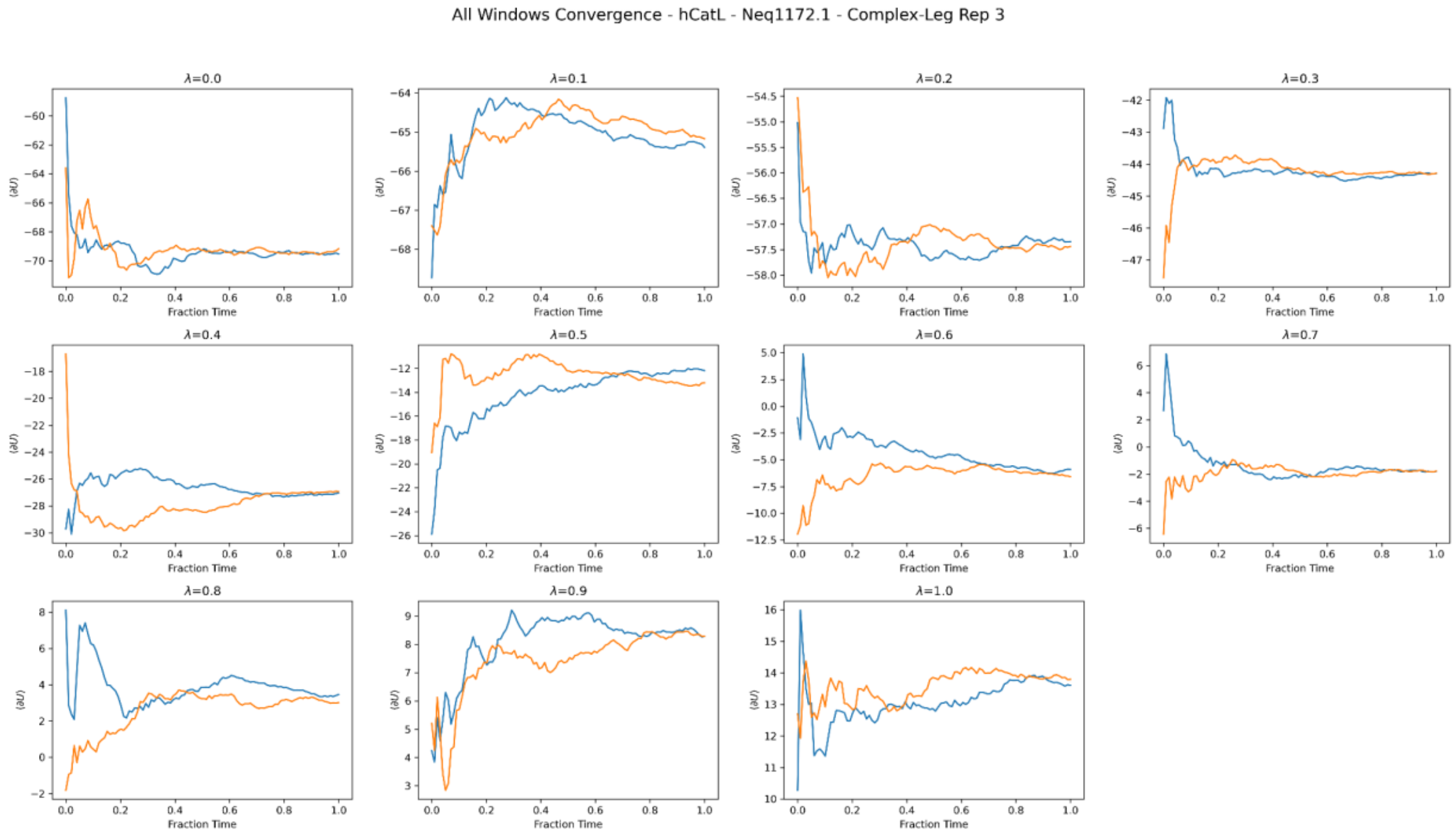

Figure S34. Temporal convergence profile of ⟨∂U/∂λ⟩ across all simulated λ windows in the complex step for the alchemical transformation from hCatL–Neq1172.1 to hCatL–Neq1171.1, (replicate 3).

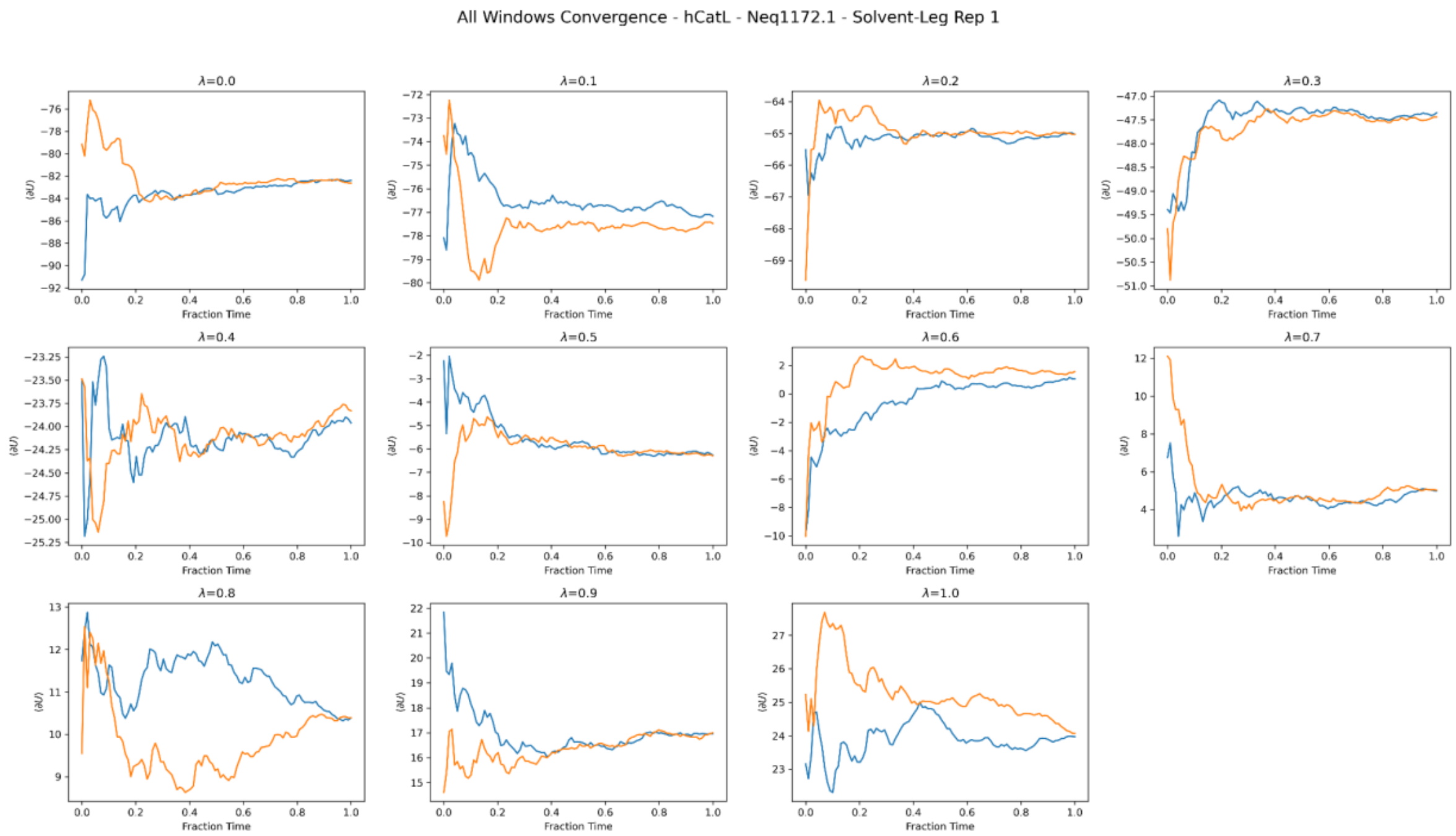

Figure S35. Temporal convergence profile of ⟨∂U/∂λ⟩ across all simulated λ windows in the solvent step for the alchemical transformation from hCatL–Neq1172.1 to hCatL–Neq1171.1, (replicate 1).

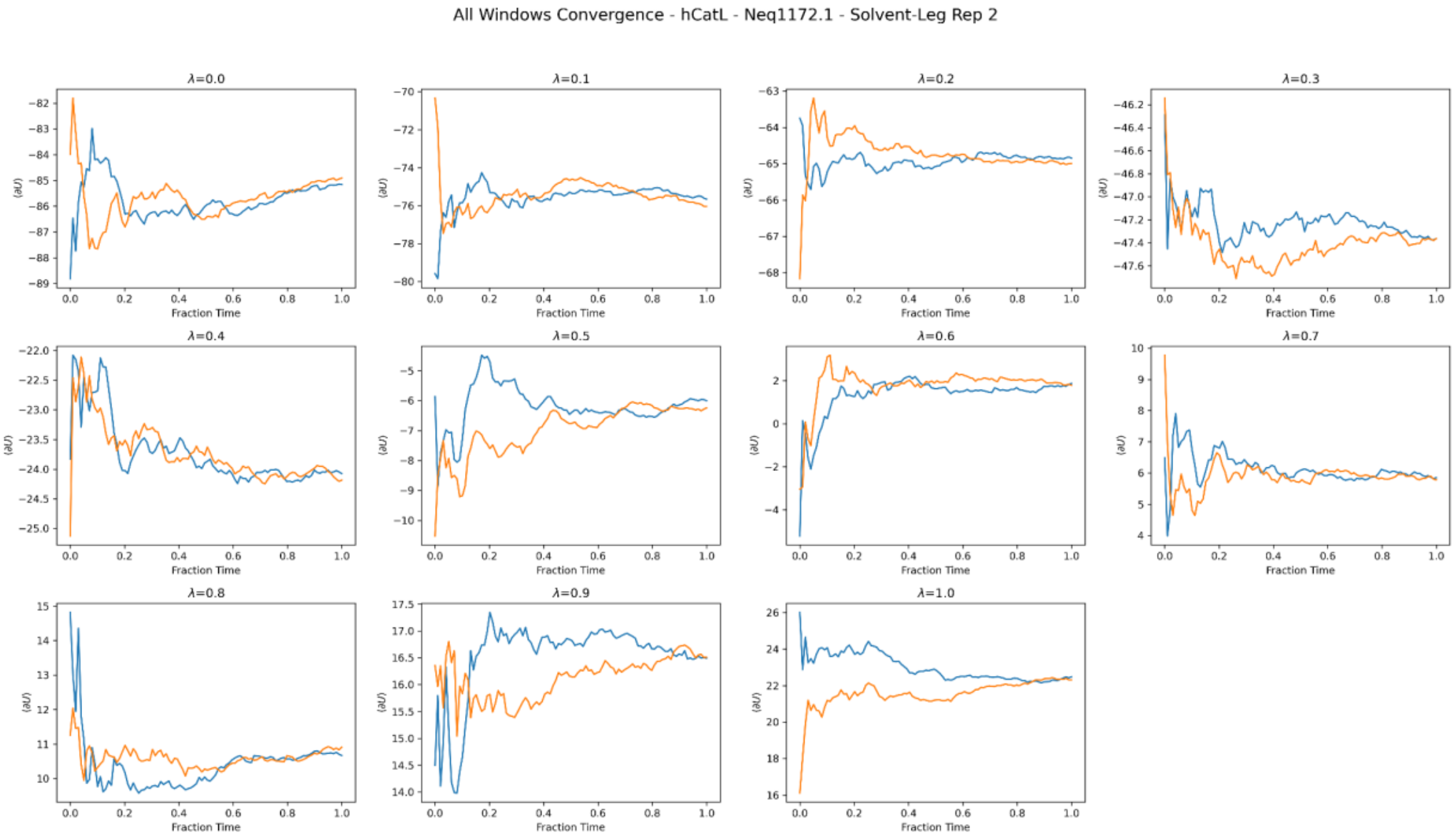

Figure S36. Temporal convergence profile of ⟨∂U/∂λ⟩ across all simulated λ windows in the solvent step for the alchemical transformation from hCatL–Neq1172.1 to hCatL–Neq1171.1, (replicate 2).

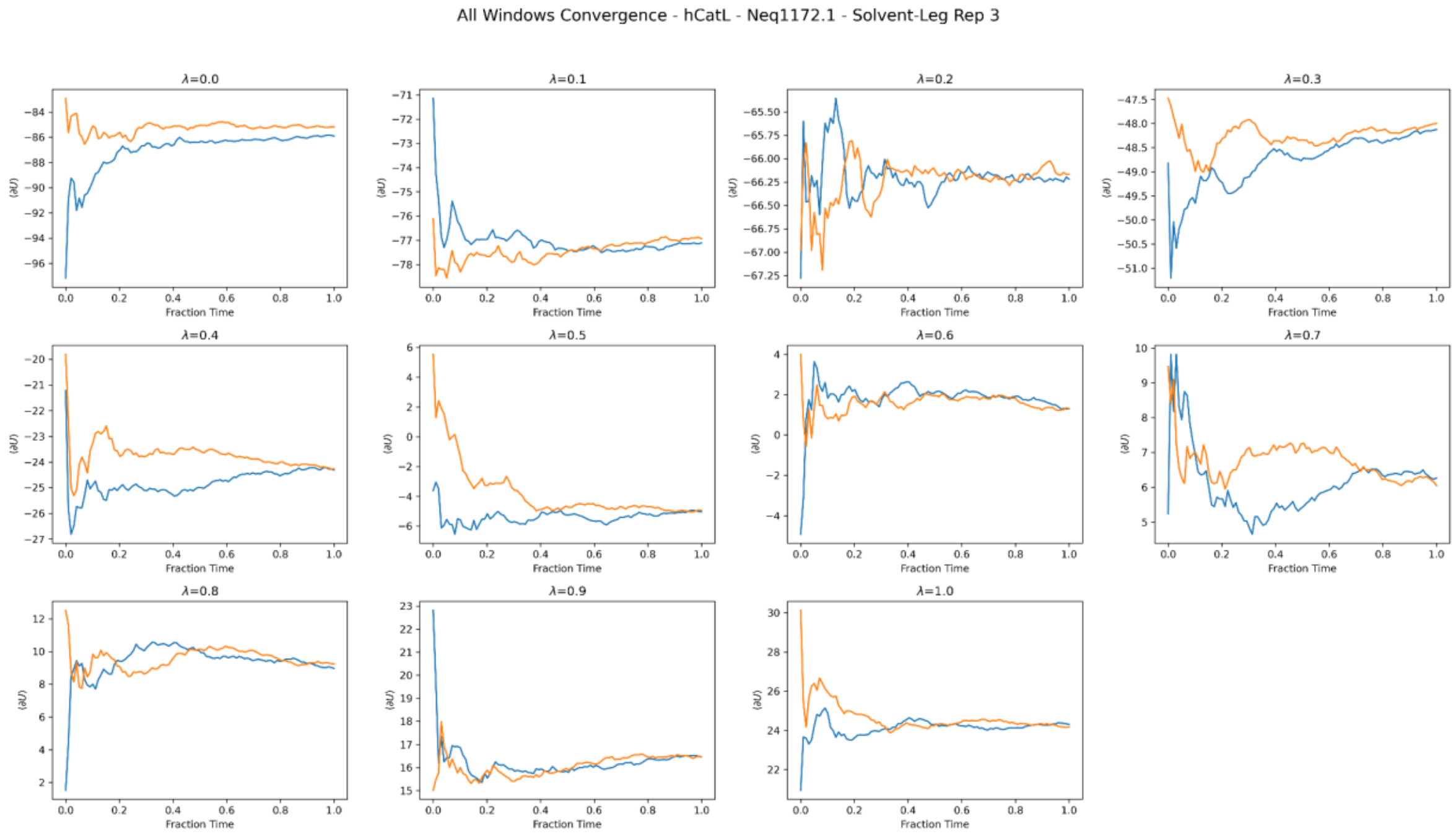

Figure S37. Temporal convergence profile of ⟨∂U/∂λ⟩ across all simulated λ windows in the solvent step for the alchemical transformation from hCatL–Neq1172.1 to hCatL–Neq1171.1, (replicate 3).

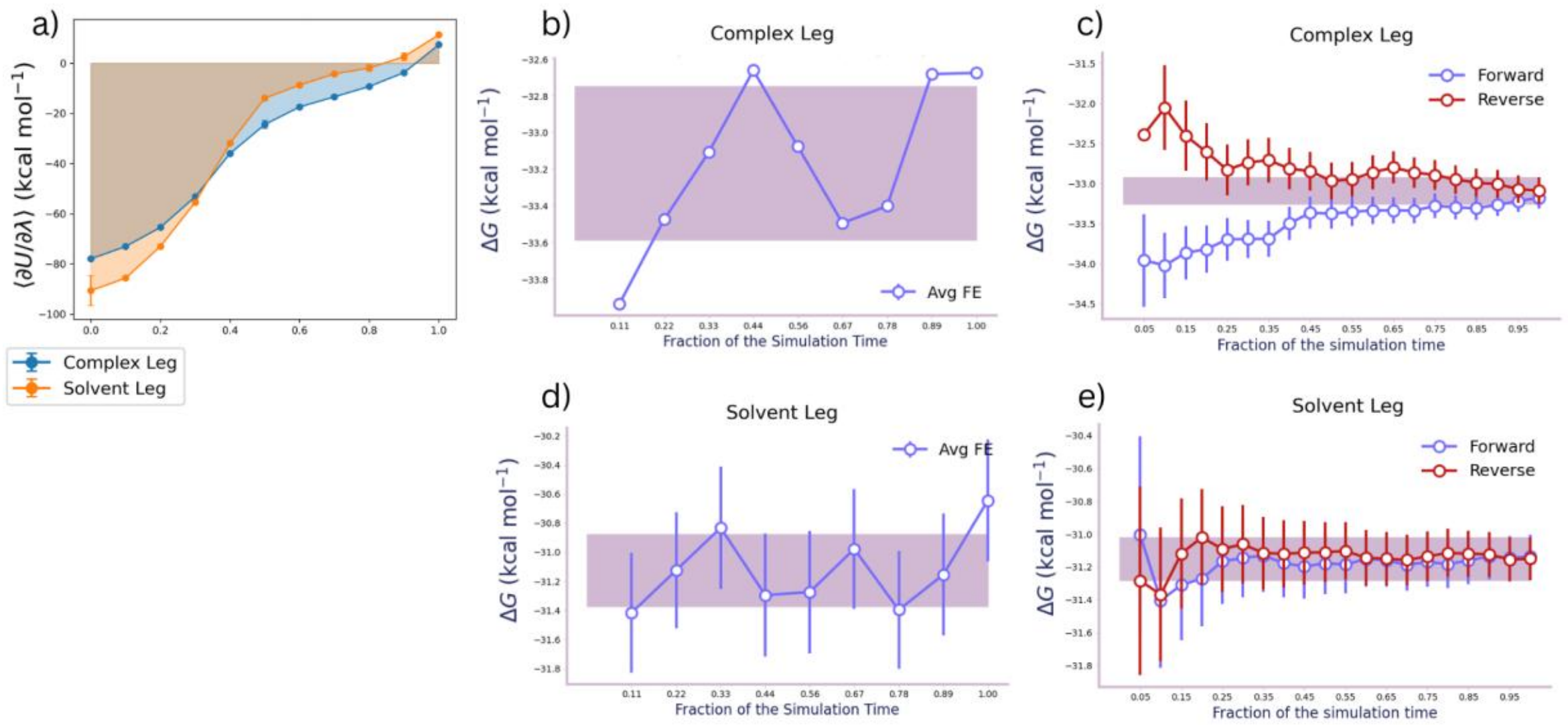

Figure S38. Free energy convergence analysis for the alchemical transformation from hCatL-Neq1181.1) as a function of the coupling parameter **λ** for both the complex and solvent phases. (b) Block average convergence analysis and (c) Forward–Backward bidirectional convergence estimate of the free energy variation (ΔG) for the complex phase. (d) Block average convergence analysis and (e) Forward–Backward bidirectional convergence estimate of ΔG for the solvent leg.

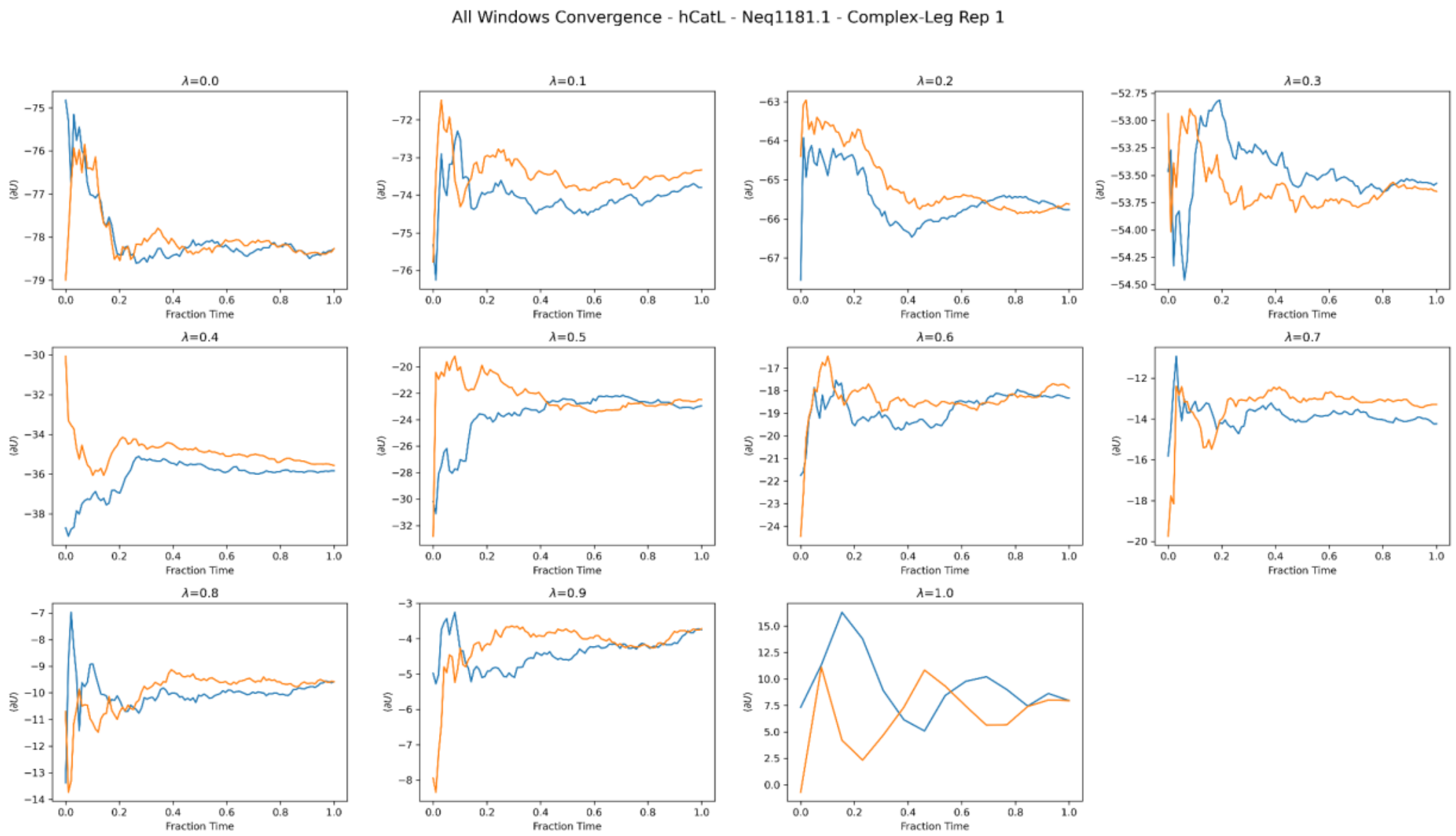

Figure S39. Temporal convergence profile of ⟨∂U/∂λ⟩ across all simulated λ windows in the complex step for the alchemical transformation from hCatL-Neq1181.1 to hCatL-Neq1182.1, (replicate 1).

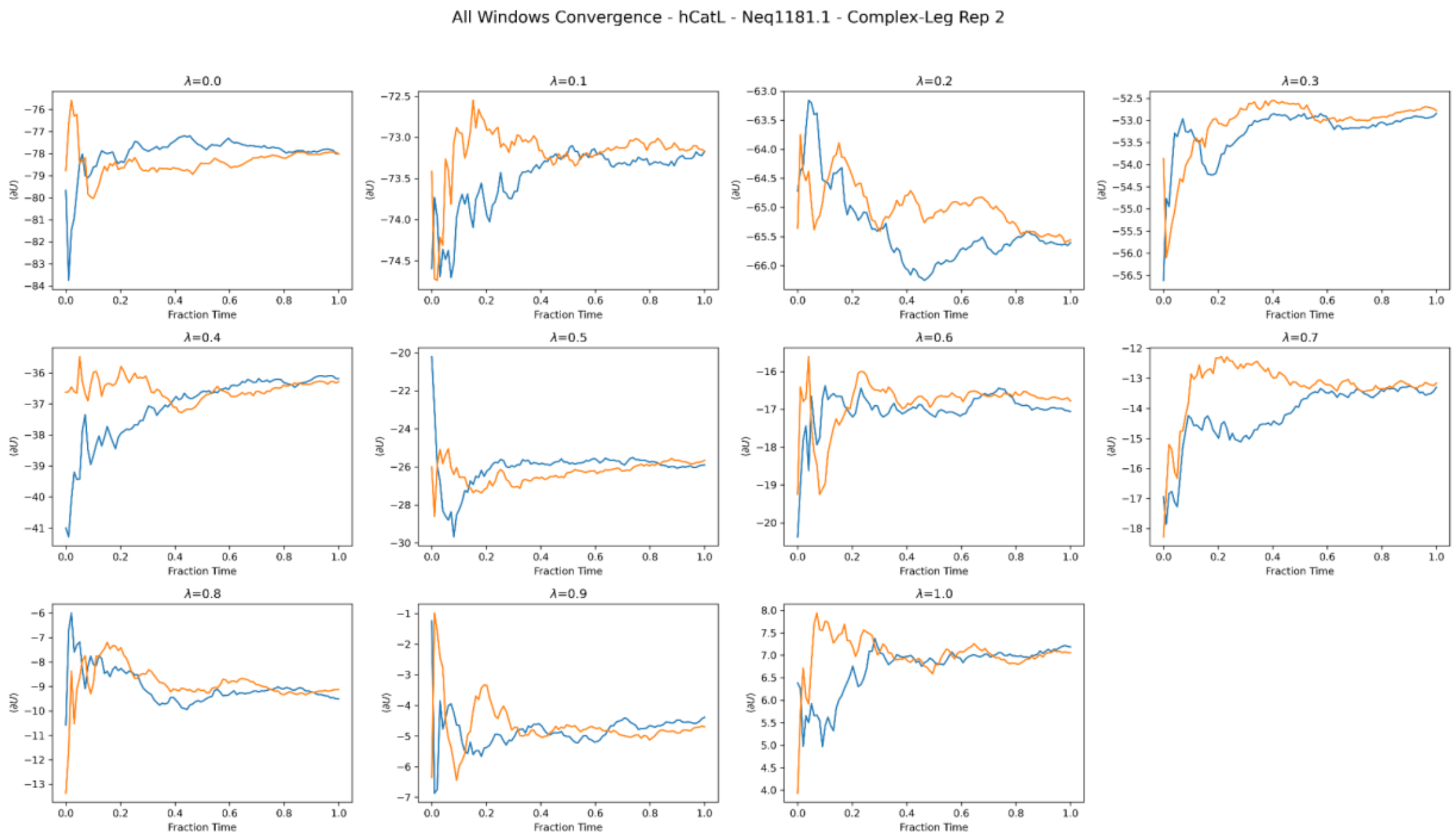

Figure S40. Temporal convergence profile of ⟨∂U/∂λ⟩ across all simulated λ windows in the complex step for the alchemical transformation from hCatL–Neq1181.1 to hCatL–Neq1182.1, (replicate 2).

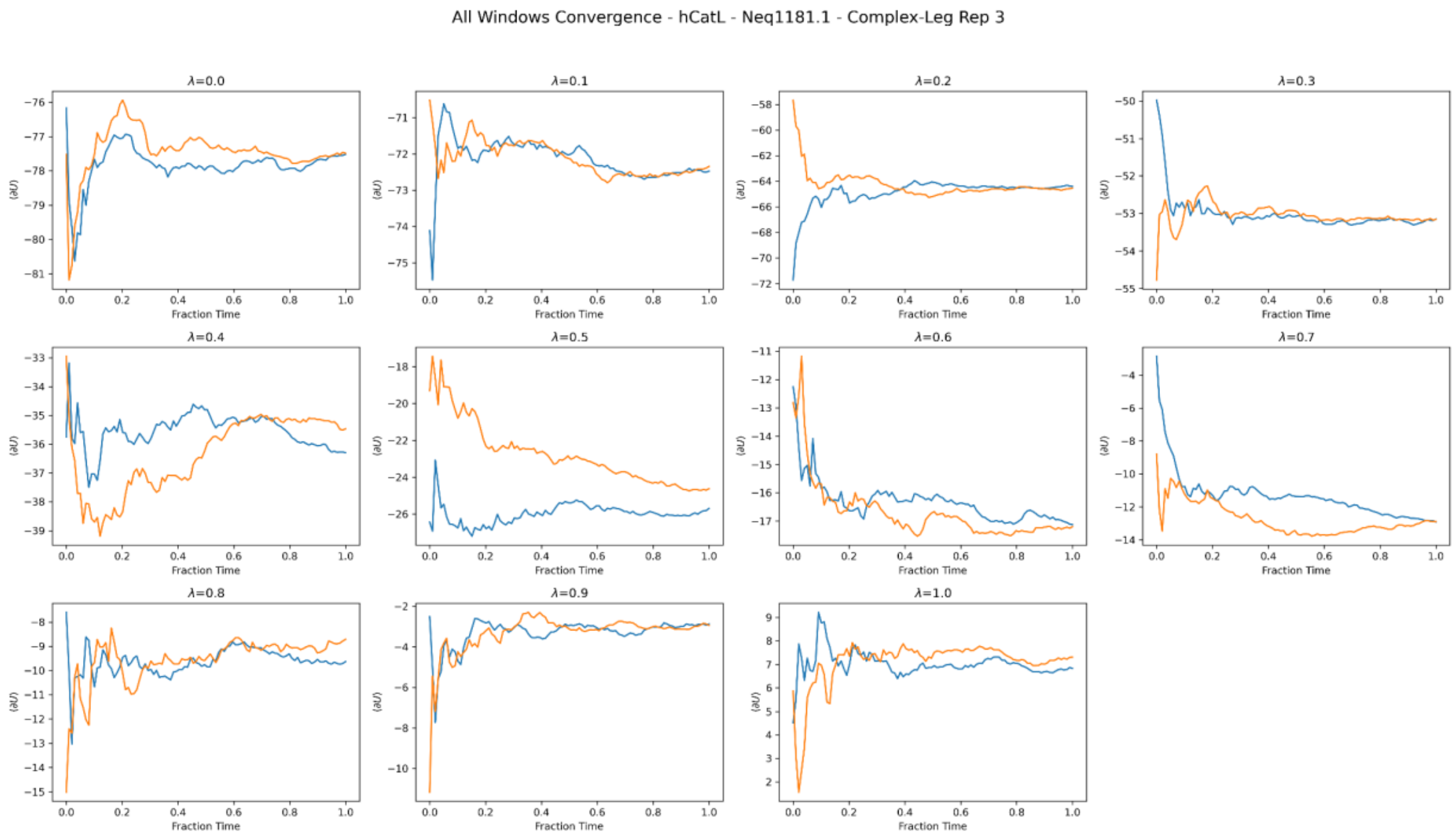

Figure S41. Temporal convergence profile of ⟨∂U/∂λ⟩ across all simulated λ windows in the complex step for the alchemical transformation from hCatL–Neq1181.1 to hCatL–Neq1182.1, (replicate 3).

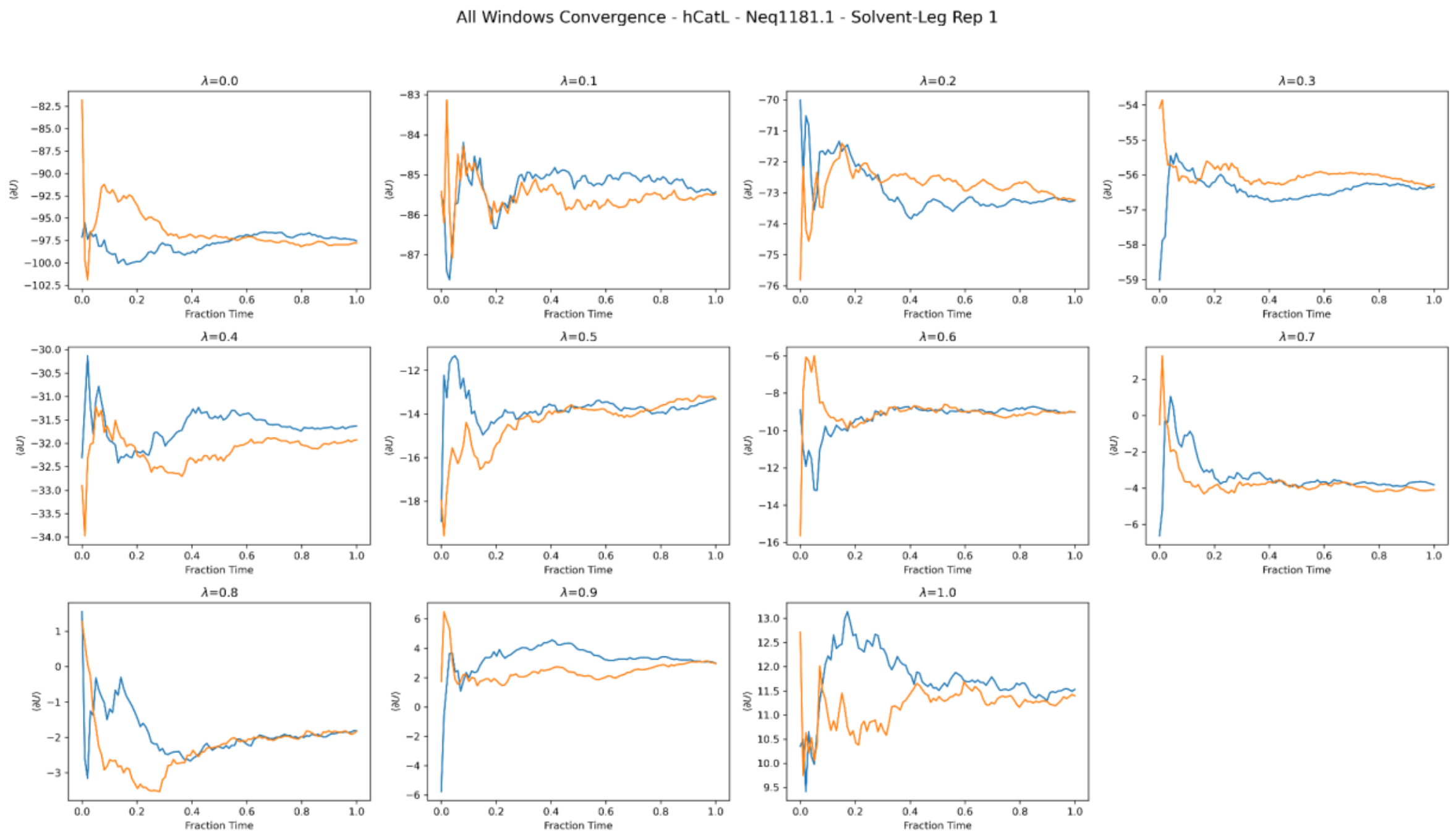

Figure S42. Temporal convergence profile of ⟨∂U/∂λ⟩ across all simulated λ windows in the solvent step for the alchemical transformation from hCatL-Neq1181.1 to hCatL-Neq1182.1, (replicate 1).

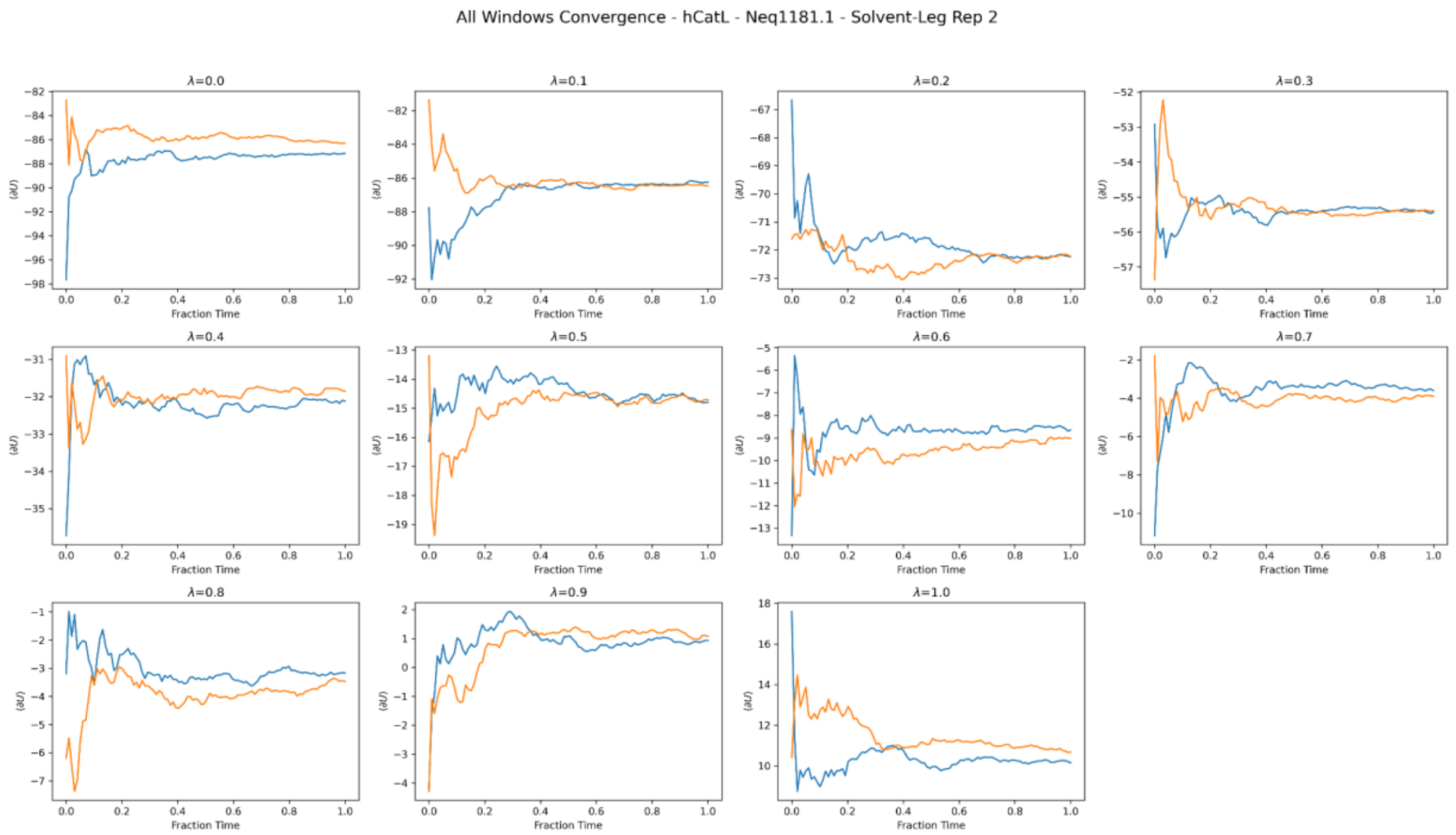

Figure S43. Temporal convergence profile of ⟨∂U/∂λ⟩ across all simulated λ windows in the solvent step for the alchemical transformation from hCatL-Neq1181.1 to hCatL-Neq1182.1, (replicate 2).

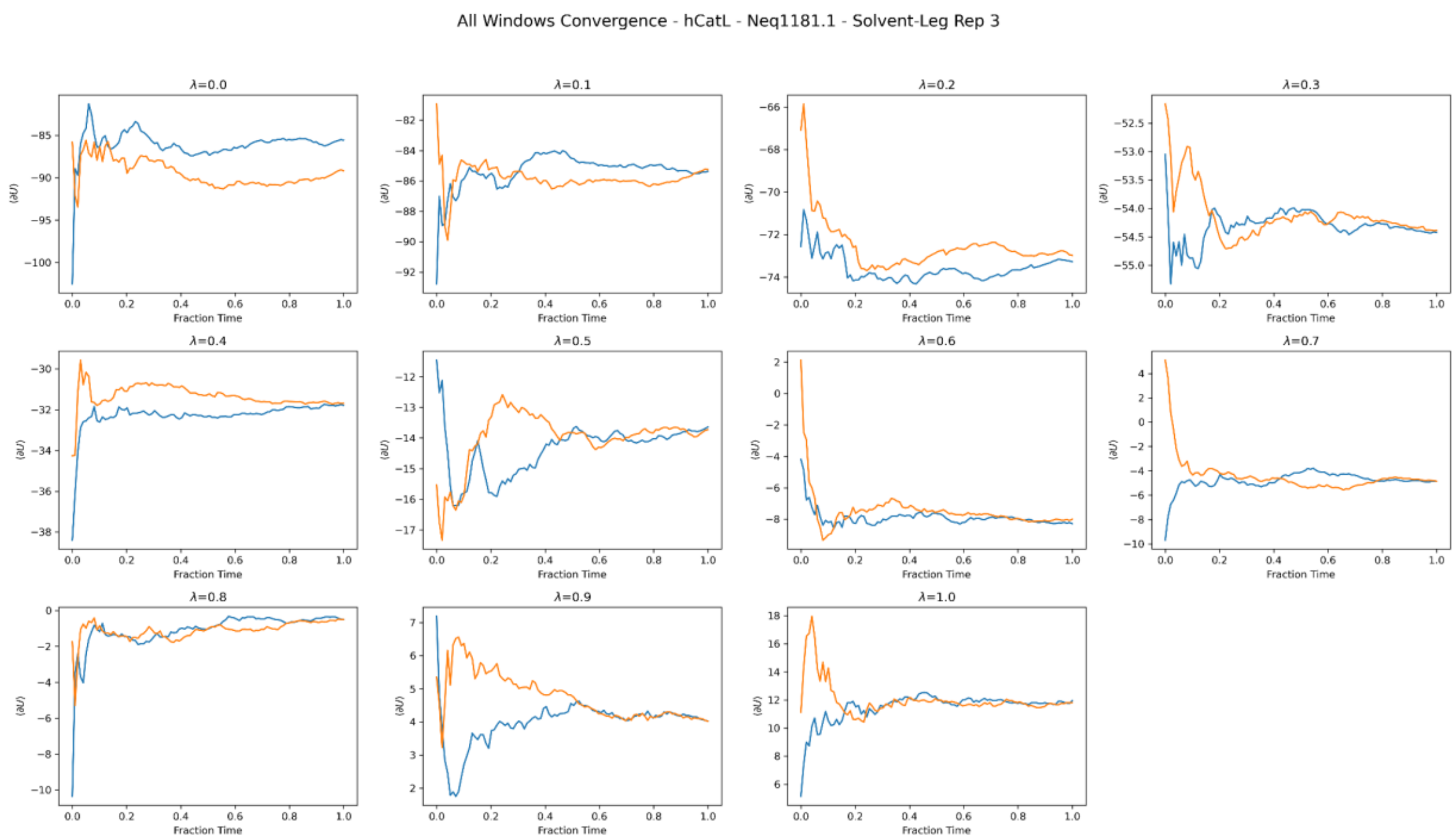

Figure S44. Temporal convergence profile of ⟨∂U/∂λ⟩ across all simulated λ windows in the solvent step for the alchemical transformation from hCatL-Neq1181.1 to hCatL-Neq1182.1, (replicate 3).

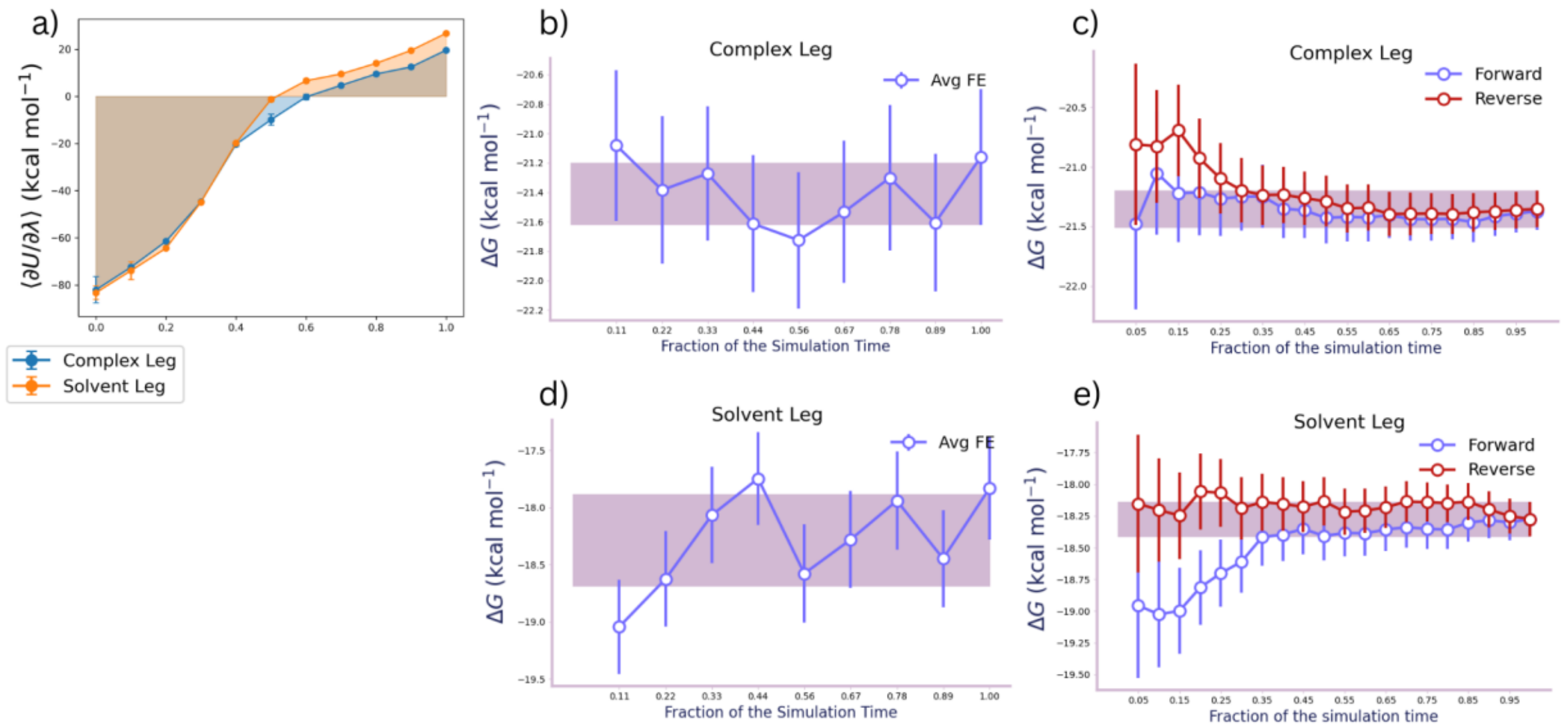

Figure S45. Free energy convergence analysis for the alchemical transformation from hCatL-Neq1268 to) as a function of the coupling parameter λ for both the complex and solvent phases. (b) Block average convergence analysis and (c) Forward–Backward bidirectional convergence estimate of the free energy variation (ΔG) for the complex phase. (d) Block average convergence analysis and (e) Forward–Backward bidirectional convergence estimate of ΔG for the solvent leg.

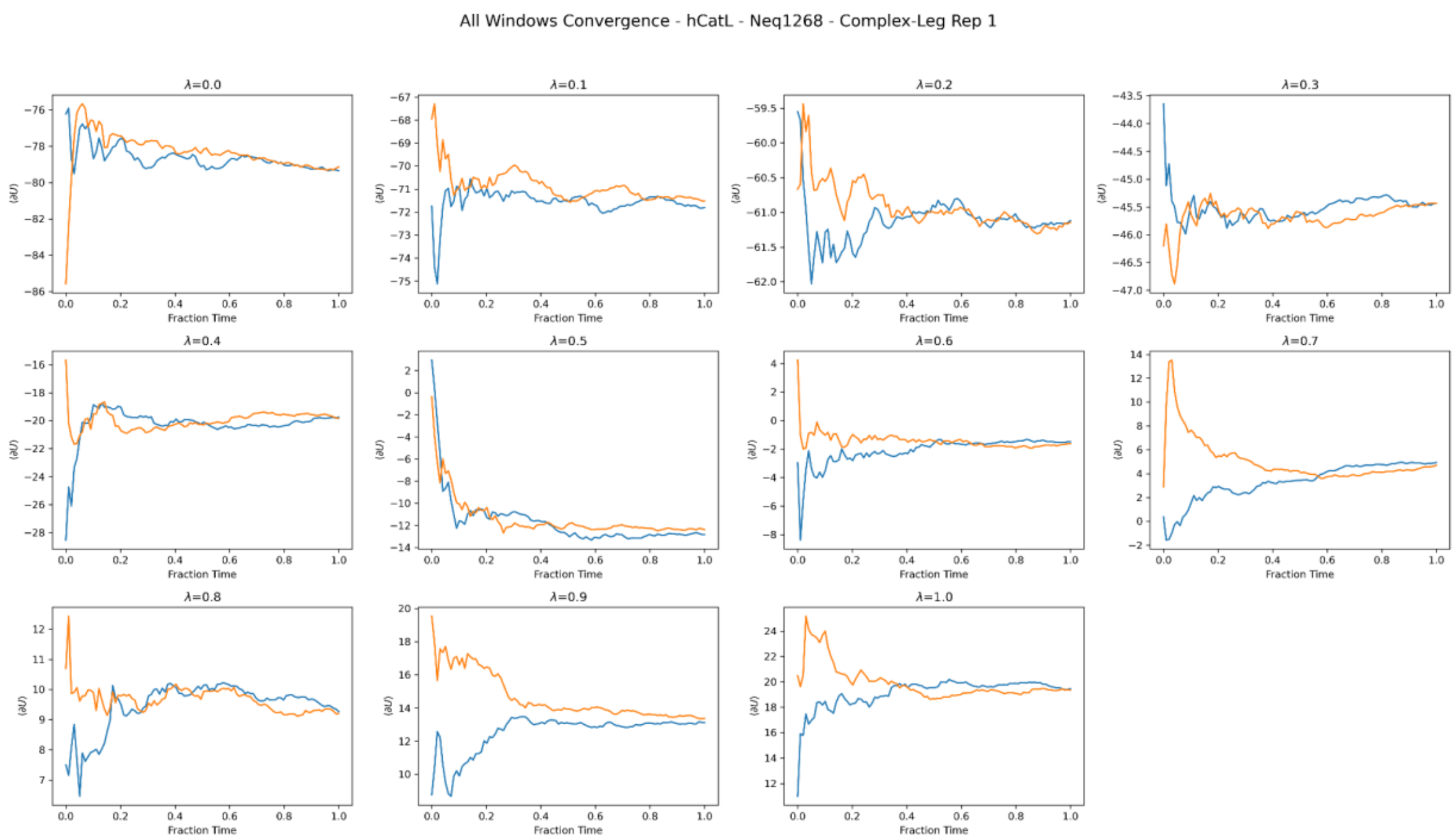

Figure S46. Temporal convergence profile of ⟨∂U/∂λ⟩ across all simulated λ windows in the complex step for the alchemical transformation from hCatL-Neq1268 to hCatL-Neq1276, (replicate 1).

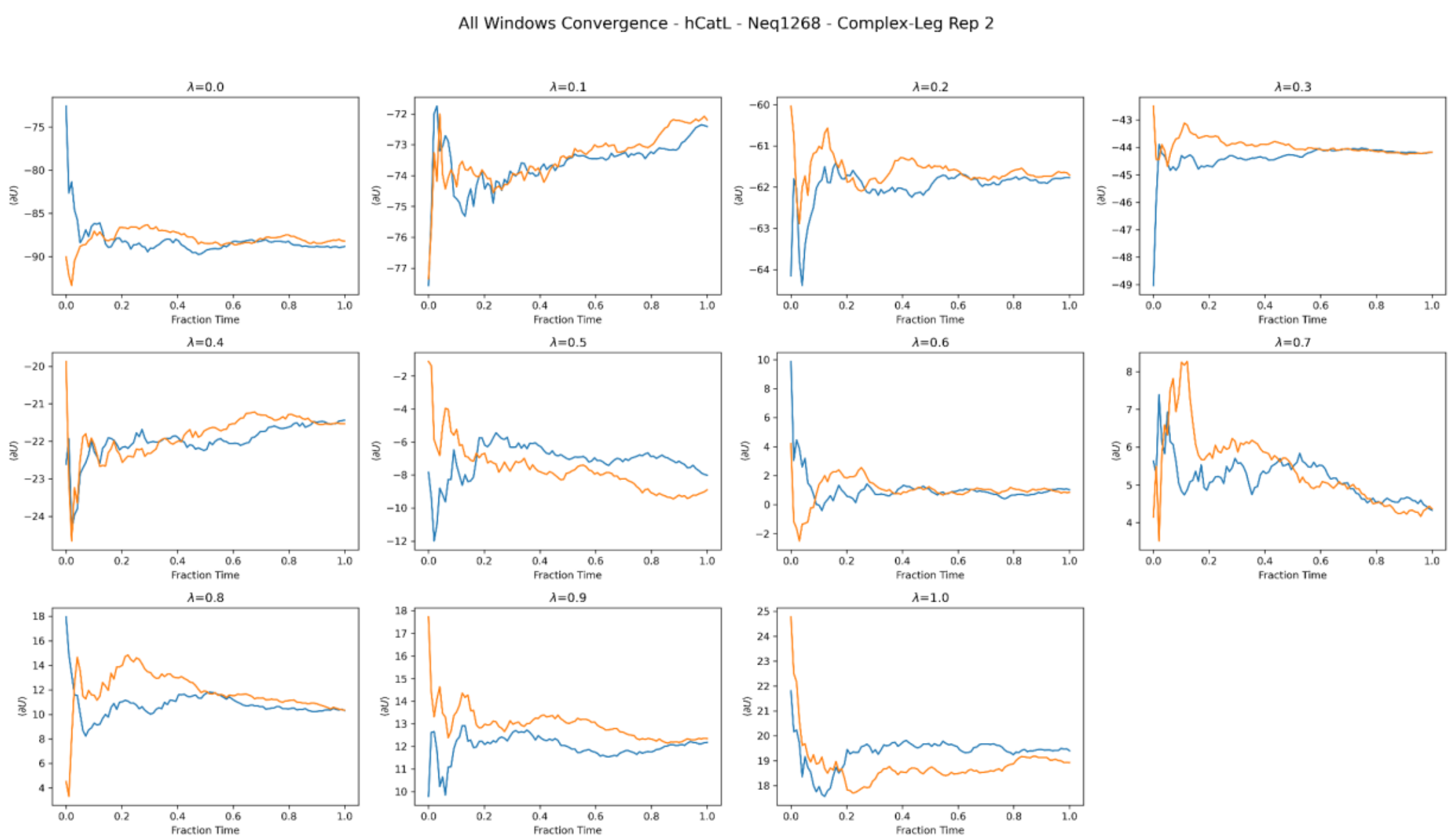

Figure S47. Temporal convergence profile of ⟨∂U/∂λ⟩ across all simulated λ windows in the complex step for the alchemical transformation from hCatL-Neq1268 to hCatL-Neq1276, (replicate 2).

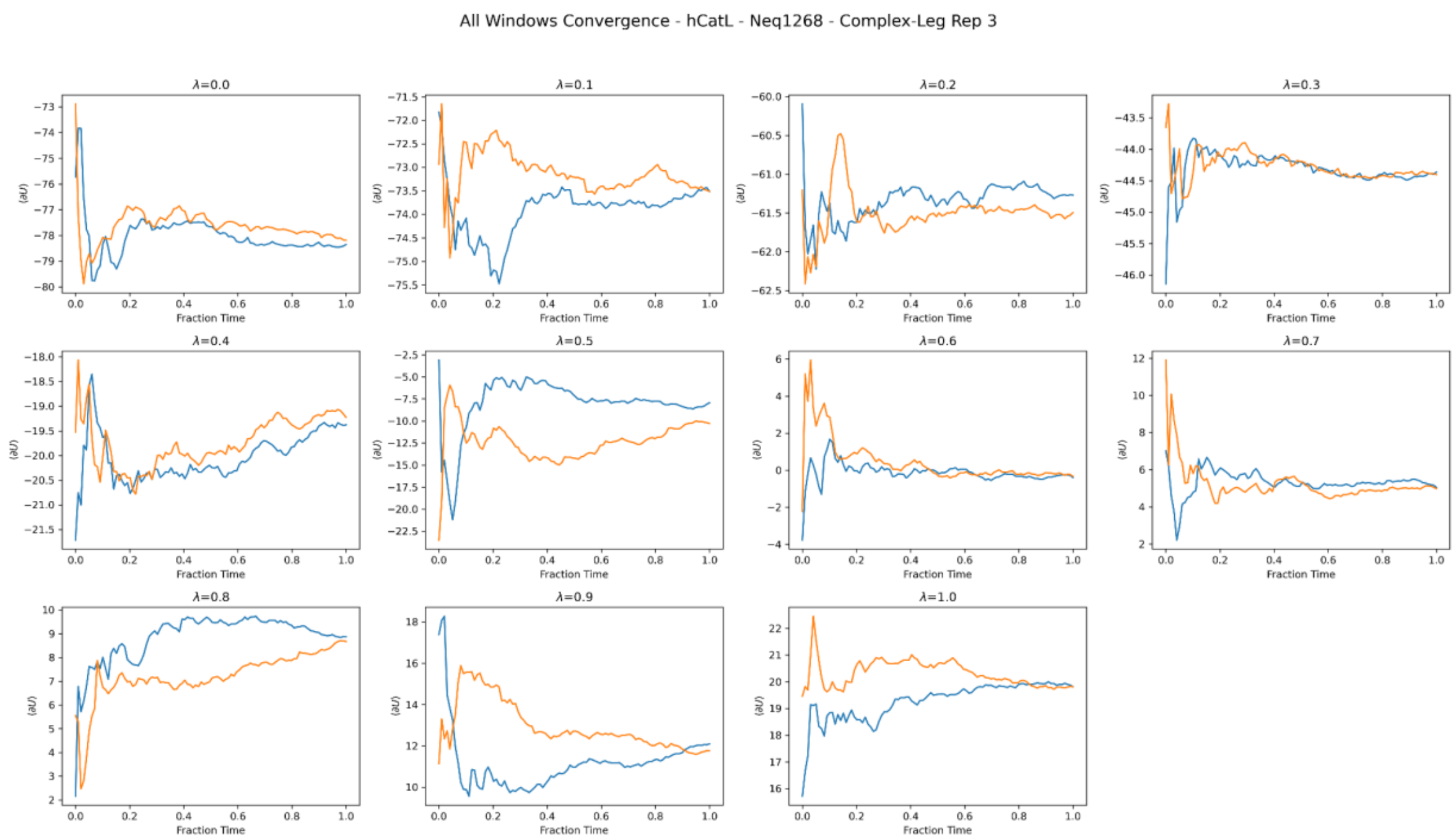

Figure S48. Temporal convergence profile of ⟨∂U/∂λ⟩ across all simulated λ windows in the complex step for the alchemical transformation from hCatL-Neq1268 to hCatL-Neq1276, (replicate 3).

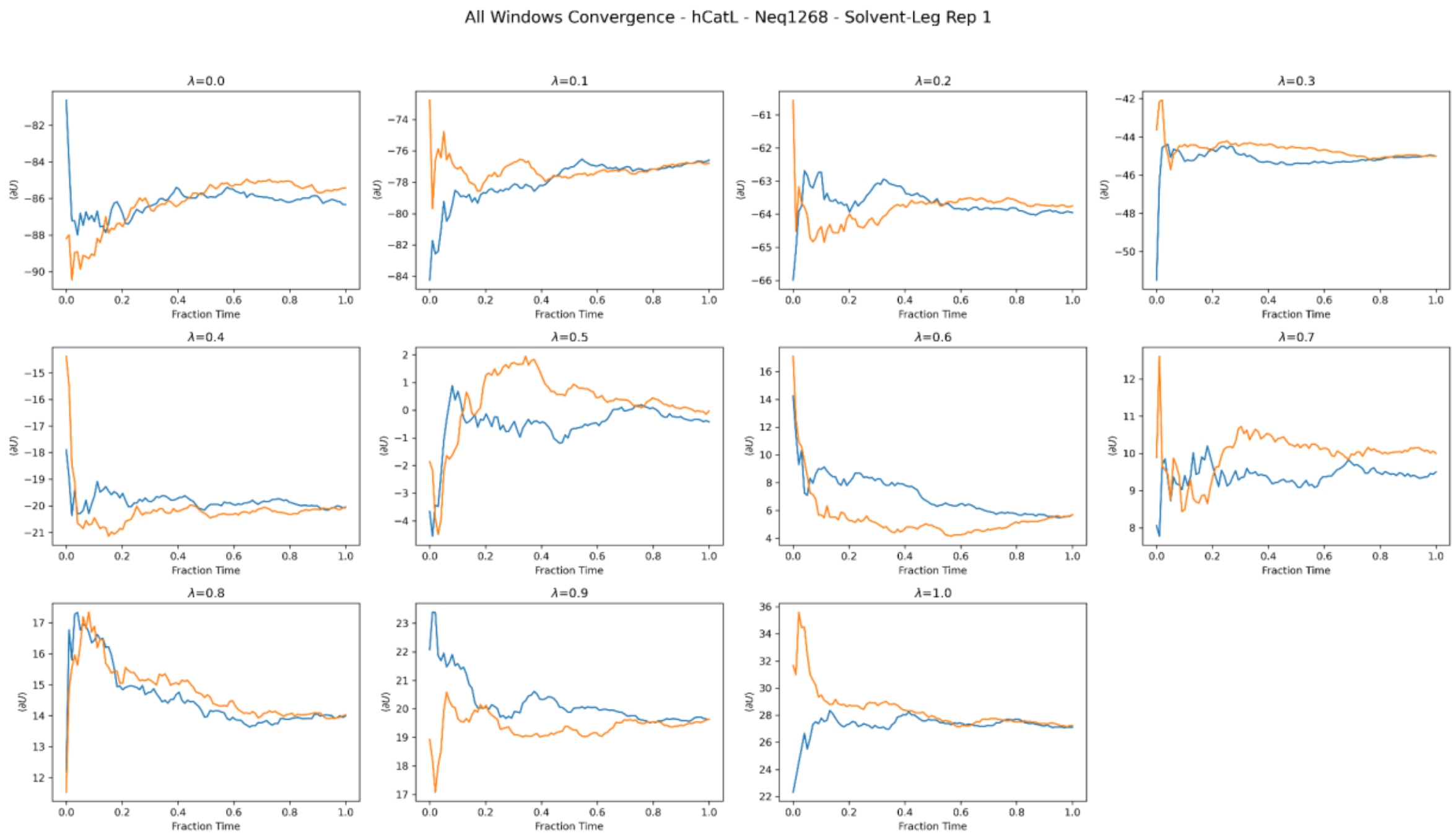

Figure S49. Temporal convergence profile of ⟨∂U/∂λ⟩ across all simulated λ windows in the solvent step for the alchemical transformation from hCatL-Neq1268 to hCatL-Neq1276, (replicate 1).

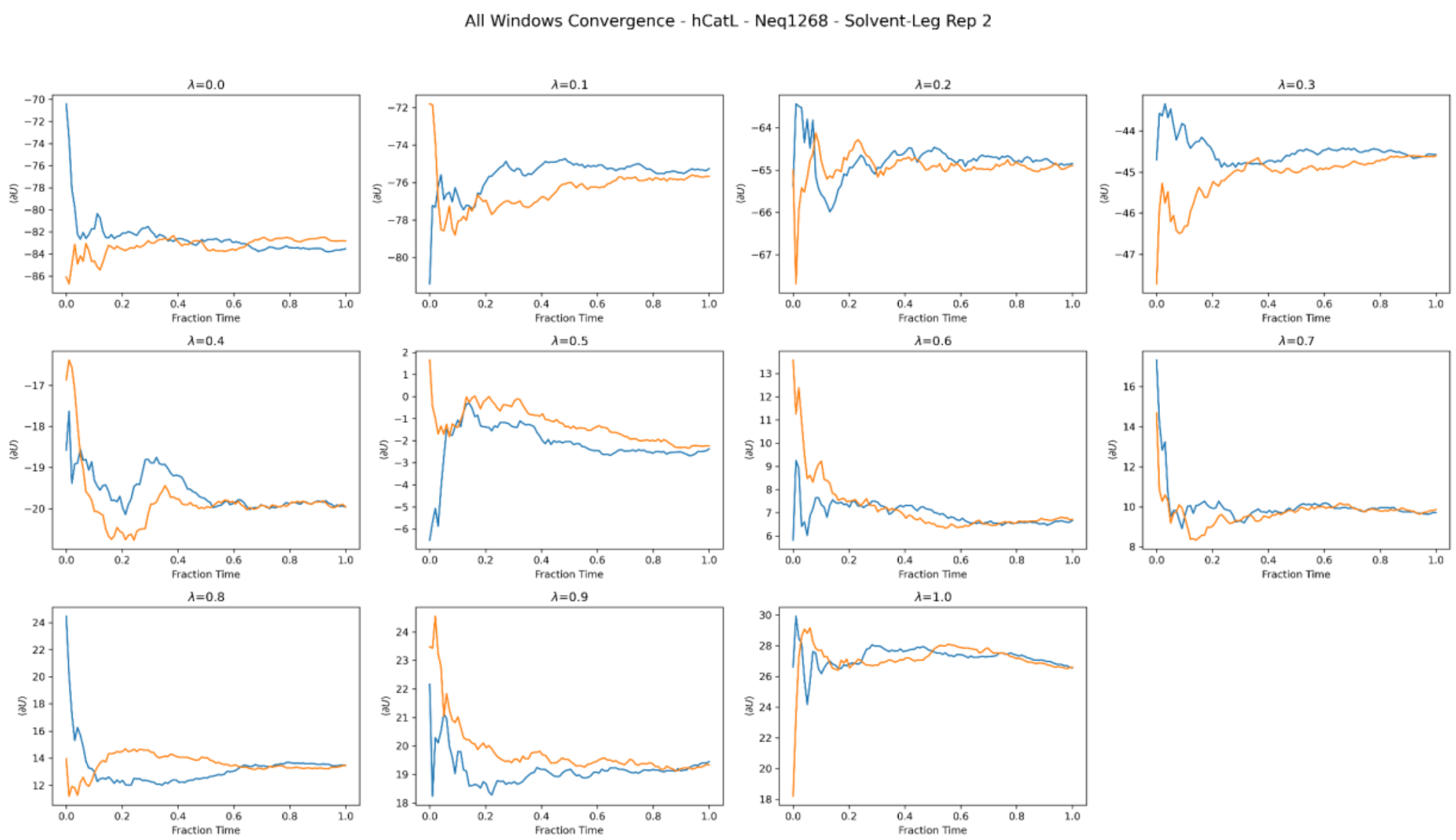

Figure S50. Temporal convergence profile of ⟨∂U/∂λ⟩ across all simulated λ windows in the solvent step for the alchemical transformation from hCatL-Neq1268 to hCatL-Neq1276, (replicate 2).

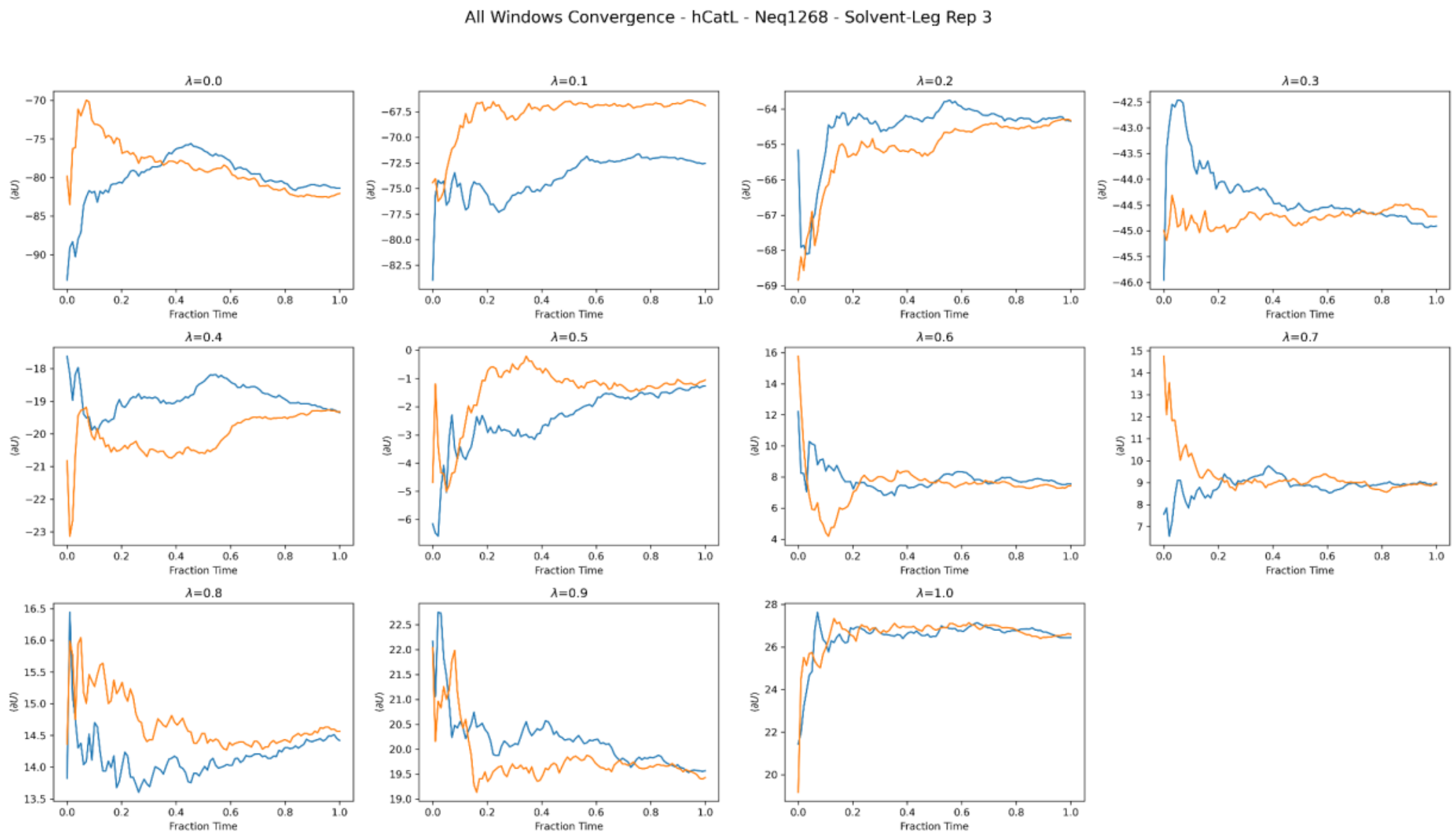

Figure S51. Temporal convergence profile of ⟨∂U/∂λ⟩ across all simulated λ windows in the solvent step for the alchemical transformation from hCatL-Neq1268 to hCatL-Neq1276, (replicate 3).
